# The Assessment of Twitter’s Potential for Outbreak Detection: Avian Influenza Case Study

**DOI:** 10.1038/s41598-019-54388-4

**Published:** 2019-12-03

**Authors:** Samira Yousefinaghani, Rozita Dara, Zvonimir Poljak, Theresa M. Bernardo, Shayan Sharif

**Affiliations:** 1 School of Computer Science, University of Guelph, Guelph, Ontario Canada; 2 Department of Population Medicine, Ontario Veterinary College, University of Guelph, Guelph, Ontario Canada; 3 Department of Pathobiology, University of Guelph, Guelph, Ontario Canada

**Keywords:** Influenza virus, Epidemiology, Computational science

## Abstract

Social media services such as Twitter are valuable sources of information for surveillance systems. A digital syndromic surveillance system has several advantages including its ability to overcome the problem of time delay in traditional surveillance systems. Despite the progress made with using digital syndromic surveillance systems, the possibility of tracking avian influenza (AI) using online sources has not been fully explored. In this study, a Twitter-based data analysis framework was developed to automatically monitor avian influenza outbreaks in a real-time manner. The framework was implemented to find worrisome posts and alerting news on Twitter, filter irrelevant ones, and detect the onset of outbreaks in several countries. The system collected and analyzed over 209,000 posts discussing avian influenza on Twitter from July 2017 to November 2018. We examined the potential of Twitter data to represent the date, severity and virus type of official reports. Furthermore, we investigated whether filtering irrelevant tweets can positively impact the performance of the system. The proposed approach was empirically evaluated using a real-world outbreak-reporting source. We found that 75% of real-world outbreak notifications of AI were identifiable from Twitter. This shows the capability of the system to serve as a complementary approach to official AI reporting methods. Moreover, we observed that one-third of outbreak notifications were reported on Twitter earlier than official reports. This feature could augment traditional surveillance systems and provide a possibility of early detection of outbreaks. This study could potentially provide a first stepping stone for building digital disease outbreak warning systems to assist epidemiologists and animal health professionals in making relevant decisions.

## Introduction

According to the World Health Organization^1^, health surveillance is defined as the continuous and systematic collection, analysis and interpretation of health-related data. Disease surveillance systems can serve as early warning tools for disease outbreaks. These systems have been used for the planning, implementation, and evaluation of health practices^1^. Traditionally, these systems rely on records from health departments^2^. The established formal protocols and regulations in the traditional systems lead to lag times between observations, report submissions and communications. This usually results in delayed and labour-intensive actions performed by health authorities^3–5^. Online services, such as Twitter, are gaining importance as a valuable source of information for decision support systems in healthcare^4,6^. This is because information can be transmitted through social networks faster than traditional channels^4^. Rapid communications taking place on social media can assist in timely reactions by decision makers. Quick responses play a key role in time-sensitive tasks such as controlling epidemics^7^.

The main goal of digital surveillance systems is to monitor and detect potential epidemic events from informal online sources^8^. These systems are used to help health authorities and decision-makers to react promptly to disease emergencies and therefore, reduce or eliminate the consequences. As discussed earlier, these systems can enhance the speed of reactions as they can bypass the formal information channels. Extensive work has examined the value of Internet-based sources such as social media, search engines and news contents for conducting infectious disease surveillance^9–11^. Some studies have focused on confined geographical locations, such as Latin America^12^, the Netherlands^13^ and Nepal^14^, while others have analyzed disease outbreaks globally^15,16^.

The benefits offered by online content for infectious disease surveillance^17,18^ can be compromised by irrelevant content. Sometimes, terms change their meaning in different contexts. For instance, discussions regarding “avian flu dance” on Twitter are irrelevant in the context of avian influenza. Online content might also be generated to propagate advertisements, viruses and phishing, or to enhance systems reputations^19^. Spammers, usually, mask their content to look relevant and useful to users. These messages have often a narrow vocabulary in comparison with the messages that attract the attention of a great number of people^20^. Informal and repeated conversations have been also considered spam in the literature^21,22^. That being said, the identification of relevant data as input for online surveillance systems is necessary^23^. Multiple techniques have been proposed in the literature to disambiguate the context of online media. The techniques range from link ratio calculation^21^ to sentiment analysis and classification methods^22,24^.

Despite significant efforts in the detection of disease events from web content, further research is required. There are still several shortcomings in tracking health-related events on the Internet. Indeed, to the best of our knowledge, limited work exists on the analysis of online content related to infectious disease in animals, in particular, AI^25^. The existing work has mostly focused on seasonal diseases especially^9,11,26–28^. In addition, the semantic purification of online discussions has been overlooked in the literature. Robertson and Yee^25^ developed a surveillance system to analyze AI-related Twitter messages collected during a six-month period. Although the authors found a strong correlation between monthly observations of official reports and tweets, they could not precisely identify the onset of reports. That said, the main contribution of our work consists of introducing a system that enhances previous attempts to identify AI outbreaks from Twitter. The improvement was achieved by constructing location-specific time-series and removing irrelevant content. Another reason why further research on digital surveillance systems for animal diseases is required lies in the significant economic and health impacts that surveillance systems can offer to the poultry industry.

In the present study, we constructed an Internet-based surveillance system that monitors AI activities on Twitter. The main goal of the system was to evaluate the possibility of using Twitter as a complementary source of AI official reports. The proposed system consists of three components: data collection, natural language processing (NLP) and anomaly detection. The data collection component continuously downloaded AI-related tweets using the Twitter search API (application programming interface). The next component, NLP, was used to determine the suitability of tweets for monitoring AI outbreaks. Finally, the third component (i.e. anomaly detection) was designed for country-level analyses to meet four objectives: I) To investigate the potential of Twitter as a supplementary source of AI official reports. II) To examine the impact of sentiment classification of Twitter-derived posts on the accuracy of anomaly detection. III) To identify severity and virus subtypes of outbreaks from Twitter. IV) To investigate the extent to which Twitter content can assist in early warning of AI outbreaks.

## Related Work

Efforts have been made to detect common epidemic events such as seasonal influenza and Influenza-like Illness (ILI) through web and social media^9,11,26–28^. However, challenges in the detection of unexpected outbreak events through social media have not been fully explored. As outlined in the literature^4,15,29,30^, the approaches in the detection of common and recurring health events from web content are mature. These approaches^17,29,31–33^ have measured the strength of the relationship between the frequency of officially reported cases and online disease-related posts or keywords^9,17,34–37^.

Several methods have been exploited to detect disease outbreaks from social media. In the study by Di Martino *et al*.^15^, the Early Aberration Reporting System (EARS) family of algorithms was selected for outbreak detection. In order to validate Twitter alerts, a method was proposed to find relevant official events from ProMED-mail unstructured documents. A document would contain a relevant event if both medical conditions and geographic reference were identified. In another study, van de Belt *et al*.^13^ developed an early outbreak system in the Netherlands using Google Trends and a local social media, named Coosto. In this system, a simple cut-off criterion (i.e. double standard deviation for Google Trends and a frequency above ten for Coosto) was specified to detect outbreaks. In this study, the Dutch outbreak notification system was used as the gold standard. Van de Belt and colleagues^13^ concluded that compared to Google Trends, a limited number of outbreaks were detectable from Coosto. However, the number of false positive detections in Coosto were lower than in Google Trends.

Considering various geographical locations, Fast and colleagues^16^ designed a warning system to identify and forecast social response to diseases reported in news articles. The HealthMap news articles were automatically tagged with indicators of disease spread, severity, preventive measures and social responses. Then, a Bayesian Network and exponentially weighted moving average (EWMA) methods were used to detect unusual periods of social responses. The findings showed that when the news coverage is sufficient, the social reaction to disease spread can be predicted via online news.

As outlined earlier, due to the noisy nature of the content of social media networks such as Twitter, the outbreak detection in these networks is challenging^4,29,30^. In addition to noises, sometimes terms can change their meaning in different contexts. In this regard, a few studies have considered filtering irrelevant contents as part of disease outbreak detection. Di Martino *et al*.^15^ exploited a list of negative keywords associated with diseases to filter irrelevant tweets. In another study by Avare *et al*.^4^, an adaptive classification method based on feature change was proposed to dynamically annotate tweets with relevant or irrelevant labels. In particular^4^, aimed to dynamically change the definition of relevant tweets as terminologies evolve in Twitter messages. The required labels were obtained from health experts and crowd-sourced workers. For incoming tweets, manual labelling was only performed if the extracted features were different than previous features.

## Methodology

**Figure 1:**
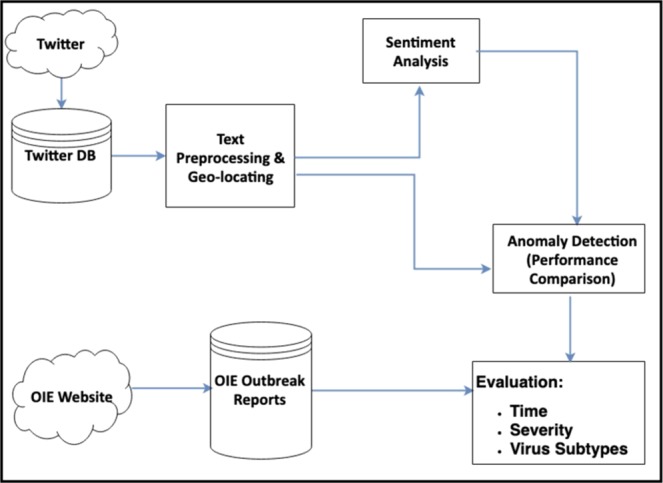
Architecture of the proposed system: Data is collected from Twitter and OIE. After pre-processing, anomalies are detected and evaluated.

The primary goal here is to investigate the extent to which Twitter data can represent actual AI outbreak notifications. Moreover, the potential of Twitter content to assist in AI outbreak-alarming tools is investigated. An overview of the proposed system is given in Fig. 1. The proposed system includes several components. Initially, Twitter posts and OIE outbreak reports were collected and stored in their corresponding databases. The Twitter database was then cleaned and geo-located in the next phase. After specifying labels to tweets in a semi-supervised classification component, irrelevant content was filtered. Next, in the anomaly detection component, the influence of sentiment analysis task on the performance of anomaly detection was investigated. Performance of anomaly detection from tweets was evaluated in terms of time and severity of outbreaks as well as the virus type corresponding with outbreaks. In this component, we looked for sudden changes in the daily time-series of country-based tweets.

### **Data collection and pre-processing**

#### **Social media posts**

The Twitter data-collector component used a crawler, i.e. a PHP script, to visit Twitter every minute. The crawler exploited the Twitter Search API^38^ to download posts and continuously store them into a MySQL database. The Twitter Search API can have near real-time access to a collection of tweets matching a specified query. In the API, we restricted tweets to “english” language and “recent” results. Also, several keywords were fed into the Search API to obtain required tweets. These keywords were extracted from sample tweets associated with AI and confirmed by experts in the field. The returned JavaScript Object Notation (JSON) was parsed into following fields: user ID, user name, user screen name, profile location, number of re-tweets, number of favourite hits, text and date. In total, the collected dataset contained about 209,000 observations generated by over 116,000 unique users from July 2017 to December 2018. Among stored posts, over 105,000 posts were re-tweets (i.e. re-share) of other tweets. Except for an interruption that occurred in April and May 2018 due to server issues, the data collection process has been continuous during the study period. This data collection process ensures that the temporal patterns in the tweets can be representative of users’ activities. Then, re-tweets and non-UTF-8 characters were eliminated from further consideration. In order to use the information effectively, the next challenge was to map the tweets to their associated geographical locations.

To geolocate the collected tweets, a Python library^39,40^ and regular expressions were used. The location details included physical location information such as city, county, state, country, latitude and longitude. We found locations within the context of tweets more precise than geo-location information in the user profiles. About 25% the tweets had empty profile location and 7% had vague location information in the profile field (e.g. earth, worldwide, everywhere, nowhere and international). Since profile locations do not necessarily specify the actual locations corresponding to tweets, we used the context of tweets to find geographical references of events.

#### **Outbreak ground truth**

To validate the surveillance value of the insights obtained from tweets, reports were aggregated from the disease information interface on the OIE website^41^. We utilized a programmed robot to visit the OIE web pages every four hours. The robot filtered and stored AI-relevant reports including 60 immediate and 382 follow-up notifications for the same duration that tweets have been collected. The stored database included the following fields: location, report type, start date, confirmation date, report date, date of submission to OIE, resolve date, number of new outbreaks, the reason for notification, manifestation, agent, serotype, species, number of susceptible, number of deaths, number of disposed, morbidity rate, mortality rate, fatality rate and lost proportion. Moreover, a new field, named “magnitude”, was added to each report entry, indicating the severity of reported outbreaks. The “magnitude” field was assigned with “L”, “M” and “H” codes, respectively showing low, medium and high severities. We categorized reports as high when the ‘number of lost’ was higher than 50,000 animals. Otherwise, when ‘number of lost’ was higher than 5,000 animals, we tagged it with medium severity and when lower, we assigned low severity.

### **Natural language processing module:** positive or negative

The NLP module was used to examine whether a tweet is appropriate to be used in AI surveillance. The terms within the text of tweets can refer to various meanings that might not be in the context of AI. For instance, tweets about “bird flu” song, scientific discoveries, influenza in swine, farm sale and “bird flu” insurance cannot convey information about AI. The noise generated by irrelevant tweets can adversely affect the event detection process and also, enhance the computational costs^42^. Therefore, the process of filtering irrelevant tweets can help in obtaining more accurate detection of outbreaks. Annotation of stream and massive data sources by experts can be expensive and infeasible. That being said, the next challenge to tackle was to perform the automatic annotation of tweets, which is discussed below.

#### Experimental settings

The “relevant” and “irrelevant” labels were manually assigned to about 4,200 sample tweets. Sample tweets were uniquely and randomly selected from all monthly periods of the collected dataset. Generally, it is challenging, even for human experts, to determine whether a tweet is relevant to AI due to limited contextual information within the short-length tweets. Therefore, a set of guidelines were defined to annotate sample tweets with the help of experts in the field.

Tweets were assigned with the “relevant” sentiment, where their content related to official reports of AI farm records, emergencies and outbreak consequences. Moreover, informal reports on individual cases and disease avoidances were considered in the same class label. For example, tweets “mild form of bird flu found in Minnesota turkey flock”, “Why don’t you just stop eating poultry. It is the bird flu” and “Jack got the flu shot last year and still ended up with the bird flu” can show new cases of bird flu. On the other hand, jokes, advertisement and political issues were annotated as “irrelevant”. For example, tweets “Phylogenetic classification of hemagglutinin gene of H9N2”, “Our paper is out! The Cloacal Microbiome of Five Wild Duck Species Varies by Species and Influenza A Virus” and “The bird flu dance is actually very similar to the cat daddy” do not seem to be informative of any AI occurrence. Overall, the hand-labelled corpus of tweets contained 1,647 positive (i.e. relevant) and 2,552 negative sentiments (i.e. irrelevant).

To address the needs of the proposed system for online and automatic labelling of tweets, a semi-supervised Naive Bayes (NB) model was trained on both labelled and unlabelled datasets. In this paper, the semi-supervised learning was performed using the “Sklearn” Python library^43^. The input data was represented in a TF-IDF (Term Frequency-Inverse Document Frequency) vector space. TF-IDF scales the term (word) frequency counts in each document by penalising terms that appear more frequently across multiple documents^44^. The weight is calculated by multiplying two scores: the term frequency (TF) and the inverse document frequency (IDF), which are given in Eqs. 1 and 2. The calculated TF-IDF vectors were then used as input of the Naive Bayesian model explained in the following section.

1 $$TF(t)=\frac{Number\,of\,times\,term\,t\,appears\,in\,a\,document}{Total\,number\,of\,terms\,in\,a\,document}$$

2 $$IDF(t)=lo{g}_{e}\frac{Total\,number\,of\,documents}{Number\,of\,documents\,with\,term\,t\,in\,it}$$

#### Naive Bayesian (NB)

Naive Bayesian (NB) is a simple probabilistic classifier based on Bayes’ theorem with strong independence assumptions between the features^45^. In other words, this classifier assumes that the effect of an attribute value on a given class is independent of the values of the other attributes (class conditional independence). It is made to simplify the computations and for this reason, it is called “naive”. The NB classifier is defined as follows: Given a training set of samples, each represented by an n-dimensional vector $X={x}_{1},{x}_{2},\ldots ,{x}_{n}$ and one class label (${C}_{1},{C}_{2},\ldots ,{C}_{k}$), *X* is predicted to belong to the class *C*_*i*_ if and only if $P({C}_{i}|X) > P({C}_{j}|X)$ for $1\le j\le m,j\ne i$. The class, for which $P({C}_{i}|X)$ is maximized is called the maximum posteriori hypothesis. According to Bayes’ theorem, $P({C}_{i}|X)=\frac{P(X|{C}_{i})P({C}_{i})}{P(X)}$, the term $P(X|{C}_{i})P({C}_{i})$ needs to be maximized as *P*(*X*) is the same for all classes. For easing the computational expenses, it is assumed that given the class labels, the values of the attributes are conditionally independent of one another. Mathematically, this means that $P(X|Ci)\approx {\prod }_{k=1}^{n}\,P({x}_{k}|{C}_{i})$. Therefore, terms $P({x}_{k}|{C}_{i})$ can easily be estimated for each class *C*_*i*_ from the training set^46^.

#### Semi-supervised learning

In the present study, we trained an Expectation-Maximization (EM) based semi-supervised classifier to determine the class label of unlabelled tweets. The NB model was utilized as the base classier in the semi-supervised learning procedure. We selected NB to balance between training time and classification accuracy of the labelling process. The EM algorithm is a class of iterative algorithms that uses an optimization strategy for objective functions. Objective functions can be interpreted as likelihood estimates in the presence of missing data. We used EM in this study as it can be used for probabilistic learning models, such as NB. The EM algorithm pseudo-code is given in Algorithm 1.

The EM algorithm with NB as base classifier was adopted here to annotate data points that were not annotated manually. Initially, the trained supervised model was used to predict the labels of the unlabelled data points. Assuming the expectations of missing labels were true, the entire dataset was used to estimate new parameters. The obtained parameters were then used to create a closer estimation of labels compared to the first guess. The process continued until the algorithm converged on a fixed point. Then, the obtained labelled dataset was fed into an anomaly detection algorithm to identify unusual patterns.

**Algorithm 1:**
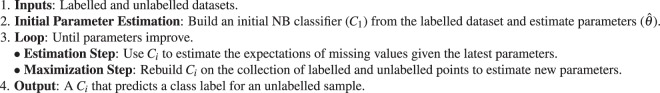
The EM algorithm^47^.

#### **Evaluation**

Initially, a random search was performed to find hyper-parameters that maximize the performance of the semi-supervised model. The list of hyper-parameters tuned on labelled points is given in Table 1.

**Table 1:** Hyper-parameters of training supervised NB.

Hyper Parameter	Tuned Value
Laplace smoothing	0.2
df	(min = 0.001, max = 0.8)

The annotated dataset contained 4,200 instances with 1,647 positive and 2,552 negative labels. In order to manage the processing time and memory required for learning the semi-supervised model on a large amount of data, we divided unlabelled data into seven equal parts, each with about 28,000 instances. Here, an instance is a single row of tweets dataset, a part refers to 28,000 instances and a fold refers to 420 instances. Then, we applied semi-supervised learning to each part and obtained the average test performances using 10-fold cross-validation. Finally, we averaged measures over all parts and reported four evaluation metrics. To evaluate the model, initially, nine train folds were used to train the classifier. Then, the trained classifier was used to learn the classes of unlabelled data. As explained in Algorithm 1, the final classifier was iteratively trained on the union of unlabelled data and train folds. This classifier was ultimately used to classify the test fold. Test evaluation measures with 95% confidence interval are reported in Table 2.

**Table 2:** Semi-supervised test measures.

Measure	Value
Average accuracy	78.40% ± 1.23%
Average precision	79.80% ± 1.21%
Average recall	74.70% ± 1.31%
Average F-score	75.2% ± 1.3%

The precision (Positive Predictive Value) represents how many selected cases are relevant, while the recall (sensitivity) represents how many relevant cases are selected (Eqs. 4 and 5). The F-measure (Eq. 6) is a weighted average of the precision and recall. Finally, the accuracy (Eq. 3) is defined as a statistical measure of how well a binary classification performs in predicting the true results of both classes among the total number of instances. For two-class classifications, there are four possible cases: For a positive class, if the prediction is positive, this is a called a true positive (TP) and if negative, it is a false negative (FN). For a negative example, if the prediction is negative, it is called true negative (TN) and if positive, it is a false positive (FP). We also denoted the number of positive and negative data points with ‘P’ and ‘N’, respectively.

3 $$Accuracy\,(ACC)=\frac{(TP+TN)}{(P+N)}$$

4 $$Precision\,or\,Positive\,Predictive\,Value\,(PPV)=\frac{TP}{(TP+FP)}$$

5 $$Recall\,or\,Sensitivity=\frac{TP}{(TP+FN)}$$

6 $$F-measure=\frac{2\,\ast \,Precision\,\ast \,Recall}{Precision+Recall}$$

With regards to evaluation results presented in Table 2, the semi-supervised learning obtained the accuracy and F-score of 78.4% and 75.2%, respectively. The data points annotated with “relevant” labels were then used in the experiments to understand how using relevant data points instead of the entire relevant and irrelevant data points can improve the performance of anomaly detection.

### Outbreak detection

Here, we aimed to show how reliable it is to use Twitter for AI surveillance purposes. More precisely, we intended (1) to describe the overlap between Twitter daily posts and official reports; (2) to understand the extent to which Twitter can be used for the early warning of AI outbreaks; (3) to indicate whether the size of outbreaks and the virus subtypes involved in outbreaks are detectable from tweets; and (4) to study the impact of filtering irrelevant tweets on the anomaly detection process. To this end, first, we visually demonstrated the overlap between OIE reports and the number of tweets. Then, the similarities between the time-series of outbreaks and tweets in terms of the onset date of reports, the magnitude of outbreaks and the type of virus involved in outbreaks were examined. Finally, the anomaly detection was applied to the original and filtered dataset and the results were compared. In this study, original tweets refer to all collected tweets before discarding irrelevant ones.

Anomaly detection is an essential application area of time-series data^48^. Anomaly detection processes in time-series are usually formulated as identifying outliers or unusual data points relative to some standard or usual signals^48,49^. Anomalies may be due to (1) inherent variabilities or (2) errors in data. Anomalies in the former category, which was considered in this study, contain valuable information that is useful for decision-making tasks^48^. The Seasonal-Hybrid Extreme Studentized Deviate (SH-ESD) algorithm was incorporated in the proposed system to detect the onset of outbreaks from time-series of AI-related tweets. SH-ESD algorithm was specifically designed for anomaly detection in the cloud infrastructure data with the velocity, volume, and real-time nature^50^.

#### SH-ESD algorithm

SH-ESD algorithm is a robust and recently-published anomaly detection technique which is built upon the Generalized ESD test^51^. SH-ESD can be used to detect both global (i.e. variations that cannot be explained with seasonal patterns) and local anomalies (i.e. variations inside seasonal patterns)^51^. The algorithm is composed of a statistical test hypothesis called Grubb’s Test and a time-series decomposition method, known as Seasonal-Trend Decomposition based on Loess (STL). First, STL decomposition is used to extract the residual component of the observed data. The residual is assumed to be symmetrically distributed (i.e. normal distribution). Then, Grubb’s Test finds outliers in a sample of residuals, which is assumed to be normally distributed^48^.

#### Experimental Settings

To prepare the required data, a continuous time-series of the daily number of tweets was calculated for each country during a 15-month period. Additionally, the OIE database was queried to obtain dates on which immediate outbreak notifications were reported for each country. To detect anomalies given time-series of tweets, we used an open-source R package “AnomalyDetection” that was released by the Twitter engineering team^52^. This package uses the SH-ESD technique, which was explained earlier. We identified anomalies from both original and relevant tweets, i.e. those classified as relevant, and compared the result. The comparison was performed to examine whether relevant tweets can be a better representative of outbreak notifications compared to original tweets.

## Results and Discussion

### Overlap visualization

**Figure 2:**
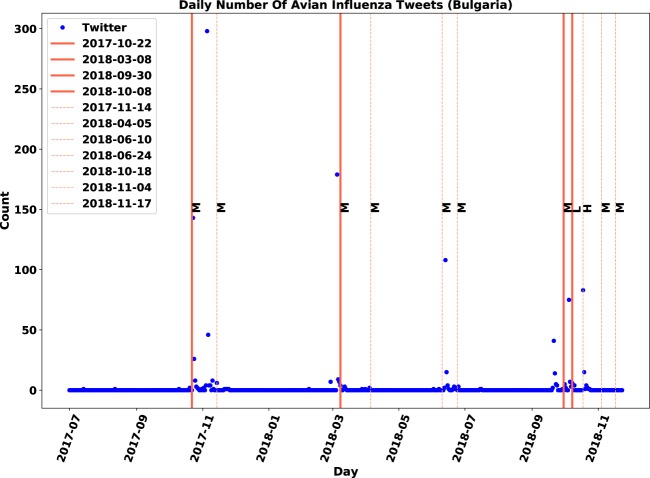
Overlap visualization of Twitter and official outbreak reports in Bulgaria: Twitter daily count data is marked by blue points, immediate outbreak reports by red lines and follow-up outbreak reports by dash lines.

**Figure 3:**
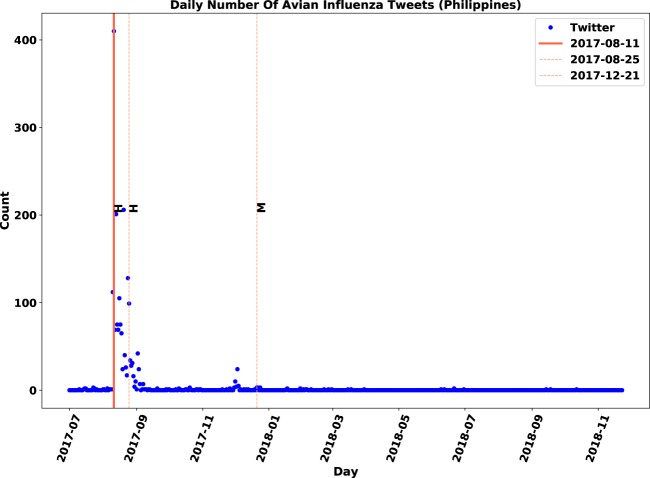
Overlap visualization of Twitter and official outbreak reports in Philippines: Twitter daily count data is marked by blue points, immediate outbreak reports by red lines and follow-up outbreak reports by dash lines.

The overlap patterns between time-series of AI-related tweets and outbreak reports were plotted for all 21 locations. The patterns for Bulgaria and the Philippines are displayed in Figs. 2 and 3, respectively. The patterns for the rest of locations can be seen in Figs. 1–21 in Supplementary Document. Visualizations were drawn from the tweets that were annotated with “relevant” label in the database. The time-series of tweets are marked with circular blue points and the sporadic outbreak reports during the course of study with vertical red lines. Beside each line, a letter (i.e. L, M and H) indicates the severity of outbreaks. Generally, a surge in the number of tweets around report dates defines an existing pattern.

As shown in Figs. 2 and 3, the distribution of the continuous and discrete time-series related to Bulgaria was similar. The first peak in the number of tweets took place in October 2017, when an immediate report was announced on the same day. After that, the number of tweets increased two-fold (i.e. 300 tweets) in early November. This seemed to be associated with a follow-up report 20 days after the first immediate report. Similarly, follow-up reports might be a reason behind the rise of tweets in mid-June and mid-October. Also, it is worth noting that in all three outbreaks reported in 2018, discussions on Twitter started to rise a few days before being officially reported (Fig. 2). Overall, the patterns observed in messages discussing AI events in Bulgaria showed a connection with the official reports.

Correlation patterns for the Philippines are shown in Fig. 3. A single outbreak was reported in August 2017, which was reflected on Twitter with a sudden rise followed by a gradual downward trend. The observed pattern is interesting as the activity grew a day before the immediate report, reached a peak at around 400 tweets on the report day and then, gradually dropped within 15 days. The trends of the Philippines, therefore, are explainable by Twitter activities.

Similar to observations in the Philippines, there was only one immediate outbreak notification during the study period for Korea, Malaysia, Ghana, Myanmar, Ireland, Iran, Pakistan and Saudi Arabia. Except for Ireland, the Twitter activity regarding these countries reached its highest value at the same time the corresponding outbreak was reported. For Ireland, however, Twitter activity on the notification day had the second highest value. In the observations with one immediate report, the periods of downward trends after immediate notifications were approximately 10–30 days. The upward trends before outbreaks, however, were sudden and shorter in length compared to the downward trends.

Several patterns in the observations could not be interpreted by considering immediate reports alone. For instance, the surge in the number of tweets in early October regarding Italy was associated with a follow-up report of three severe outbreaks, i.e. with magnitude code of “H”. Similarly, the second highest peaks of Korea and the Philippines were interpretable by follow-up reports. Furthermore, the peaks in Twitter activity in July and August regarding South Africa, early June regarding Bulgaria and mid-October 2017 regarding China seemed to be connected with follow-up reports. In these time-series, the activities jumped a few days before or after follow-up reports. Therefore, in addition to immediate reports, the impact of follow-up reports, in particular, the follow-up reports of severe outbreaks, could be important.

### Event detection

Given the country-based historical time-series of tweets pertaining to AI, an anomaly detection algorithm (i.e. SH-ESD) was used to identify the spikes. Confusion matrices and evaluation measures were calculated by comparing detected points (i.e. anomalies) with the actual reports. As a reference standard of notifications, we considered ‘report date’ field from the collected OIE dataset. A detected anomaly point would be considered true positive (TP) if it occurred within a 10-day window around immediate notifications. If an anomaly could not be found within any window of immediate notifications, it would be a false positive (FP). On the other hand, an immediate notification that has had no spike around it was considered as false negative (FN). The locations having less than ten daily tweets during the study period were discarded from further analyses. Also, we did not consider countries with no immediate notification during the period of study. The obtained evaluation measures including precision, recall and F-measure for 21 locations are given in Table 3.

**Table 3:** Evaluation measures of anomaly detection.

Country	TP	FP	FN	Precision	Recall	F-score
Bulgaria	4	2	0	67%	100%	80%
China	4	2	5	67%	44%	53%
France	1	3	1	33%	100%	33%
Germany	4	0	2	100%	67%	80%
Ghana	1	1	0	50%	100%	67%
India	3	1	0	75%	100%	86%
Iran	1	0	0	100%	100%	100%
Ireland	1	2	0	33%	100%	50%
Japan	2	1	0	67%	100%	80%
Korea	1	3	0	25%	100%	40%
Myanmar	1	0	0	100%	100%	100%
Malaysia	1	0	0	100%	100%	100%
Netherlands	3	0	2	100%	60%	75%
Pakistan	0	0	1	0%	0%	—
Philippines	1	1	0	50%	100%	67%
Russia	3	0	0	100%	100%	100%
Saudi Arabia	1	0	0	100%	100%	100%
South Africa	2	3	0	40%	100%	57%
Taiwan	2	3	2	40%	50%	45%
USA	4	1	0	80%	100%	89%
Vietnam	3	0	2	100%	60%	75%

Overall, the anomaly detection component was able to identify 43 out of 58 immediate reports, whereas 23 points were identified as anomalies with no corresponding immediate outbreak reports. The 100% recall was obtained for 15 locations, which means all corresponding anomalies were identified. This, however, did not apply to China and Taiwan. There were multiple immediate notifications within a short time frame in China, e.g. October 2018, which made the anomaly detection difficult. In Taiwan, the number of delivered messages was not high enough (i.e. maximum 15) to be able to detect all the events. Overall, the precision measure showed lower values than recall measure. Although eight rows were free of false identifications, five rows showed a precision less than 50%. F-measure showed a quite high value in the majority of locations. Precisely, in 14 out of 21 countries (i.e. about 70%), F-measure was higher than 66%, which implied a significant correlation between Twitter activities and OIE reports.

False positives were most frequently seen in the observations of France, Taiwan, Korea and South Africa. False positives were observed in the time-series of France in Winter 2018, which coincided with two follow-up reports in February and March 2018. We manually looked at messages regarding Korea and discovered that the discussions in July and mid-August were regarding “the reduction of bird flu” and “banning the imports of eggs and poultry from the USA”, respectively. A jump in the discussions on January 29, 2018, might be reasonable as it is associated with a follow-up report of a severe outbreak on the same date. Similarly, a possible explanation for three false positives observed in the time-series of South Africa could be the burst of follow-up reports during the period between August and November.

In several cases, we were not able to explain false positives by either biases or the presence of follow-up reports. The corresponding messages in these points were actually informing about existing cases of bird flu that had been reported on Reuters news. For instance, peaks at late March and mid-June 2018 regarding Ireland were related to cases of H5N6 in wild birds that have not been reported by the OIE. Also, the spike detected in mid-October 2017 by our study for China was associated with an outbreak reported by Reuters. Similarly, an outbreak of H9N2 in Ghana has not been reported by the OIE. H9 viruses are not recognized to have the potential for being highly pathogenic. This might be the reason why the outbreak in Ghana has not been reported by the OIE. Our investigation also indicated that a false positive detection for China at the end of February 2018, i.e. the peak in the time-series of China, was due to a human case of H7N4 bird flu in Hong Kong. Therefore, the proposed work can assist traditional systems in reporting outbreaks.

**Figure 4:**
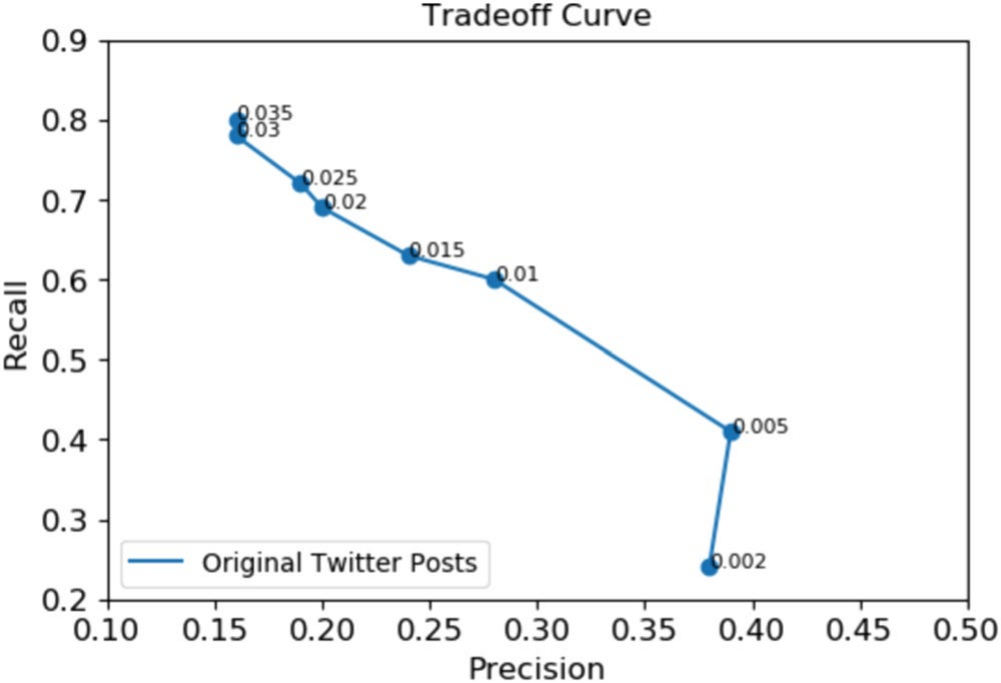
Precision-recall tradeoff curve for overall performance of anomaly detection.

Ideally, it is expected that the proposed approach can find all and only outbreaks. In reality, however, these two desires tend to trade against each other. A precision-recall (PR) curve was visualized in Fig. 4 to indicate the overall performance of the approach presented in the current paper. Here, precision indicates how well we were able to identify only anomalies, whereas recall shows how well we identified all anomalies. The PR curve was parametrized by a threshold representing the number of anomalies to visualize the tradeoff between precision and recall. We used a list of thresholds ranging from 0.002 to 0.35 corresponding with 2–17 anomalies. As illustrated, when we increased the threshold, the precision tended to drop, except for an enhancement that occurred in the plot associated with original tweets when the threshold jumped from 0.002 to 0.0005.

Depending on the application of surveillance systems, we might decide that recall is more important than precision or vise versa. If recall is more important than precision, we will choose higher thresholds for the anomaly detection algorithm. In this case, surveillance systems need to capture most or all anomalies, even if that means dealing with some false alarms. On the other hand, when higher precision than recall is preferred, the selection of lower threshold values is preferred. Overall, to determine the appropriate threshold, the relative costs of false alarms and missing anomalies need to be assessed.

### **Reflection of magnitude of outbreaks onTwitter**

The OIE provides information on the number of susceptible animals, lost, deaths, disposed and mortality rate indicating severity of outbreaks. Given this information, we tagged reports with low, medium and high severities using Eq. 7. For each tagged report, we obtained several statistics for tweets associated with that report.

The information in Table 4 indicates that the magnitude of reported outbreaks is associated with the number of tweets. The majority of outbreaks had low magnitudes with a maximum of 160 associated tweets, whereas the maximum number of tweets for high-severity outbreaks was 390. In addition, as the magnitude increased, centrality measures (i.e. mean, median and mode) increased as well. The average number of tweets associated with low-magnitude reports was 8.41 while for medium and high magnitudes, it jumped to 30.81 and 71.52, respectively.

**Table 4:** Connection between magnitude of reported outbreaks and statistical measures of tweets.

	Low	Medium	High
Count	207	65	21
Min	0	0	0
Max	160	250	390
Mean	8.41	30.81	71.52
Median	1	3	20
Mode	0	0	20

We categorized the severity of outbreaks based on the proportion of animals lost. However, the severity of outbreaks could depend on other factors such as the duration of outbreaks, virulence of viruses and immune status of poultry. In the present work, we assume that severity depends on the limited information accessible from the OIE dataset. This said, our observations revealed that the severity of outbreaks can be determined by Twitter activity.

7 $$number\,of\,lost=susceptibles\,\ast \,proportion\,of\,susceptible\,lost$$

**Figure 5:**
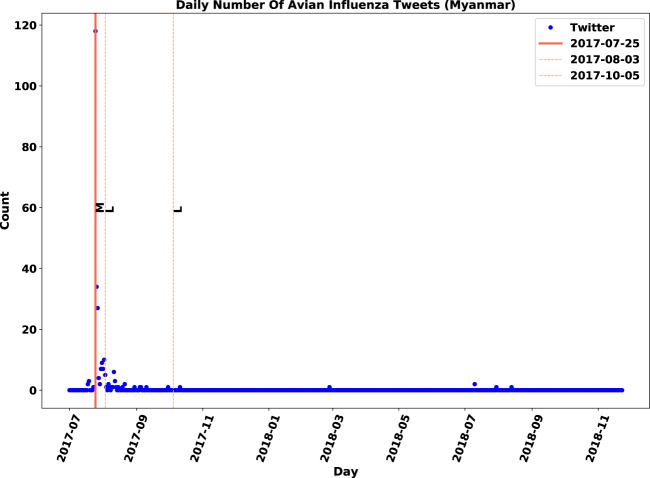
Overlap visualization of Twitter and official outbreak reports in Myanmar: Twitter daily count data is marked by blue points, immediate outbreak reports by red lines and follow-up outbreak reports by dash lines.

**Figure 6:**
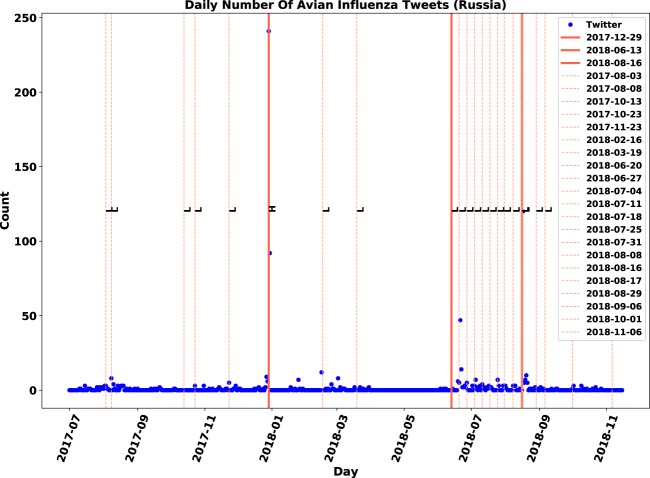
Overlap visualization of Twitter and official outbreak reports in Russia. Twitter daily count data is marked by blue points, immediate outbreak reports by red lines and follow-up outbreak reports by dash lines.

Moreover, we visually illustrated the link between the magnitude of outbreaks and the number of discussions on Twitter. In Fig. 5, low-magnitude outbreaks were reported in early August 2017 and early October 2017, when zero or a very limited number of tweets were communicated. In contrast, in late July, an outbreak with medium magnitude was reported, which led to 120 posts on Twitter. The same patterns can be found in Fig. 6, when 240 posts on Twitter were associated with a high-magnitude outbreak.

### **Subtype identification**

We investigated the possibility of identifying virus subtypes associated with each outbreak from Twitter content. To this end, topic models were built using tweets within a window size of five days around outbreak dates. Latent Dirichlet Allocation^53^, which is one of the most popular topic models was used in the current research. This model assumes that documents are a mixture of topics and a topic is a probability distribution of words. Here, we assumed that there is only one topic in each document. The six most probable words (W1–W6) and their corresponding probability in the topic for a number of immediate reports are given in Table 5. Immediate reports are specified in the table by their related location and subtype.

**Table 5:** High-probability words for immediate outbreak reports.

Outbreaks	W1	W2	W3	W4	W5	W6
Germany (H5N6)	**h5n6** 0.107	confirms 0.101	farm 0.098	case 0.096	island 0.095	**germany** 0.021
China (H5N6)	highly 0.074	reports 0.073	pathogenic 0.053	**china** 0.053	**h5n6** 0.052	outbreak 0.044
Iran (H5N6)	**h5n6** 0.089	wild 0.089	north 0.089	ducks 0.089	among 0.088	reports 0.085
Netherlands (H5N6)	outbreak 0.064	pathogen 0.058	highly 0.056	**h5n6** 0.053	confirms 0.050	**netherlands** 0.033
Russia (H5N2)	**h5n2** 0.078	reports 0.069	**russia** 0.051	outbreak 0.04	farm 0.044	pathogenic 0.042
India (H5N8)	**h5n8** 0.068	highly 0.063	**karnataka** 0.063	virus 0.042	contagious 0.037	positive 0.031
Vietnam (H5N1)	outbreak 0.107	**h5n1** 0.101	reports 0.092	north 0.092	oie 0.073	**vietnam** 0.053
France (H7N7)	**france** 0.073	pathogen 0.073	**h7n7** 0.073	outbreak 0.073	one 0.073	poultry 0.073

**Figure 7:**
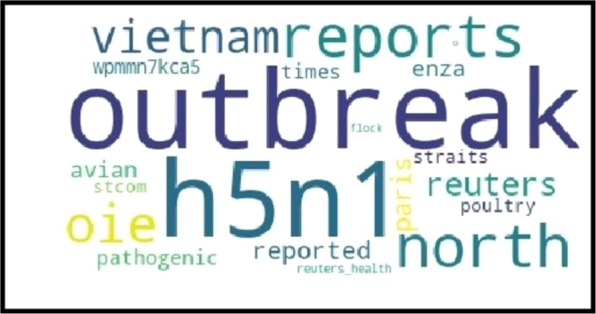
The word cloud around an outbreak date for Vietnam: The size of words is proportional to their probability in the corresponding topic distribution.

**Figure 8:**
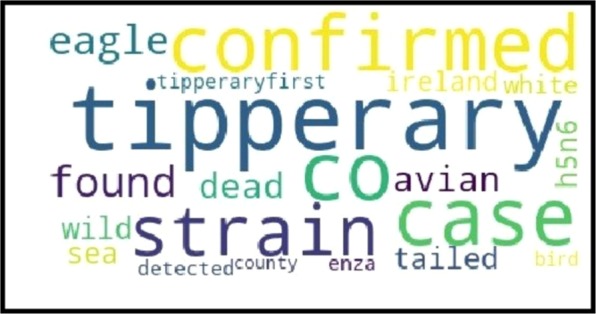
The word cloud around an outbreak date for Japan: The size of words is proportional to their probability in the corresponding topic distribution.

Word clouds for Vietnam and Japan are illustrated in Figs. 7 and 8, where the size of words is proportional to their probability in the corresponding topic distribution. The rest of word clouds can be seen in Figs. 22–77 in the Supplementary Document. Our observations showed that subtype terms were successfully identifiable in 47 out of 58 word clouds. In addition, the location of outbreaks was recognizable in 51 out of 58 word clouds. Our observation showed that the subtypes and locations found in the word clouds were matching the subtype and location of their corresponding official reports. The observed locations and AI virus subtypes involved in outbreaks are highlighted with bold font in Table 5.

Moreover, we identified all existing subtypes and their pathogenicity from the word clouds during the study period. Information on subtypes, their corresponding locations and whether they are of high or low pathogenicity is shown in Table 6. As demonstrated, the majority of discussions around immediate reports were regarding outbreaks with highly pathogenic subtypes. Low pathogenic subtypes have appeared in discussions regarding outbreaks in France, Germany and USA. A few subtypes, e.g. H5N1, appeared in some discussions as highly pathogenic and in others as low pathogenic.

**Table 6:** List of identified subtypes associated with immediate outbreak reports.

Subtype	Location	Pathogenicity
H5N8	Bulgaria/Germany/India/Pakistan/South Africa/Saudi Arabia	high
H5N6	China/The Netherlands	high
H7N9	China	high
H5N1	China/India/Myanmar/Malaysia/Vietnam	high
H5N1	France	low
H5N5	France	low
H7N7	France	low
H5N2	Germany/USA	low
H5N2	Russia	high
H7N1	USA	low
H7N3	USA	low

The topic model can contribute to the surveillance system by introducing the subtype and location of outbreaks. This can be helpful in decreasing the number of false positives and false negatives. It means, if we use word clouds in conjunction with the anomaly detection algorithm, we might be able to detect more outbreaks compared with applying only the anomaly detection algorithm. In addition, word clouds can be used to evaluate detected outbreaks by the anomaly detection method.

### **Contribution of sentiment classification**

We examined the impact of sentiment classification task on the performance of anomaly detection. The anomaly detection algorithm was applied to both original and relevant datasets of tweets and then, the results were compared. The evaluation measures are displayed in Table 7. Although both signals were correlated with the gold standard data, the relevant signal improved the F-score for nine out of 21 locations. We observed that discarding irrelevant posts decreased the number of false positives by 12 and increased the number of true positives by three. The number of false positives dropped from 23 to 11, which means the sentiment filtering of irrelevant tweets helped in discarding approximately half of anomalies that were falsely detected.

**Table 7:** Influence of relevance analysis on evaluation measures of anomaly detection.

	Country	TP	FP	FN	Accuracy	Precision	Recall	F-score
Original	China	3	3	6	33%	50%	33%	40%
Relevant	China	4	2	5	44%	67%	44%	**53%**
Original	France	0	5	2	0%	0%	0%	—
Relevant	France	1	3	1	50%	33%	100%	**33%**
Original	India	0	3	3	0%	0%	0%	—
Relevant	India	3	1	0	100%	75%	100%	**86%**
Original	Japan	2	2	0	100%	50%	100%	67%
Relevant	Japan	2	1	0	100%	67%	100%	**80%**
Original	Netherlands	3	1	2	60%	75%	60%	67%
Relevant	Netherlands	3	0	2	60%	100%	60%	**75%**
Original	Pakistan	0	1	1	0%	0%	0%	—
Relevant	Pakistan	0	**0**	1	0%	0%	0%	—
Original	Saudi Arabia	1	1	0	100%	50%	100%	67%
Relevant	Saudi Arabia	1	0	0	100%	100%	100%	**100%**
Original	Taiwan	2	4	2	50%	33%	50%	40%
Relevant	Taiwan	2	3	2	50%	40%	50%	**45%**
Original	USA	4	2	0	100%	67%	100%	80%
Relevant	USA	4	1	0	100%	80%	100%	**89%**

**Figure 9:**
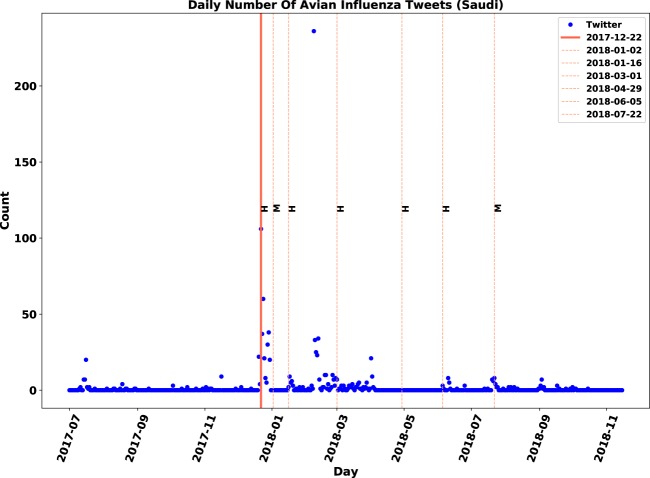
Original daily number of tweets in Saudi Arabia. Twitter daily count data is marked by blue points, immediate outbreak reports by red lines and follow-up outbreak reports by dash lines.

**Figure 10:**
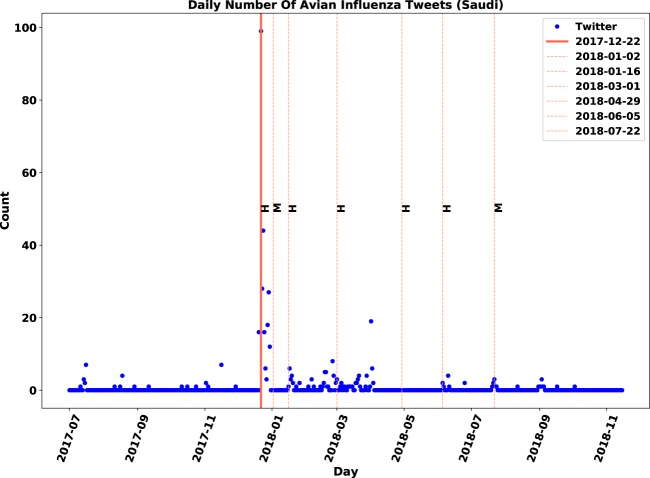
Relevant daily number of tweets in Saudi Arabia: Twitter daily count data is marked by blue points, immediate outbreak reports by red lines and follow-up outbreak reports by dash lines.

**Figure 11:**
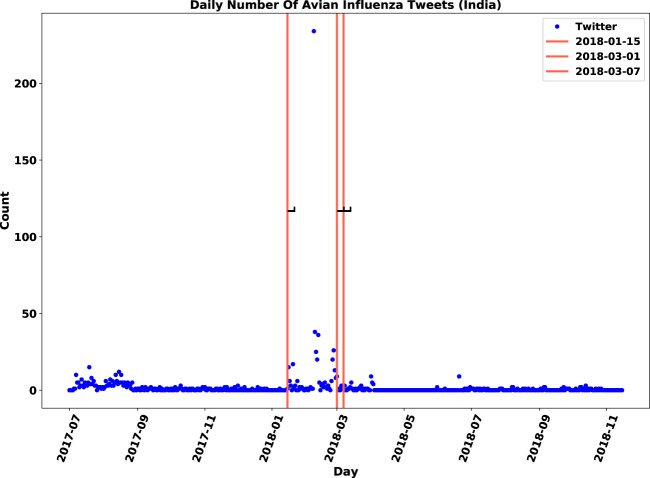
Original daily number of tweets in India: Twitter daily count data is marked by blue points, immediate outbreak reports by red lines and follow-up outbreak reports by dash lines.

**Figure 12:**
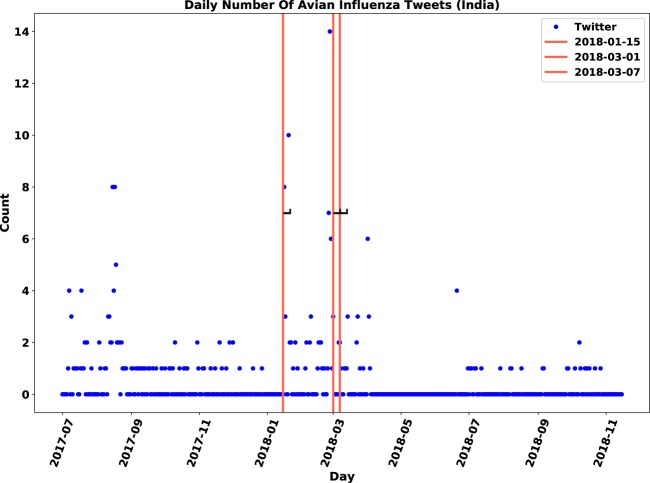
Relevant daily number of tweets in India: Twitter daily count data is marked by blue points, immediate outbreak reports by red lines and follow-up outbreak reports by dash lines.

Comparisons between relevant and original time-series for Saudi Arabia and India are demonstrated in Figs. 9–12. In Figs. 9 and 10, a peak in early February 2018 that had no association with any outbreaks disappeared after the filtering of irrelevant tweets. Conversely, the removal of the peak point in mid-February (Figs. 11 and 12) led to identifying anomalies associated with actual events that have not been evident before the filtering of irrelevant tweets.

We used McNemar’s test^54^ to assess the relative difference in the proportion of error between two models, i.e. before and after filtering irrelevant tweets. Given the actual list of outbreaks, the prediction results were transformed into a contingency table shown in Table 8. The first cell in the table is the total number of anomalies that were correctly identified by both original and relevant time-series. The second cell shows that there was no event that the original time-series could help in decreasing the number of false positives and false negatives in comparison to relevant time-series. The third cell shows that relevant time-series enabled us to have more correct detection than when we used original time-series. The last cell shows that there were 15 events that could not be identified using either of time-series. The table was then used in McNemar’s test, and the p-value of 0.062 was obtained. This could show that the accuracy difference between models was marginally significant.

**Table 8:** McNemar’s test - contingency table.

Original/Relevant	correct	incorrect
correct	40	0
incorrect	5	15

**Figure 13:**
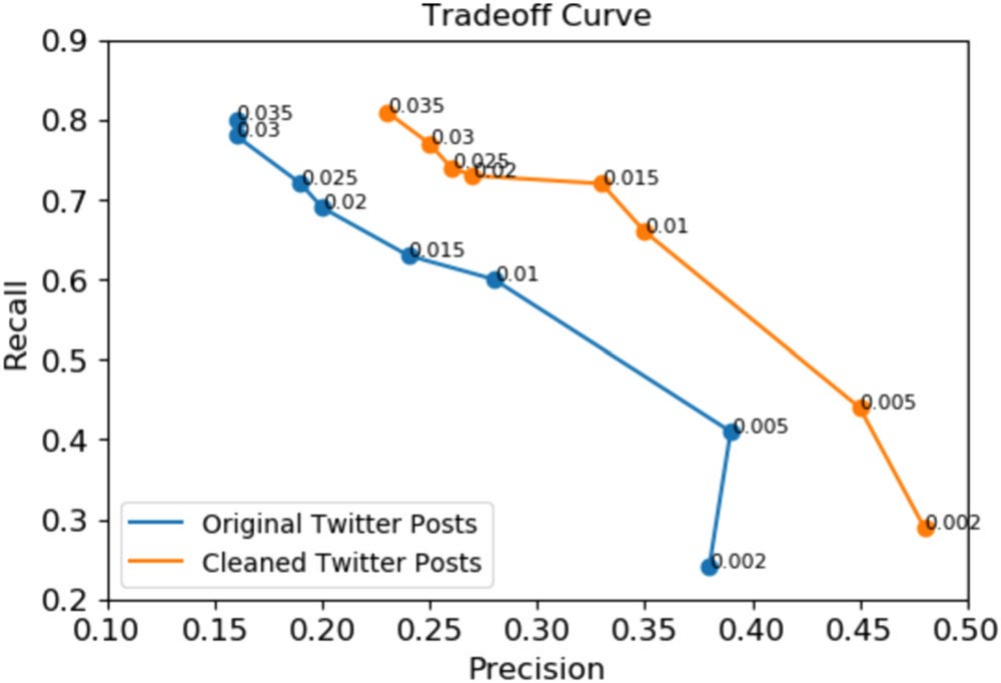
A comparison of precision-recall tradeoff curves: Overall performance of anomaly detection for original (blue) and relevant (orange) tweets.

Moreover, the plots in Fig. 13 for the original and cleaned signal of tweets were depicted in blue and orange colours, respectively. The plots characterized how well relevant tweets performed compared to original tweets in terms of obtaining all and only report dates. Looking at the plots, the precision of the anomaly detection for cleaned tweets was considerably higher than original tweets. On the other hand, the recall measure was slightly higher than or equal to original tweets. This could show that filtering irrelevant tweets can significantly help to decrease the number of false alarms in the monitoring of AI from Twitter.

### **Twitter for early warning of AI outbreaks**

Overall, we discussed 58 immediate outbreak notifications reported regarding 21 countries. We found 18 immediate outbreaks being reported on Twitter earlier than the official OIE. That being said, approximately one-third of the outbreaks led to a surge in the number of discussions on Twitter earlier than the announcement of official reports. For example, spikes in Twitter discussions in late September 2018, late June 2018 and late October 2017 related to Bulgaria, Japan and France, respectively, occurred before immediate reports. Our observation showed that the period between the spikes and immediate report dates varies between 1–15 days. Overall, this shows the potential of Twitter to be incorporated into AI surveillance systems for prediction purposes.

## Conclusion and Future Work

In the present study, an Internet-based tracking system was developed to monitor AI events from Twitter. We investigated the extent to which Twitter can serve as a complementary or additional information source of AI outbreak reports. The results confirmed that the system introduced here can be used to complement traditional surveillance systems. More precisely, this system extracts information similar to traditional systems in terms of time, magnitude and virus subtype of outbreaks. Here, however, we did not identify pathogenicity of viruses from Twitter which could be studied in future studies to help understand the severity of outbreaks better.

The system can also, to some extent, enhance the capacity of official systems by providing early warning of outbreaks. This can potentially assist animal health authorities in preventing or mitigating the impact of possible AI events. Furthermore, it contributes to the literature by improving the performance of AI event-detection procedure with the help of a semi-supervised sentiment-learning model. The basic principles of our work could be applied to other types of infectious diseases of animals to improve digital disease surveillance by filtering irrelevant tweets to reduce background noise. In future, adding non-contextual information of tweets such as the number of ‘re-tweets’ and ‘likes’ to the analysis might lead to more precise results. Also, future research can extend this study by using regional-based and multi-language data extractions and analyses.

## Supplementary information

Supplementary information

## Data Availability

The datasets generated and analysed during the current study are available from the corresponding author on reasonable request.

## References

[1] WHO. *World Health Organization*, Available on http://www.who.int/ (Accessed December 2018).

[2] Perrotta, D., Tizzoni, M. & Paolotti, D. Using participatory Web-based surveillance data to improve seasonal influenza forecasting in Italy. In *Proceedings of the 26th International Conference on World Wide Web*, 303–310 (Perth, Australia, 2017).

[3] Krieck, M., Dreesman, J., Otrusina, L. & Denecke, K. A new age of public health: Identifying disease outbreaks by analyzing tweets. In *Proceedings of Health Web-Science Workshop*, *ACM Web Science Conference* (New York, USA, 2011).

[4] Stewart, A. *et al*. Why is it difficult to detect sudden and unexpected epidemic outbreaks in twitter? *CoRR* abs/1611.03426 (2016).

[5] Allen C, Tsou M-H, Aslam A, Nagel A, Gawron J-M (2016). Applying GIS and machine learning methods to Twitter data for multiscale surveillance of influenza. PloS One.

[6] Ji X, Chun SA, Wei Z, Geller J (2015). Twitter sentiment classification for measuring public health concerns. Social Network Analysis and Mining.

[7] Yaesoubi, R. & Cohen, T. Adaptive decision-making during epidemics. In Zhang, N. K. S. (ed.) *Decision Analytics and Optimization in Disease Prevention and Treatment*, chap. 3, 59–79 (Wiley, 2018).

[8] Christaki E (2015). New technologies in predicting, preventing and controlling emerging infectious diseases. Virulence.

[9] Paul, M. J., Dredze, M. & Broniatowski, D. Twitter improves influenza forecasting. *PLoS Currents* **6**, ecurrents.outbreaks.90b9ed0f59bae4ccaa683a39865d9117 (2014). 10.1371/currents.outbreaks.90b9ed0f59bae4ccaa683a39865d9117 PMC4234396 25642377

[10] Broniatowski DA, Dredze M, Paul MJ, Dugas A (2015). Using social media to perform local influenza surveillance in an inner-city hospital: a retrospective observational study. JMIR Public Health and Surveillance.

[11] Santillana M (2015). Combining search, social media, and traditional data sources to improve influenza surveillance. PLoS Computational Biology.

[12] McGough SF, Brownstein JS, Hawkins JB, Santillana M (2017). Forecasting Zika incidence in the 2016 Latin America outbreak combining traditional disease surveillance with search, social media, and news report data. PLoS Neglected Tropical Diseases.

[13] van de Belt, T. H. *et al*. Social media posts and online search behaviour as early-warning system for MRSA outbreaks. *Antimicrobial Resistance and Infection Control* **7** (2018). 10.1186/s13756-018-0359-4 PMC5977481 29876100

[14] Schwind JS (2017). Online surveillance of media health event reporting in Nepal: digital disease detection from a One Health perspective. BMC International Health and Human Rights.

[15] Di Martino S (2017). Towards exploiting social networks for detecting epidemic outbreaks. Global Journal of Flexible Systems Management.

[16] Fast SM (2018). Predicting social response to infectious disease outbreaks from internet-based news streams. Annals of Operations Research.

[17] Culotta, A. Towards detecting influenza epidemics by analyzing twitter messages. In *Proceedings of the first workshop on social media analytics*, 115–122 (New York, NY, USA, 2010).

[18] Ahmed, W., Bath, P., Sbaffi, L. & Demartini, G. Using Twitter for insights into the 2009 swine flu and 2014 Ebola outbreaks. In *Proceedings of Lecture Notes in Computer Science*, *iConference*, 25–28 (Sheffield, UK, 2018).

[19] Atefeh F, Khreich W (2015). A survey of techniques for event detection in twitter. Computational Intelligence.

[20] Kunneman, F. & van den Bosch, A. Event detection in twitter: A machine-learning approach based on term pivoting. In *Proceedings of the 26th Benelux Conference on Artificial Intelligence*, 65–72 (Nijmegen, the Netherlands, 2014).

[21] Szomszor, M., Kostkova, P. & St Louis, C. Twitter informatics: tracking and understanding public reaction during the 2009 swine flu pandemic. In *Proceedings of 2011 IEEE/WIC/ACM International Conference on Web Intelligence and Intelligent Agent Technology (WI-IAT)*, 320–323 (Lyon, France, 2011).

[22] Shah, M. Disease propagation in social networks: a novel study of infection genesis and spread on twitter. In *Proceedings of Workshop on Big Data*, *Streams and Heterogeneous Source Mining: Algorithms*, *Systems*, *Programming Models and Applications*, 85–102 (San Francisco, CA, USA, 2016).

[23] Perveen N, Missen MMS, Rasool Q, Akhtar N (2016). Sentiment based twitter spam detection. International Journal of Advanced Computer Science and Applications (IJACSA).

[24] Byrd, K., Mansurov, A. & Baysal, O. Mining Twitter data for influenza detection and surveillance. In *Proceedings of the International Workshop on Software Engineering in Healthcare Systems*, 43–49 (Austen, Texas, 2016).

[25] Robertson C, Yee L (2016). Avian influenza risk surveillance in North America with online media. PloS One.

[26] Astill, J., Dara, R., Fraser, E., Sharif, S. Detecting and Predicting Emerging Disease in Poultry With the Implementation of New Technologies and Big Data: A Focus on Avian Influenza Virus. *Frontiers in Veterinary Science* **5**, 263 (2018). 10.3389/fvets.2018.00263 PMC6218608 30425995

[27] Corley CD, Cook DJ, Mikler AR, Singh KP (2010). Text and structural data mining of influenza mentions in web and social media. International Journal of Environmental Research and Public Health.

[28] Signorini A, Segre AM, Polgreen PM (2011). The use of twitter to track levels of disease activity and public concern in the U.S. during the influenza A H1N1 pandemic. PloS One.

[29] Romano, S. *Semantic-based knowledge management and document processing in the e-health domain*. Ph.D. thesis, Università degli Studi di Napoli Federico II (2013).

[30] Yousefi Naghani, S., Dara, R., Poljak, Z., Sharif, S. A review of knowledge discovery process in control and mitigation of avian influenza. *Animal Health Research Reviews, Cambridge University Press*, 1–11 (2019). 10.1017/S1466252319000033 31895021

[31] Lampos V, Cristianini N (2012). Nowcasting events from the social web with statistical learning. ACM Transactions on Intelligent Systems and Technology (TIST).

[32] Jain VK, Kumar S (2015). An effective approach to track levels of influenza-A (H1N1) pandemic in India using twitter. Procedia Computer Science.

[33] Woo H (2018). Identification of keywords from Twitter and web blog posts to detect influenza epidemics in Korea. Disaster Medicine and Public Health Preparedness.

[34] Achrekar, H., Gandhe, A., Lazarus, R., Yu, S.-H. & Liu, B. Predicting flu trends using Twitter data. In *Proceedings of 2011 IEEE Conference on Computer Communications Workshops (INFOCOM WKSHPS)*, 702–707 (Shanghai, P.R. China, 2011).

[35] Lampos, V. & Cristianini, N. Tracking the flu pandemic by monitoring the social web. In *Proceedings of 2010 2nd International Workshop on Cognitive Information Processing (CIP)*, 411–416 (Elba, Italy, 2010).

[36] Broniatowski DA, Paul MJ, Dredze M (2013). National and local influenza surveillance through twitter: an analysis of the 2012–2013 influenza epidemic. PloS One.

[37] Sharpe, D., Hopkins, R., Cook, R. L. & Striley, C. W. Using a bayesian method to assess Google, Twitter, and Wikipedia for ILI surveillance. *Online Journal of Public Health Informatics* **9** (2017).

[38] Search API. *Standard search API*, Available on https://developer.twitter.com/en/docs/tweets/search/api-reference/get-search-tweets.html (Accessed December 2018).

[39] Carmen. *Carmen documentation*, Available on https://carmen.readthedocs.io/en/latest/index.html (Accessed November 2017).

[40] Dredze, M., Paul, M. J., Bergsma, S. & Tran, H. Carmen: a Twitter geolocation system with applications to public health. In *Proceedings of AAAI Workshop on Expanding the Boundaries of Health Informatics Using AI (HIAI)*, 20–24 (Bellevue, Wa, 2013).

[41] OIE. *World Organization For Animal Health*, Available on https://www.oie.int/wahis_2/public/wahid.php/Diseaseinformation/WI (Accessed November 2017).

[42] Hasan M, Orgun MA, Schwitter R (2017). A survey on real-time event detection from the twitter data stream. Journal of Information Science.

[43] scikit learn. *scikit-learn software*, Available on https://scikit-learn.org (Accessed April 2019).

[44] Effrosynidis, D., Peikos, G., Symeonidis, S. & Arampatzis, A. Emoji prediction in tweets. In *Proceedings of The 12th International Workshop on Semantic Evaluation*, 466–469 (Louisiana, USA, 2018).

[45] McCallum, A. *et al*. A comparison of event models for naive bayes text classification. In *Proceedings of AAAI-98 Workshop on Learning for Text Categorization*, 41–48 (Madison, Wisconsin, USA, 1998).

[46] Leung, K. M. Naive bayesian classifier. Tech. Rep., Polytechnic University Department of Computer Science/Finance and Risk Engineering (2007).

[47] Nigam, K., McCallum, A. & Mitchell, T. Semi-supervised text classification using EM. In *Semi-Supervised Learning*, 33–56 (Massachusetts, USA, 2006).

[48] Vieira, R. G., Leone Filho, M. A. & Semolini, R. An Enhanced Seasonal-Hybrid ESD technique for robust anomaly detection on time series. In *Simpósio Brasileiro de Redes de Computadores (SBRC)*, vol. 36 (São Paulo, Brazil, 2018).

[49] Laptev, N., Amizadeh, S. & Flint, I. Generic and scalable framework for automated time-series anomaly detection. In *Proceedings of the 21th ACM SIGKDD International Conference on Knowledge Discovery and Data Mining*, 1939–1947 (ACM, Sydney, NSW, Australia, 2015).

[50] Hochenbaum, J., Vallis, O. S. & Kejariwal, A. Automatic anomaly detection in the cloud via statistical learning. *CoRR* abs/1704.07706 (2017).

[51] Ahmad, S. & Purdy, S. Real-time anomaly detection for streaming analytics. *CoRR* abs/1607.02480 (2016).

[52] AnomalyDetection. *AnomalyDetection R package*, Available on https://www.rdocumentation.org/packages/anomalyDetection/versions/0.1.2 (Accessed December 2018).

[53] Blei DM, Ng AY, Jordan MI (2003). Latent dirichlet allocation. Journal of Machine Learning Research.

[54] Alpaydin, E. *Introduction to machine learning* (MIT press, 2009).

