# Supplementary material for: The Assessment of Twitter’s Potential for Outbreak Detection: Avian Influenza Case Study
Source: Sci Rep. 2019 Dec 3;9:18147. doi: 10.1038/s41598-019-54388-4 (PMC6890696; doi:10.1038/s41598-019-54388-4)
Supplement: Supplementary file 1 — Supplementary information [file 41598_2019_54388_MOESM1_ESM.pdf]

The Assessment of Twitter's Potential for Outbreak Detection: Avian Influenza Case Study

Samira YousefiNaghani[1], **Rozita Dara**[1,*], **Zvonimir Poljak**[2], **Theresa M Bernardo**[2], **and Shayan Sharif**[3]

[1]School of Computer Science, University of Guelph, Guelph, Ontario, Canada
[2]Department of Population Medicine, Ontario Veterinary College, University of Guelph, Guelph, Ontario, Canada
[3]Department of Pathobiology, University of Guelph, Guelph, Ontario, Canada
[*]Requests for materials should be addressed to drozita@uoguelph.ca

ABSTRACT

Social media services such as Twitter are valuable sources of information for surveillance systems. A digital syndromic surveillance system has several advantages including its ability to overcome the problem of time delay in traditional surveillance systems. Despite the progress made with using digital syndromic surveillance systems, the possibility of tracking avian influenza (AI) using online sources has not been fully explored. In this study, a Twitter-based data analysis framework was developed to automatically monitor avian influenza outbreaks in a real-time manner. The framework was implemented to find worrisome posts and alerting news on Twitter, filter irrelevant ones, and detect the onset of outbreaks in several countries. The system collected and analyzed over 209,000 posts discussing avian influenza on Twitter from July 2017 to November 2018. We examined the potential of Twitter data to represent the date, severity and virus type of official reports. Furthermore, we investigated whether filtering irrelevant tweets can positively impact the performance of the system. The proposed approach was empirically evaluated using a real-world outbreak-reporting source. We found that 75% of real-world outbreak notifications of AI were identifiable from Twitter. This shows the capability of the system to serve as a complementary approach to official AI reporting methods. Moreover, we observed that one-third of outbreak notifications were reported on Twitter earlier than official reports. This feature could augment traditional surveillance systems and provide a possibility of early detection of outbreaks. This study could potentially provide a first stepping stone for building digital disease outbreak warning systems to assist epidemiologists and animal health professionals in making relevant decisions.

Figures

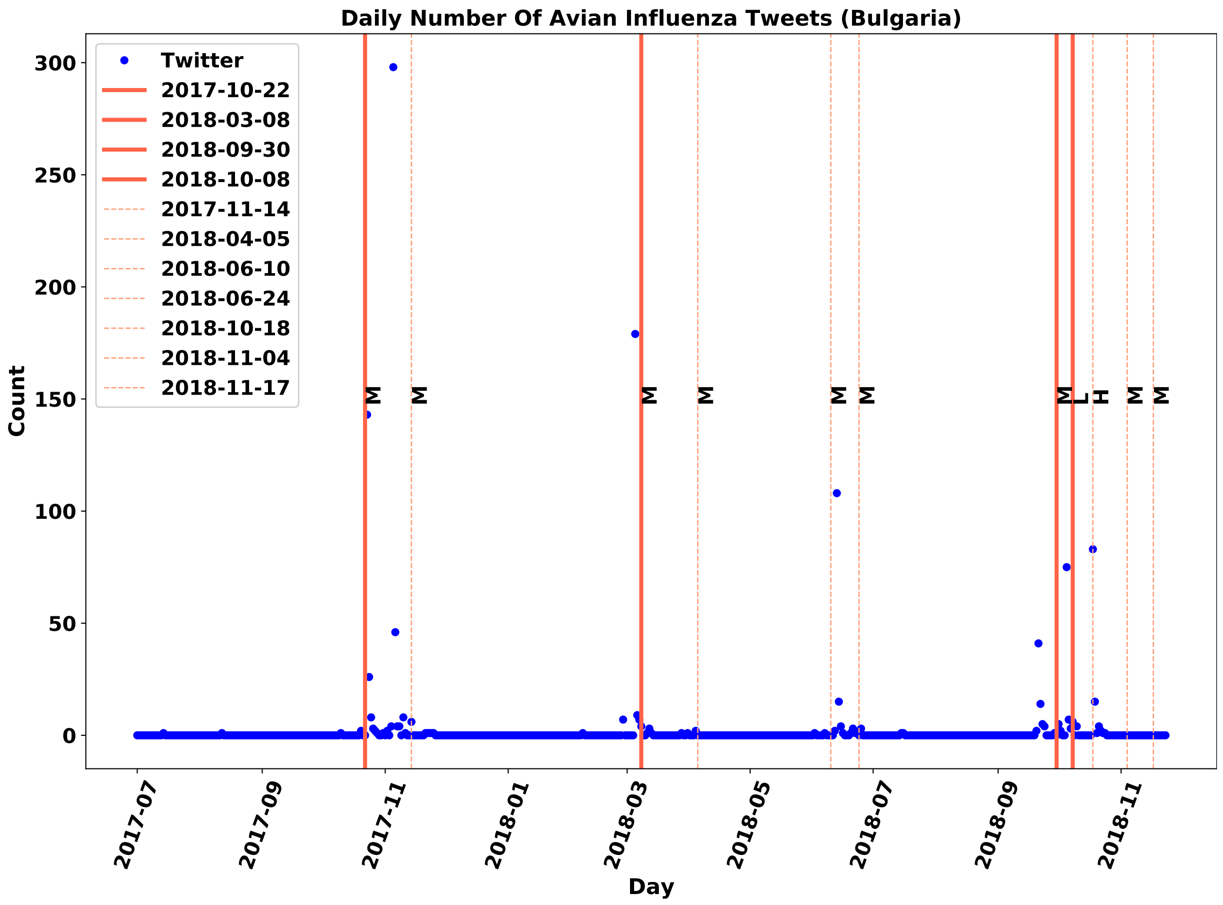

Figure 1. Twitter Daily Count Data (blue points), Immediate (red lines) and Follow-up (dash lines) Outbreak Notifications

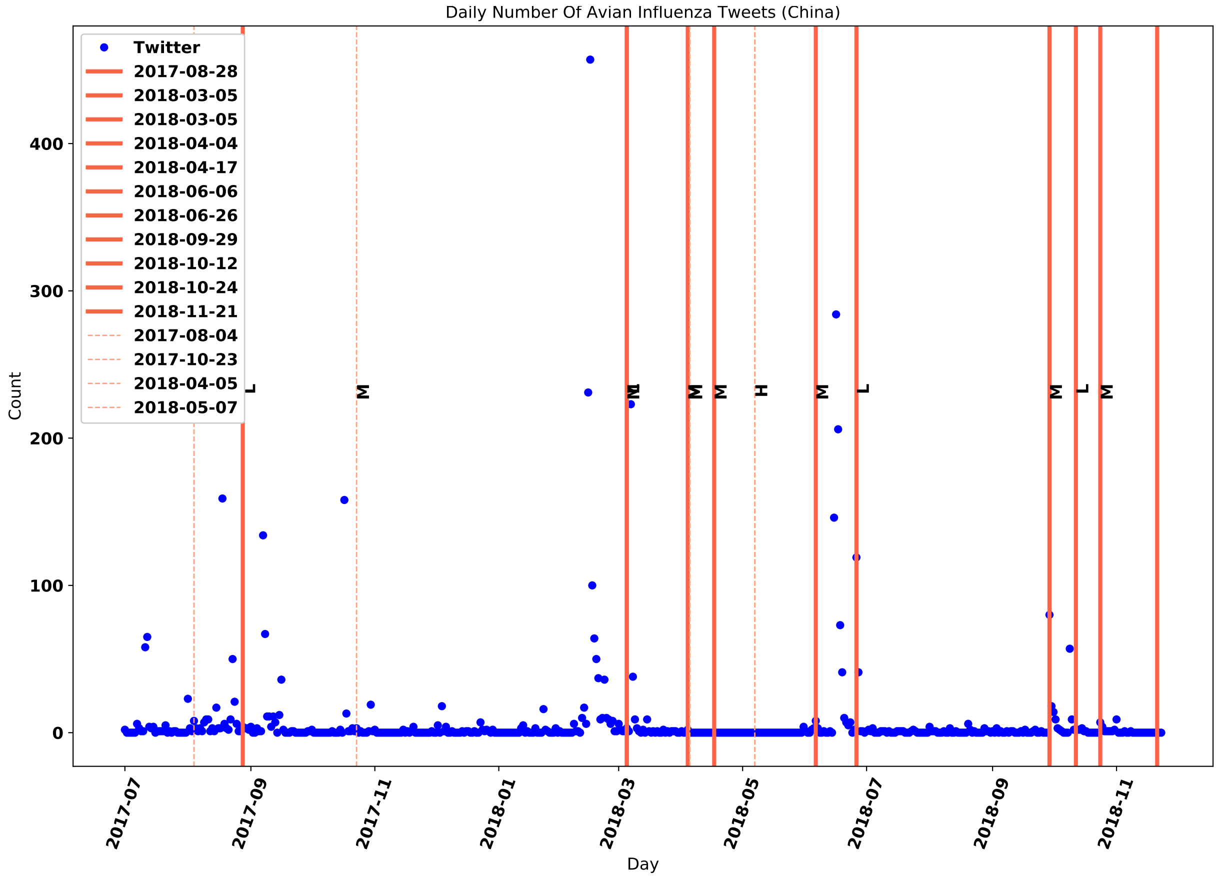

Figure 2. Twitter Daily Count Data (blue points), Immediate (red lines) and Follow-up (dash lines) Outbreak Notifications

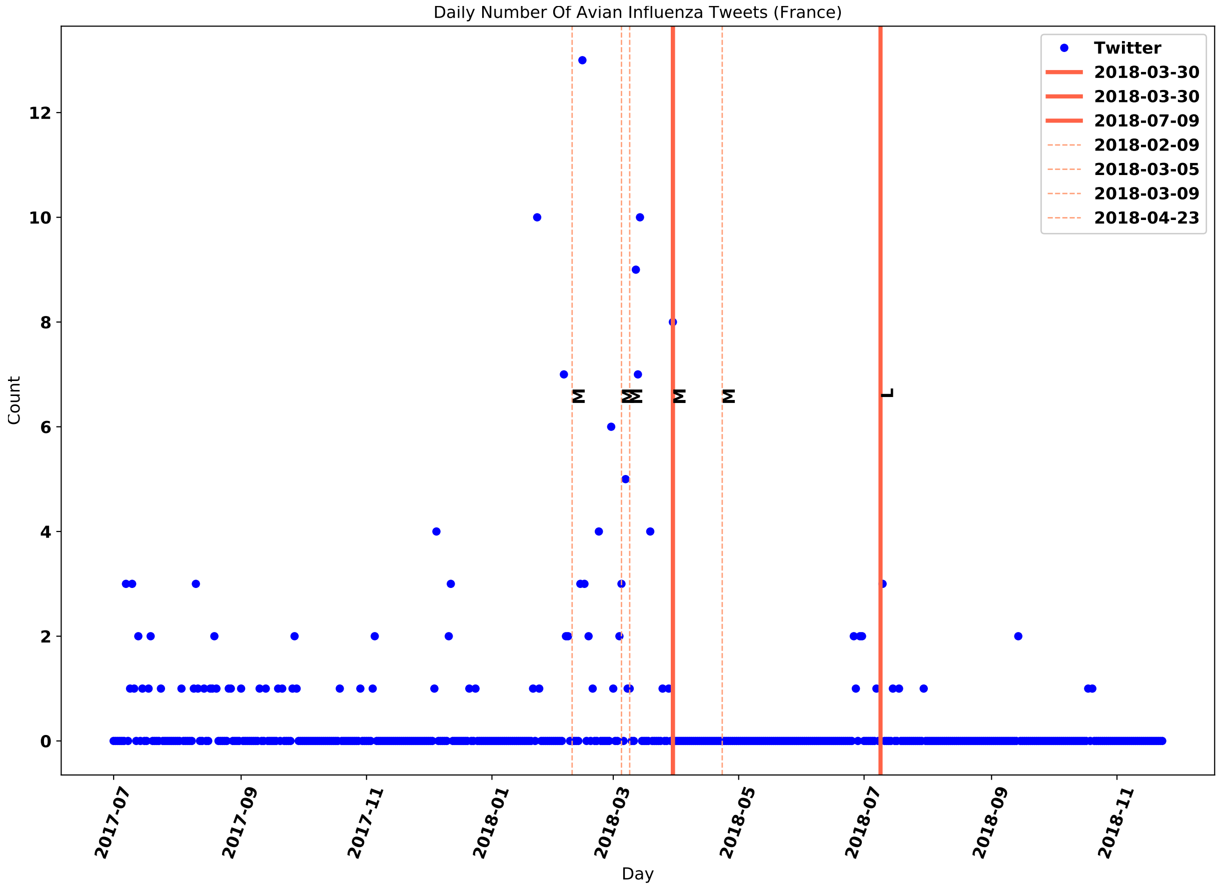

Figure 3. Twitter Daily Count Data (blue points), Immediate (red lines) and Follow-up (dash lines) Outbreak Notifications

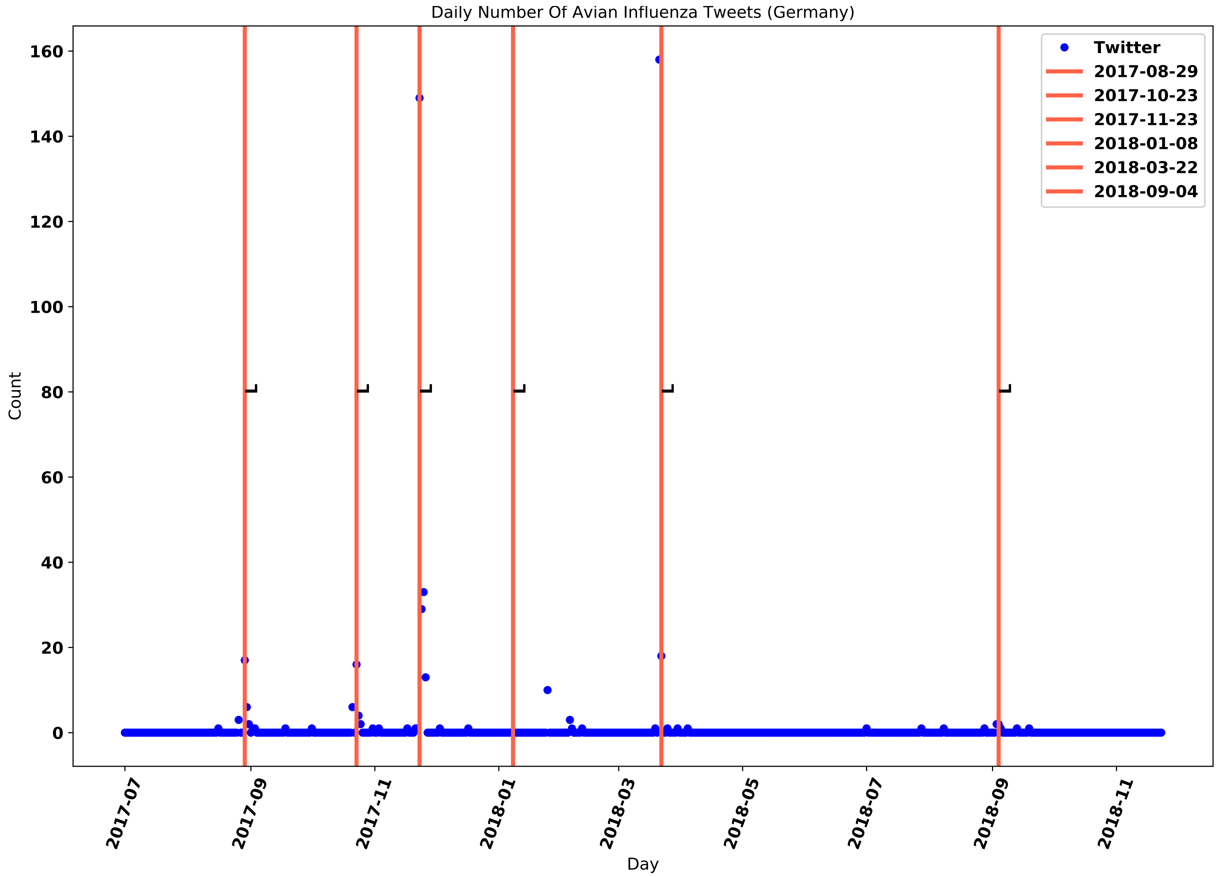

Figure 4. Twitter Daily Count Data (blue points), Immediate (red lines) and Follow-up (dash lines) Outbreak Notifications

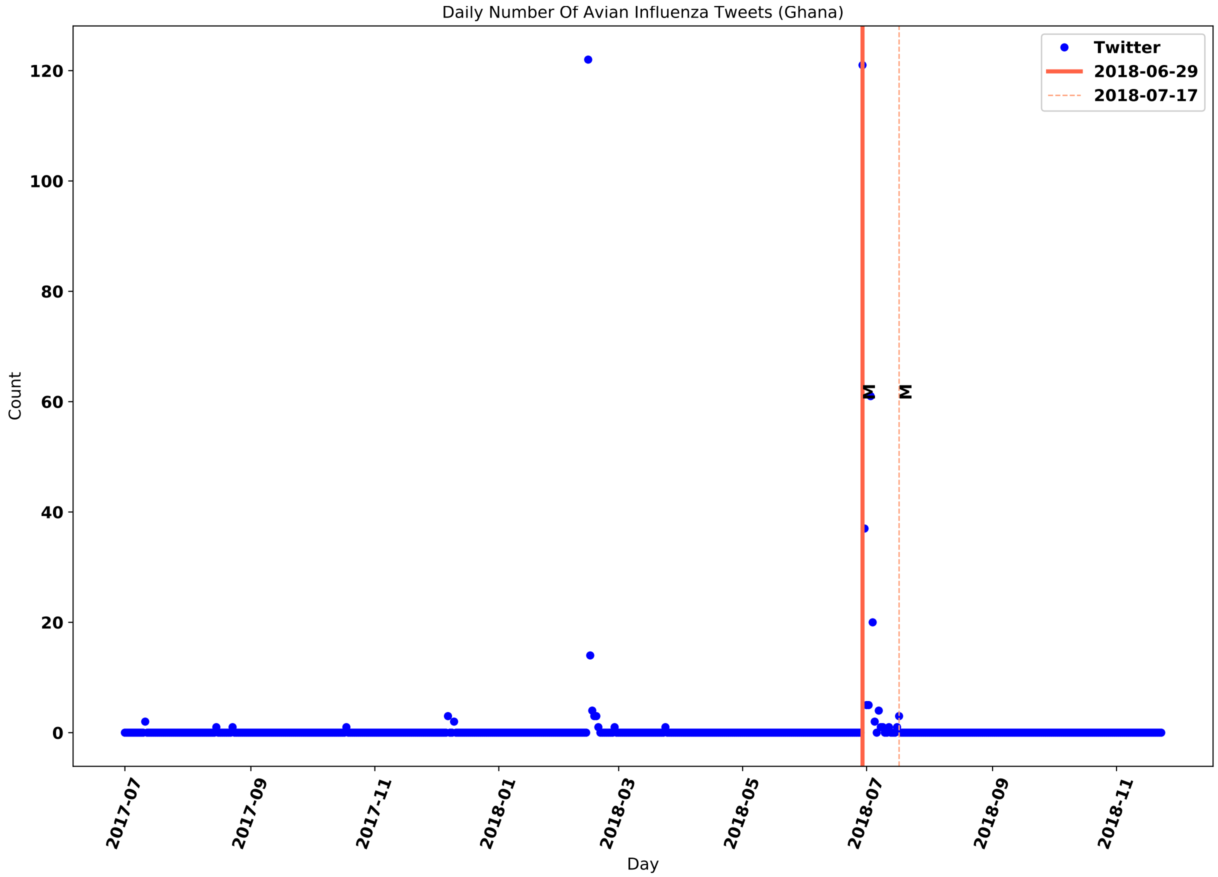

Figure 5. Twitter Daily Count Data (blue points), Immediate (red lines) and Follow-up (dash lines) Outbreak Notifications

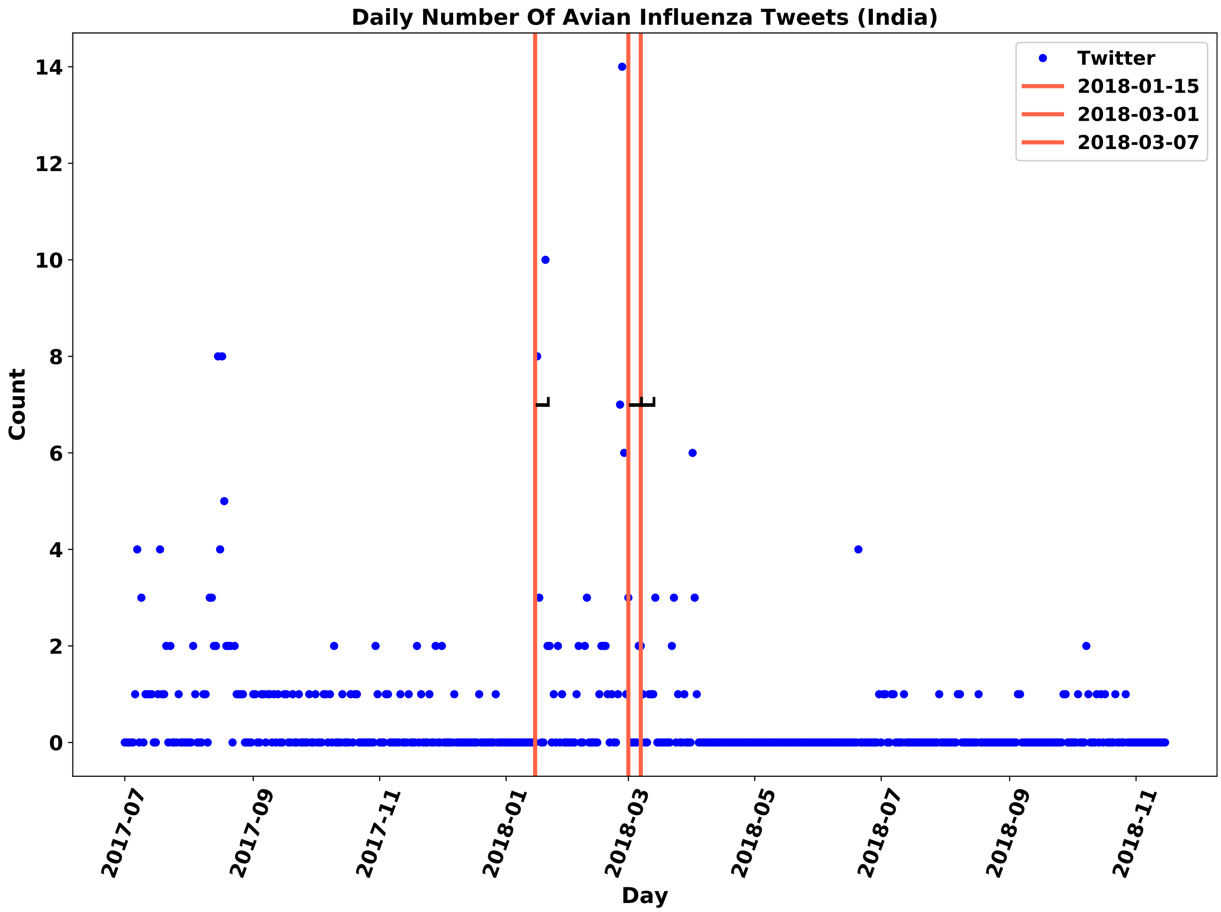

Figure 6. Twitter Daily Count Data (blue points), Immediate (red lines) and Follow-up (dash lines) Outbreak Notifications

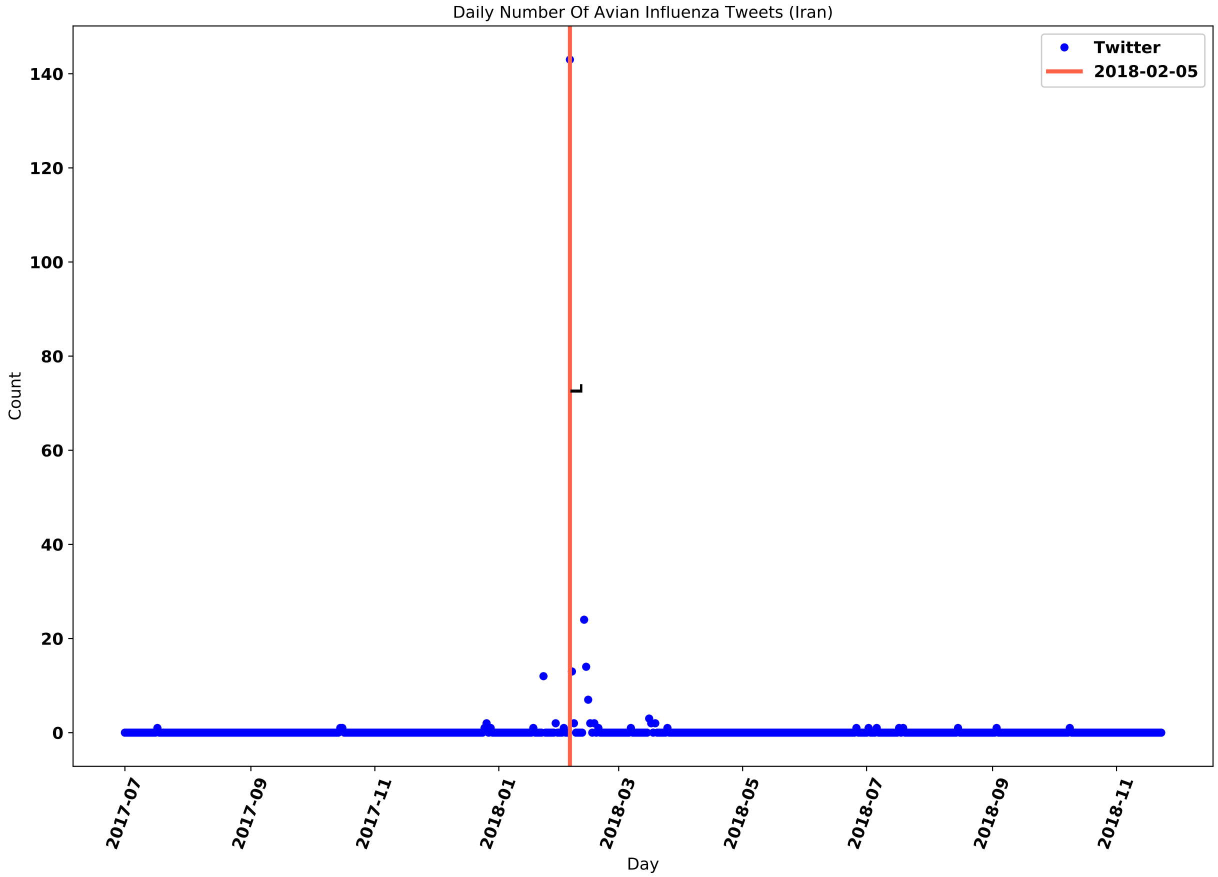

Figure 7. Twitter Daily Count Data (blue points), Immediate (red lines) and Follow-up (dash lines) Outbreak Notifications

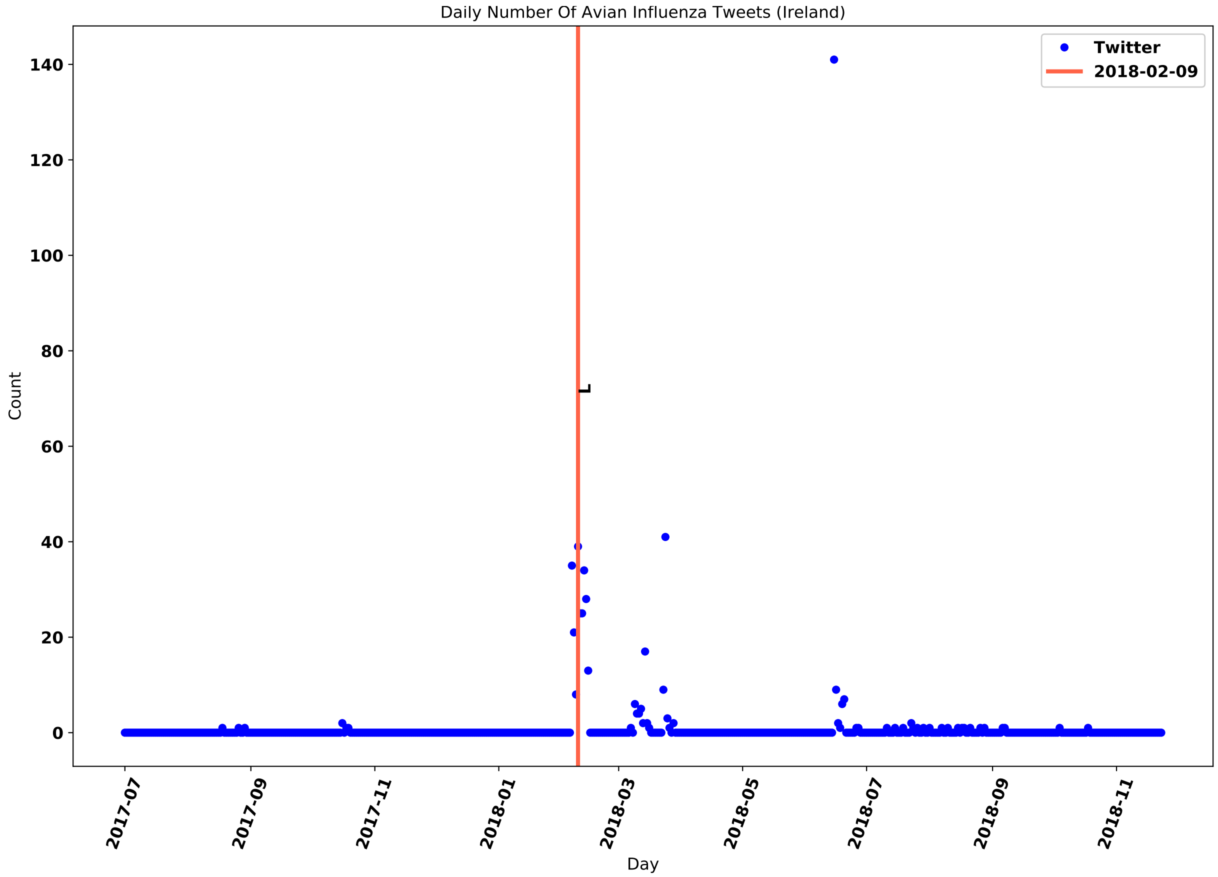

Figure 8. Twitter Daily Count Data (blue points), Immediate (red lines) and Follow-up (dash lines) Outbreak Notifications

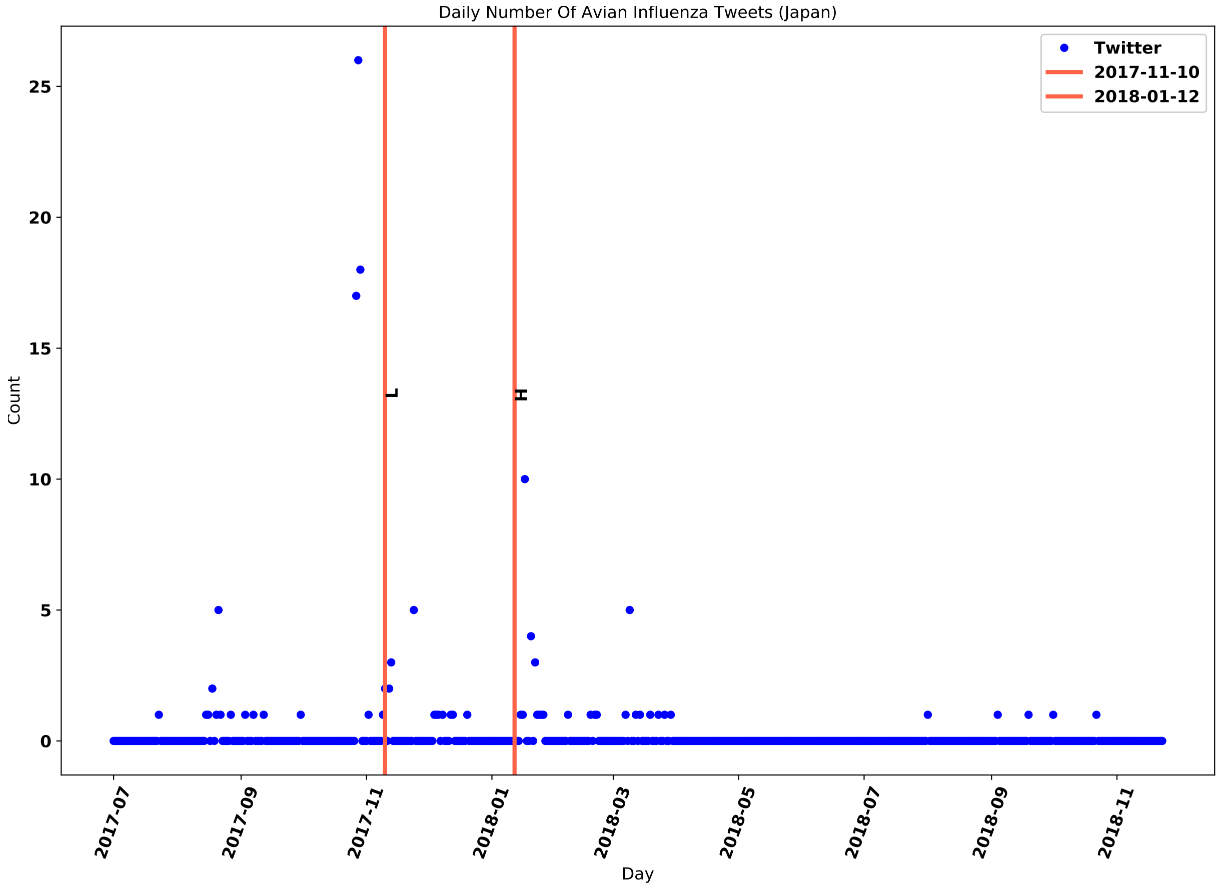

Figure 9. Twitter Daily Count Data (blue points), Immediate (red lines) and Follow-up (dash lines) Outbreak Notifications

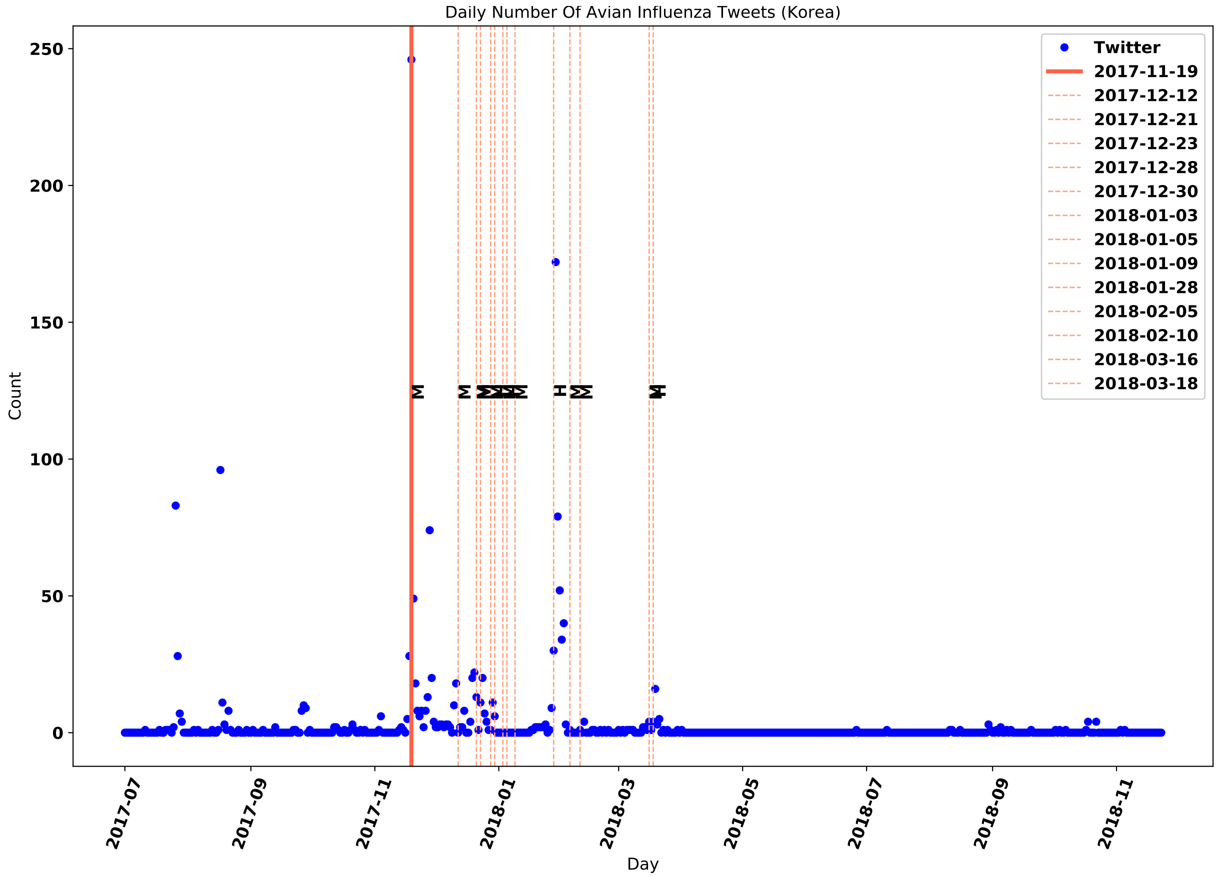

Figure 10. Twitter Daily Count Data (blue points), Immediate (red lines) and Follow-up (dash lines) Outbreak Notifications

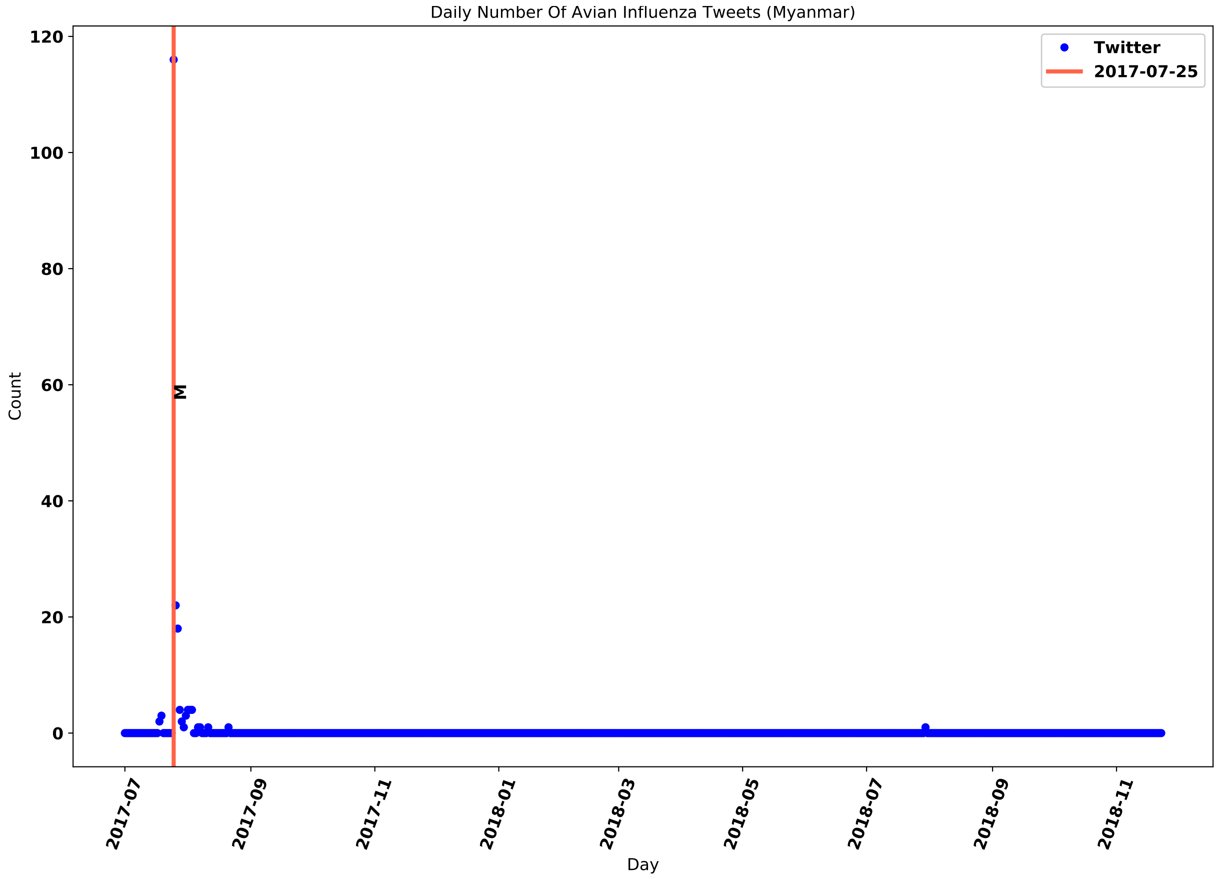

Figure 11. Twitter Daily Count Data (blue points), Immediate (red lines) and Follow-up (dash lines) Outbreak Notifications

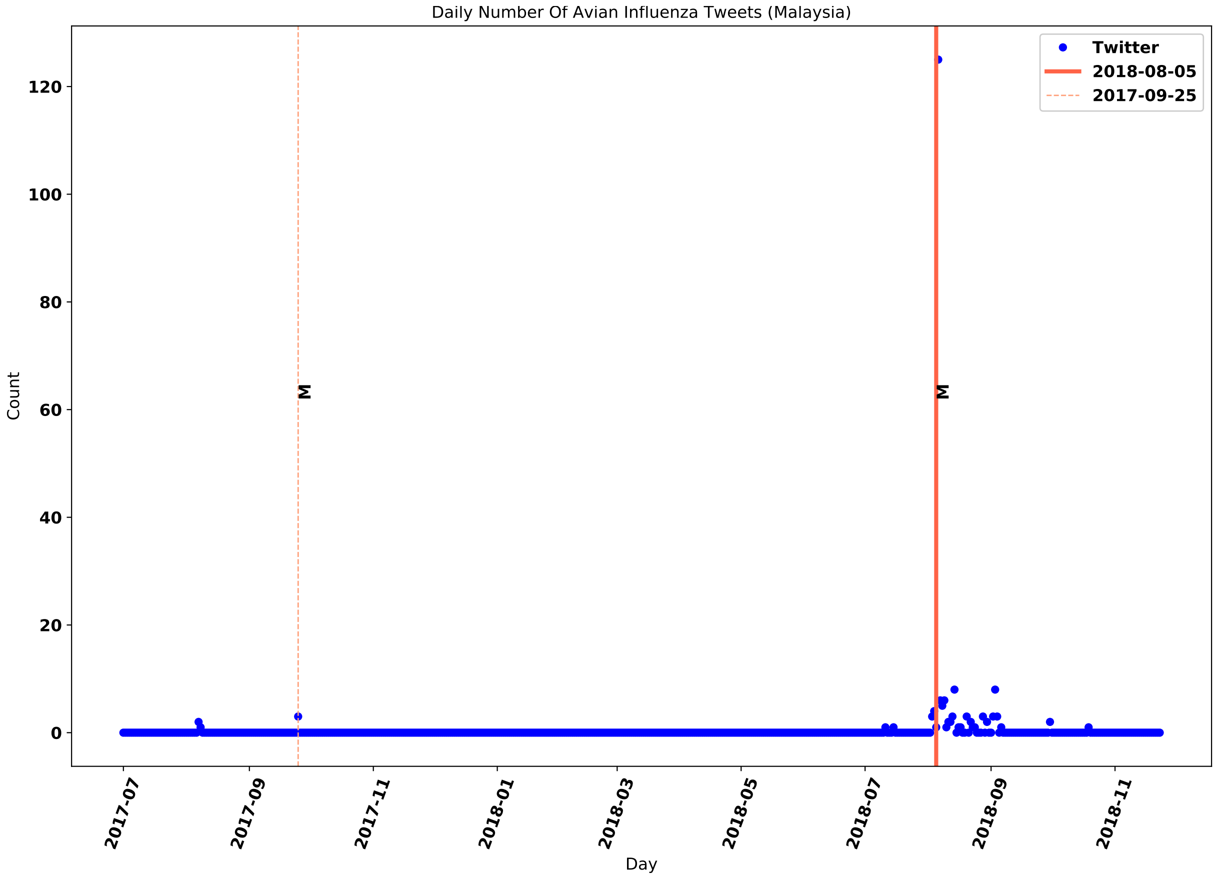

Figure 12. Twitter Daily Count Data (blue points), Immediate (red lines) and Follow-up (dash lines) Outbreak Notifications

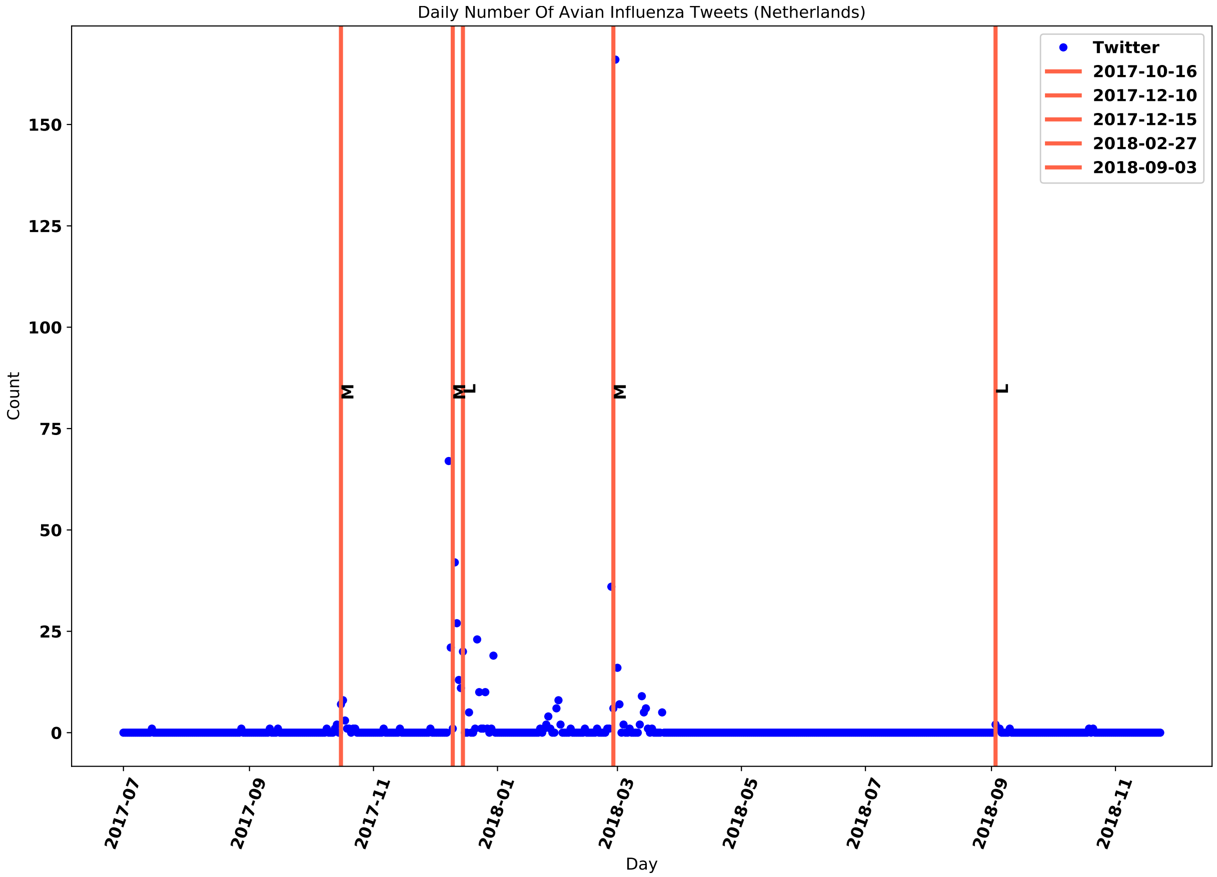

Figure 13. Twitter Daily Count Data (blue points), Immediate (red lines) and Follow-up (dash lines) Outbreak Notifications

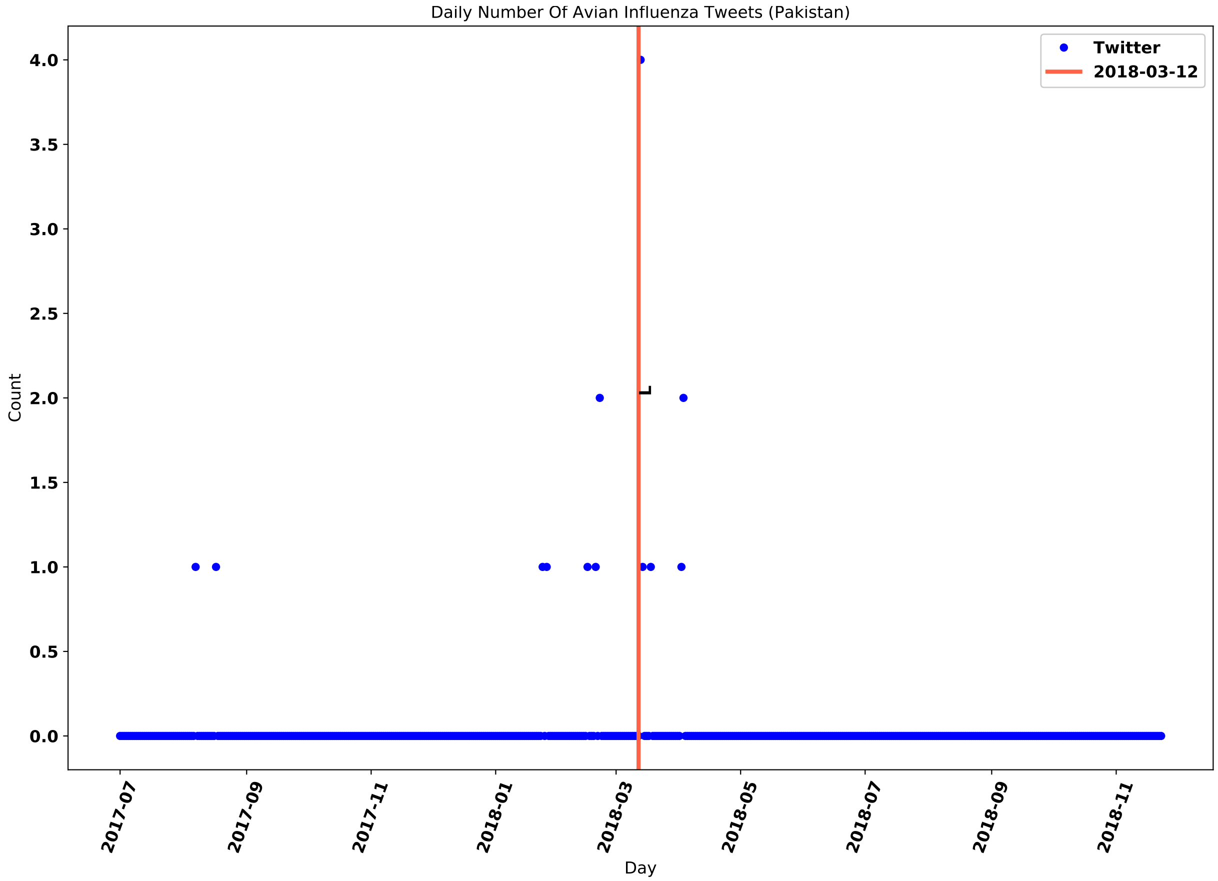

Figure 14. Twitter Daily Count Data (blue points), Immediate (red lines) and Follow-up (dash lines) Outbreak Notifications

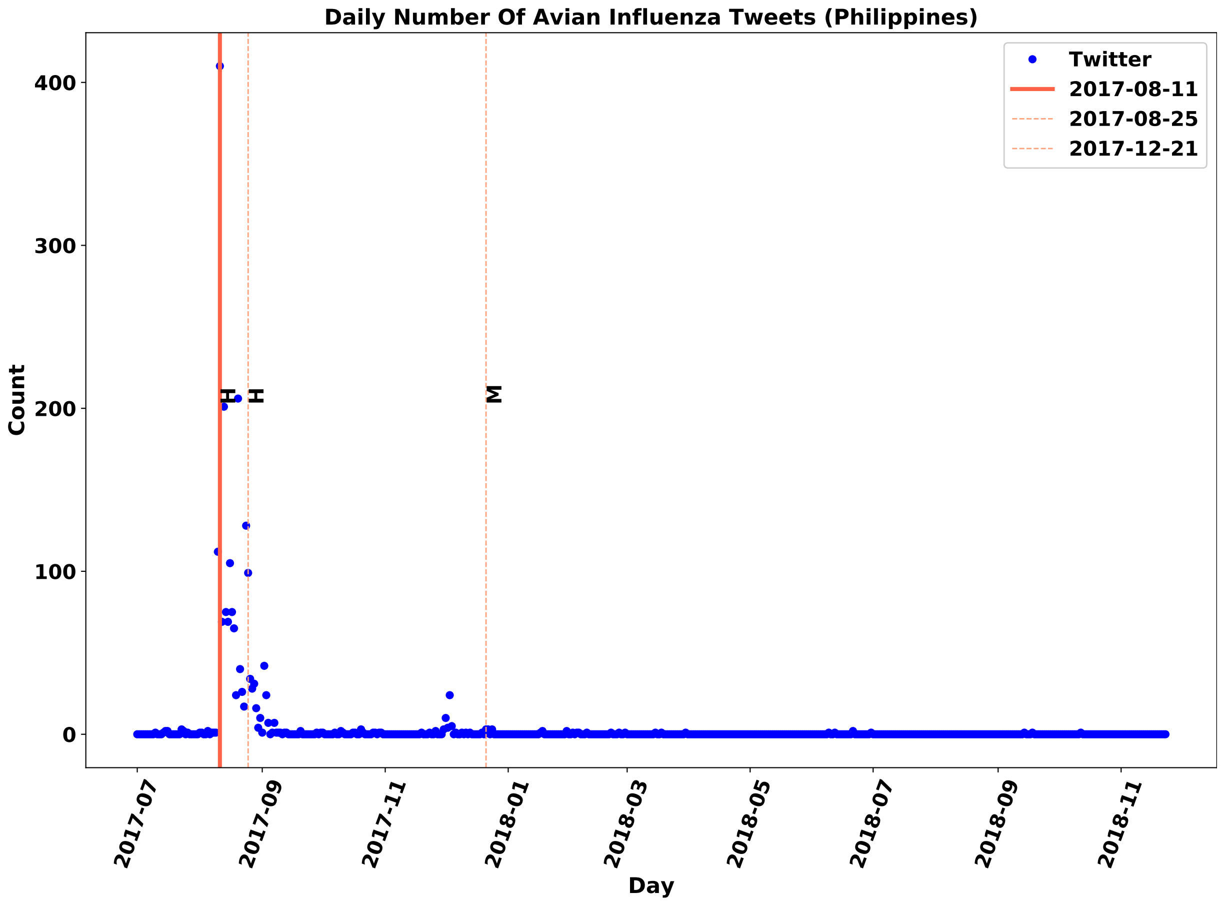

Figure 15. Twitter Daily Count Data (blue points), Immediate (red lines) and Follow-up (dash lines) Outbreak Notifications

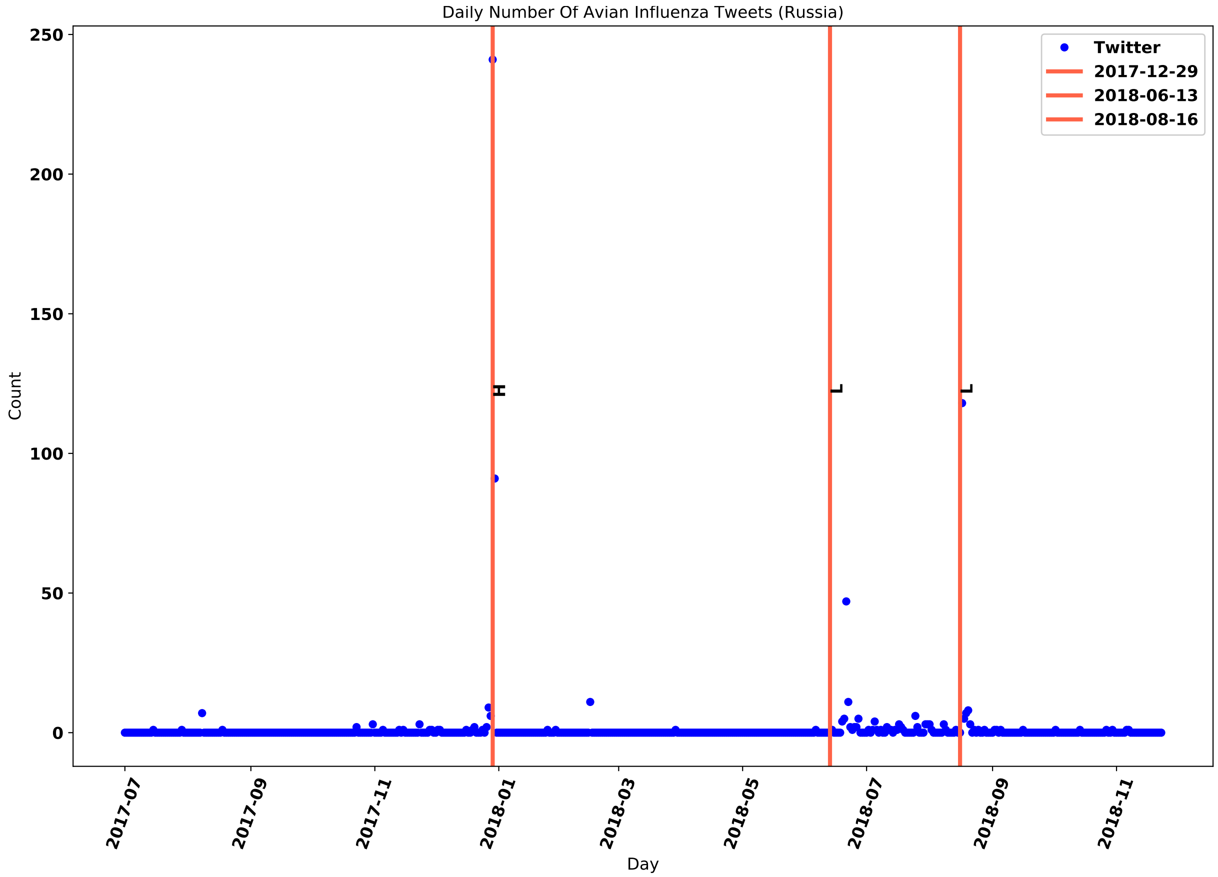

Figure 16. Twitter Daily Count Data (blue points), Immediate (red lines) and Follow-up (dash lines) Outbreak Notifications

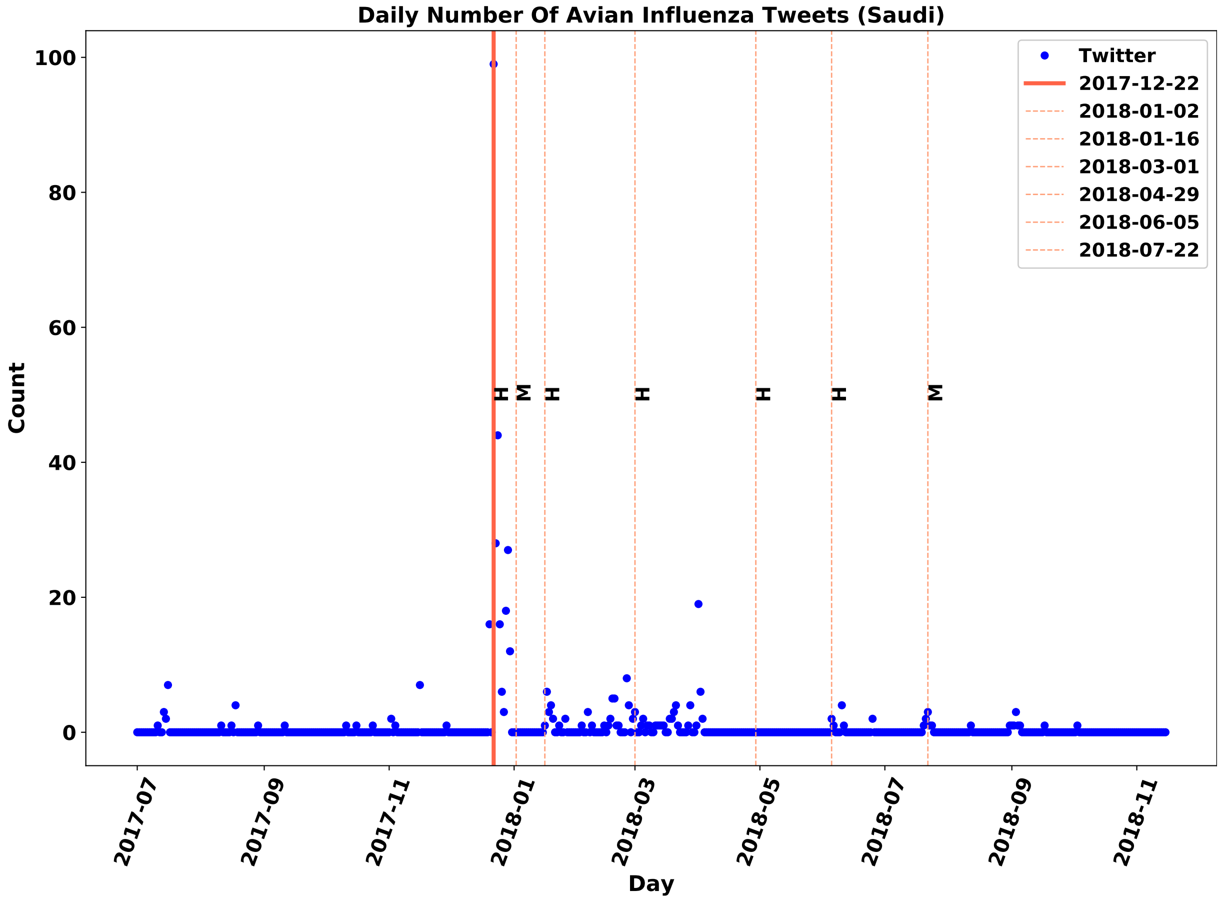

Figure 17. Twitter Daily Count Data (blue points), Immediate (red lines) and Follow-up (dash lines) Outbreak Notifications

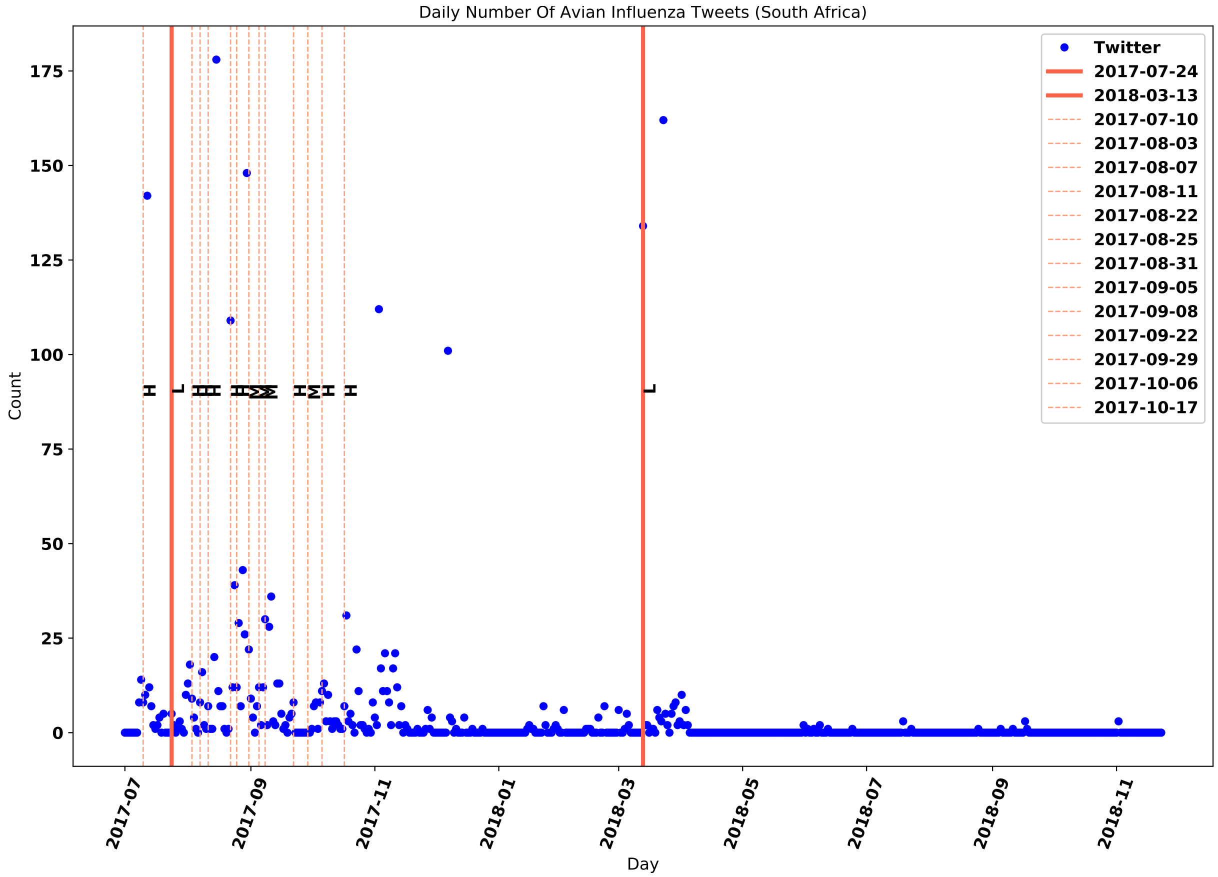

Figure 18. Twitter Daily Count Data (blue points), Immediate (red lines) and Follow-up (dash lines) Outbreak Notifications

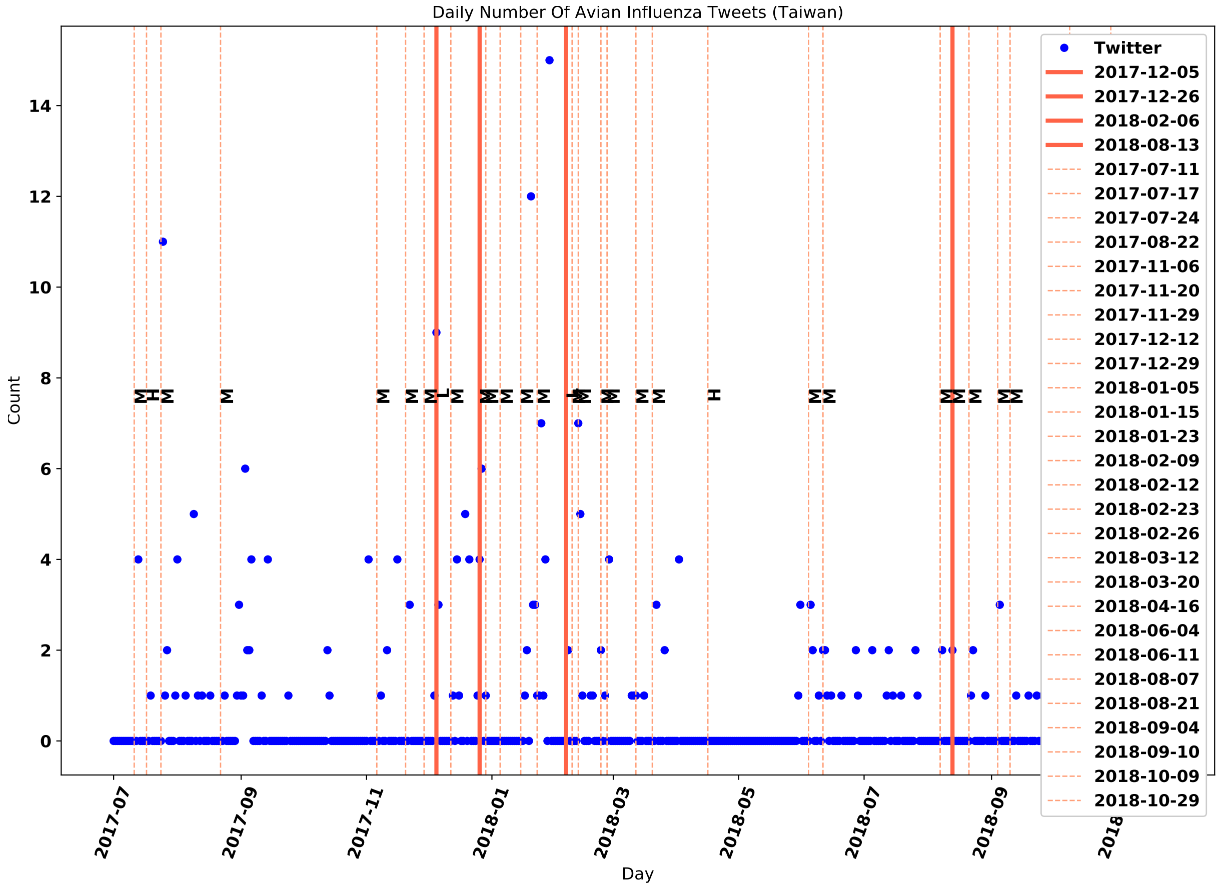

Figure 19. Twitter Daily Count Data (blue points), Immediate (red lines) and Follow-up (dash lines) Outbreak Notifications

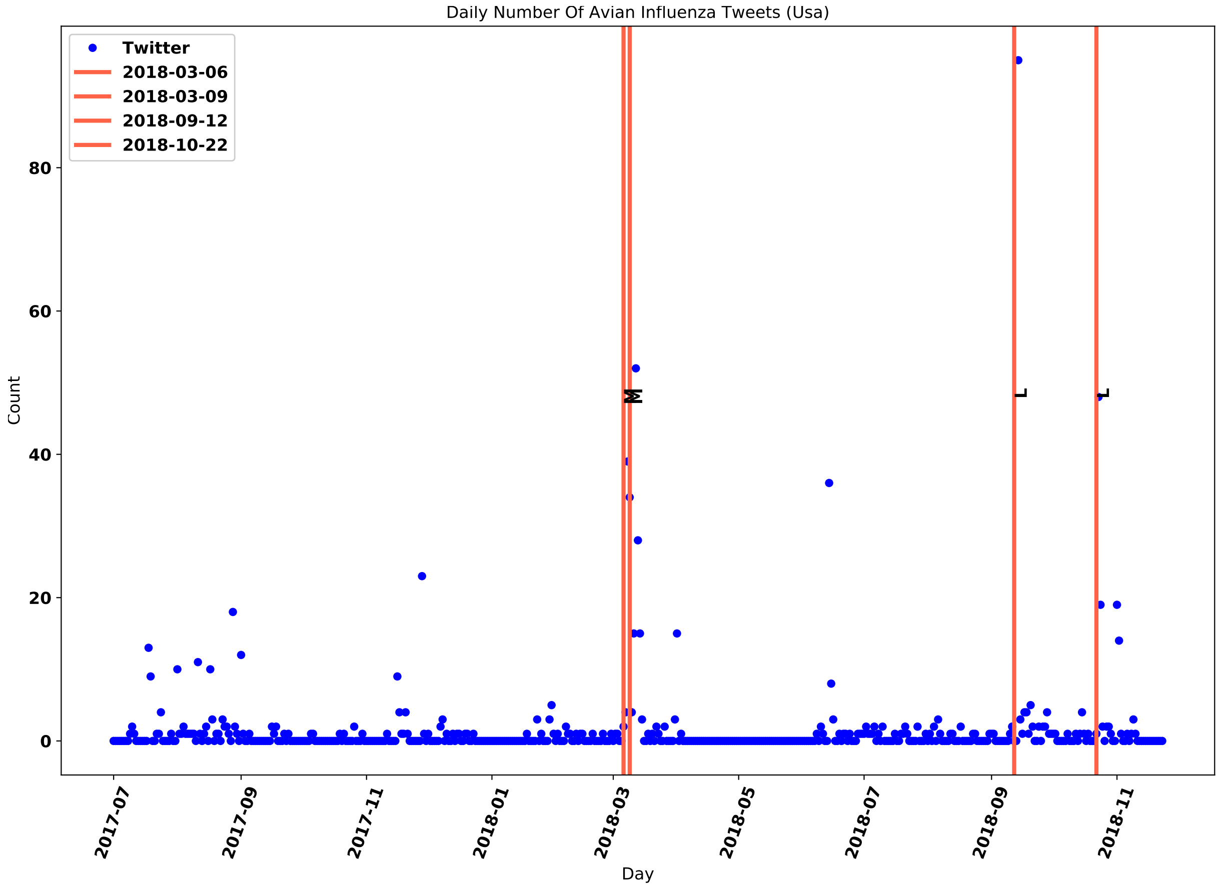

Figure 20. Twitter Daily Count Data (blue points), Immediate (red lines) and Follow-up (dash lines) Outbreak Notifications

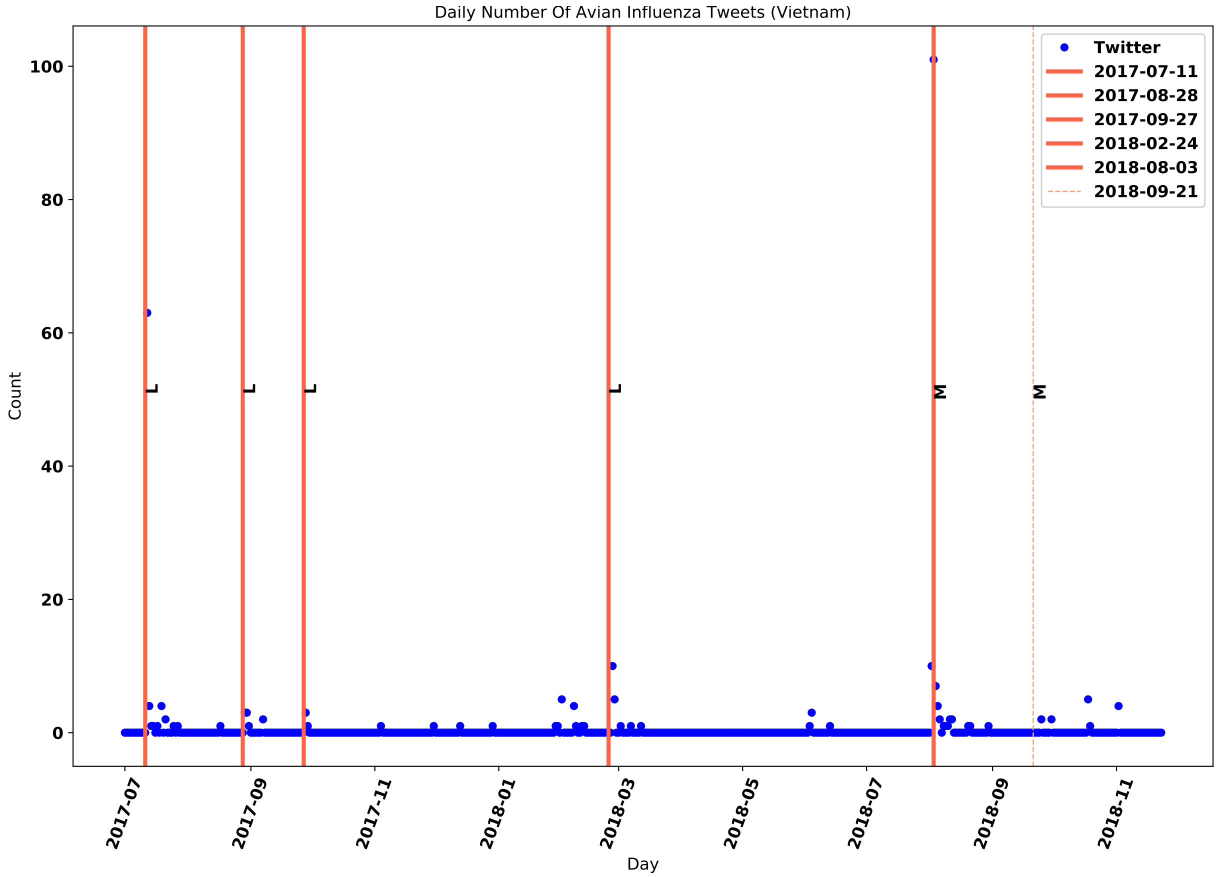

Figure 21. Twitter Daily Count Data (blue points), Immediate (red lines) and Follow-up (dash lines) Outbreak Notifications

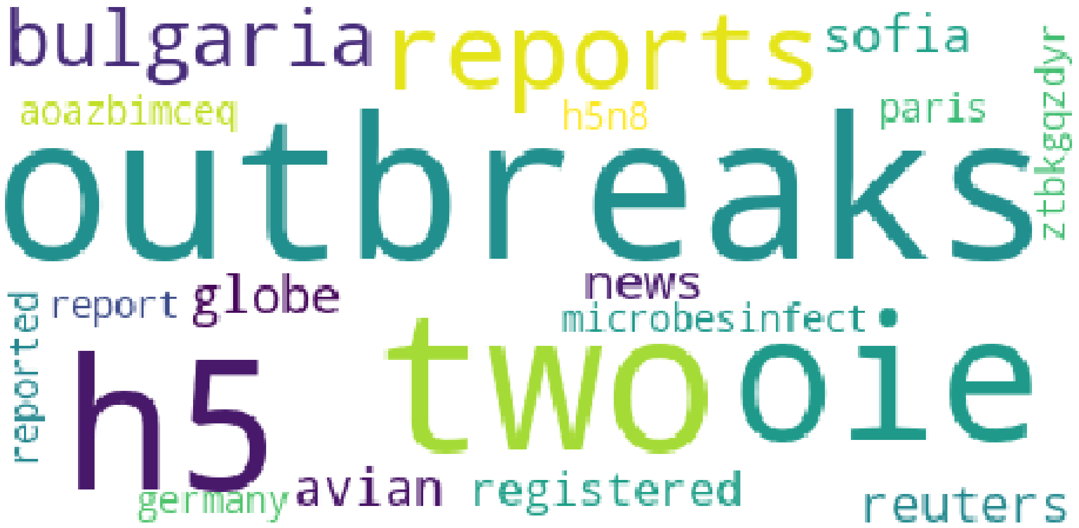

Figure 22. Word Clouds Around Outbreak Dates (Bulgaria)

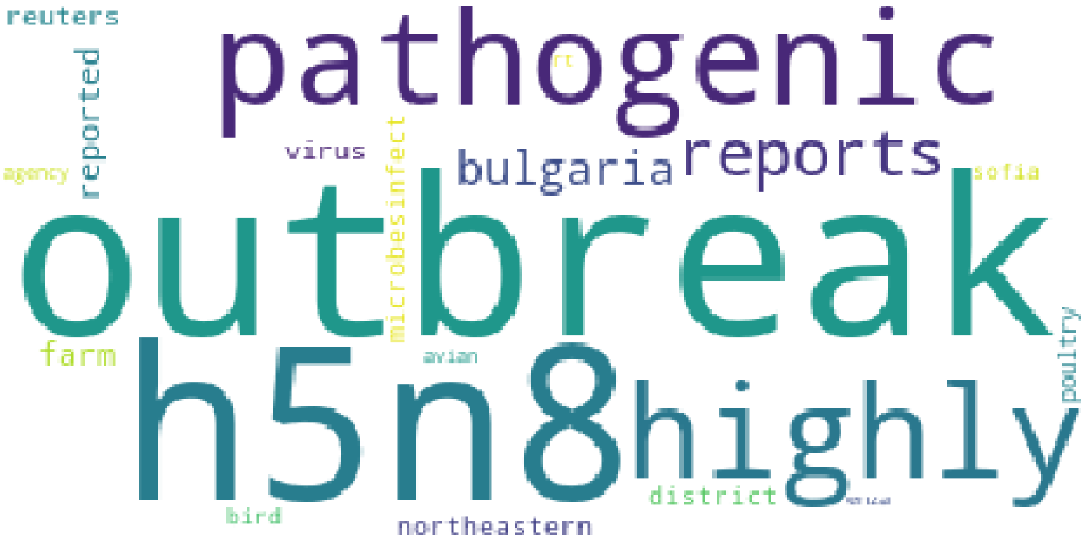

Figure 23. Word Clouds Around Outbreak Dates (Bulgaria)

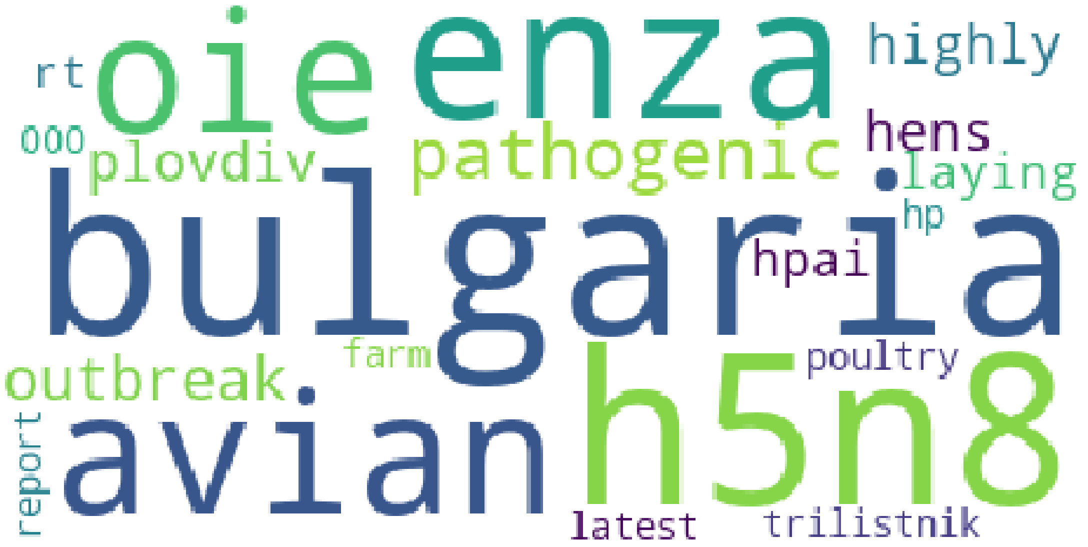

Figure 24. Word Clouds Around Outbreak Dates (Bulgaria)

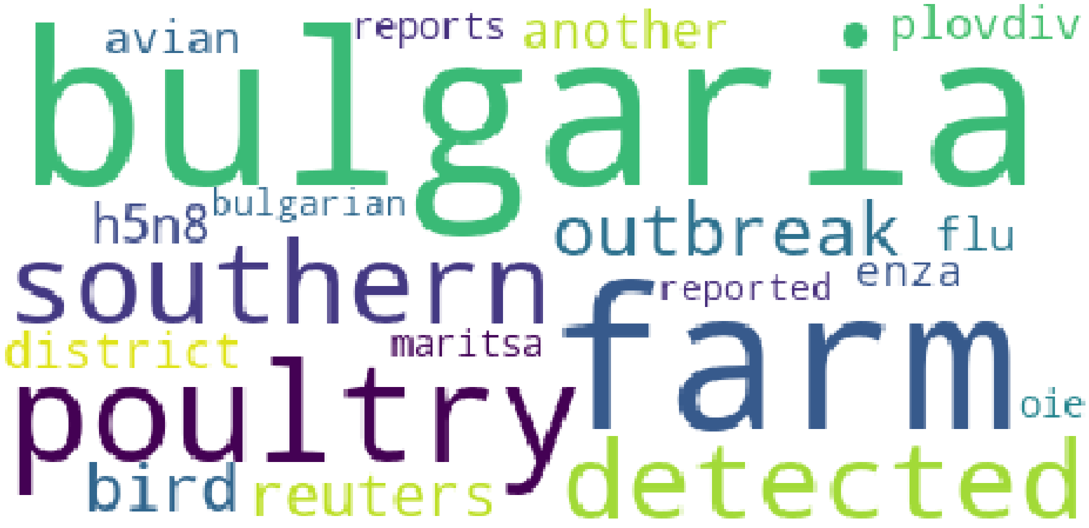

Figure 25. Word Clouds Around Outbreak Dates (Bulgaria)

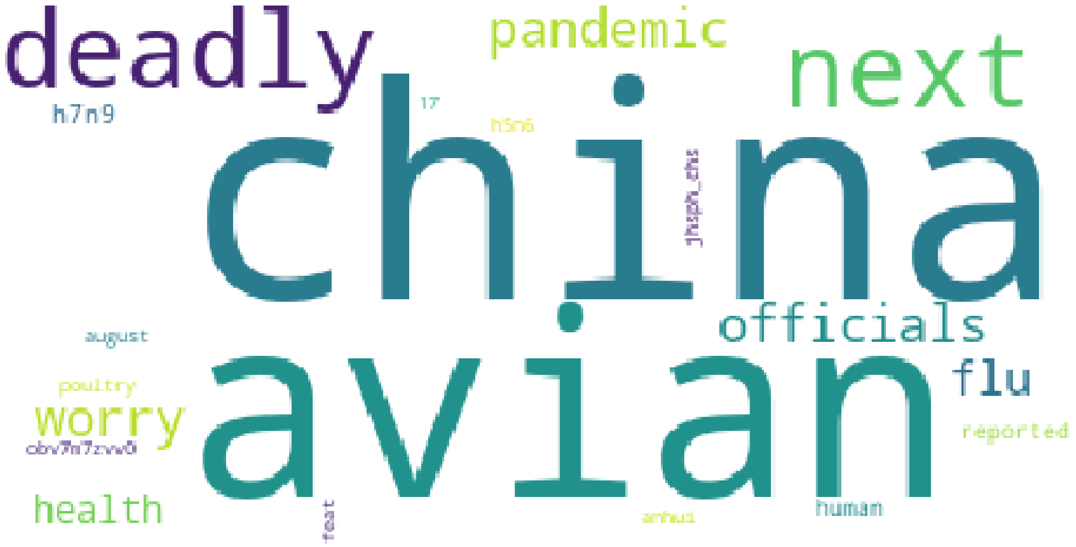

Figure 26. Word Clouds Around Outbreak Dates (China)

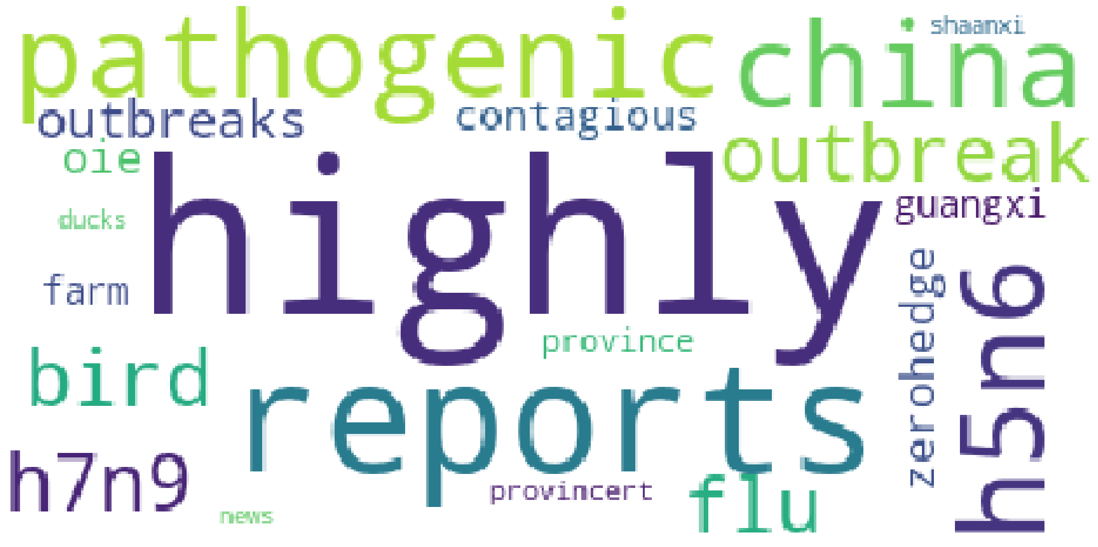

Figure 27. Word Clouds Around Outbreak Dates (China)

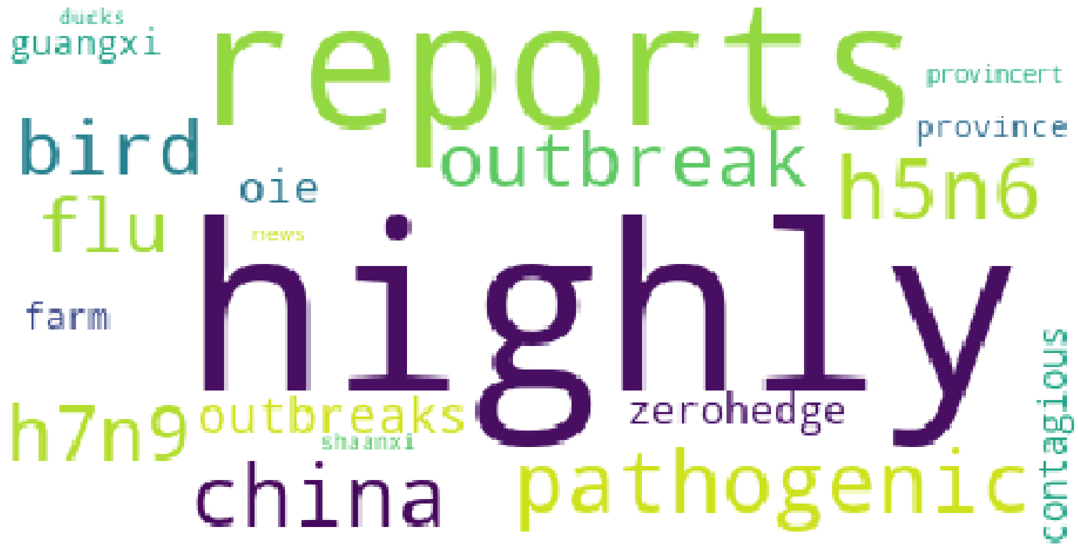

Figure 28. Word Clouds Around Outbreak Dates (China)

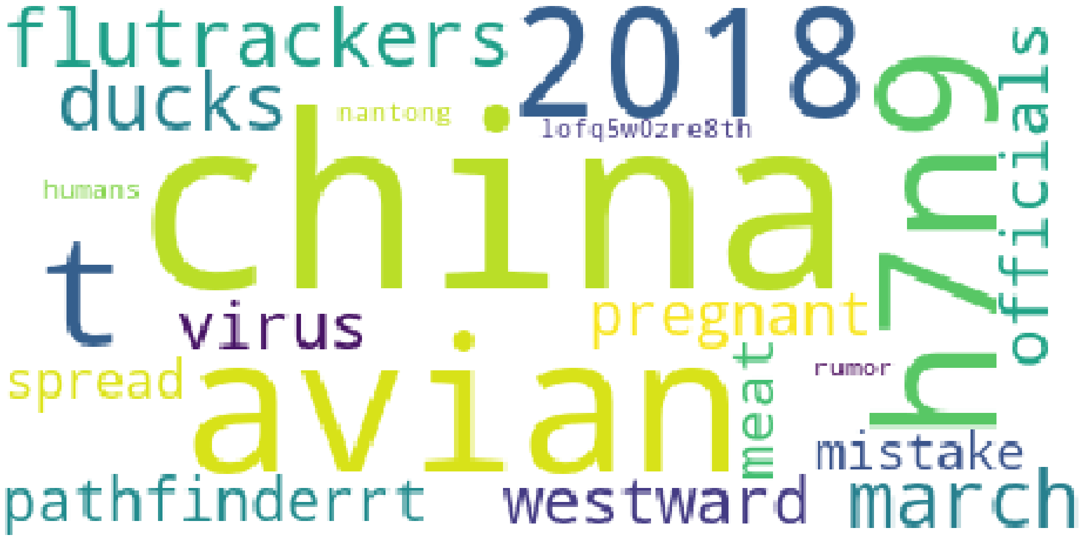

Figure 29. Word Clouds Around Outbreak Dates (China)

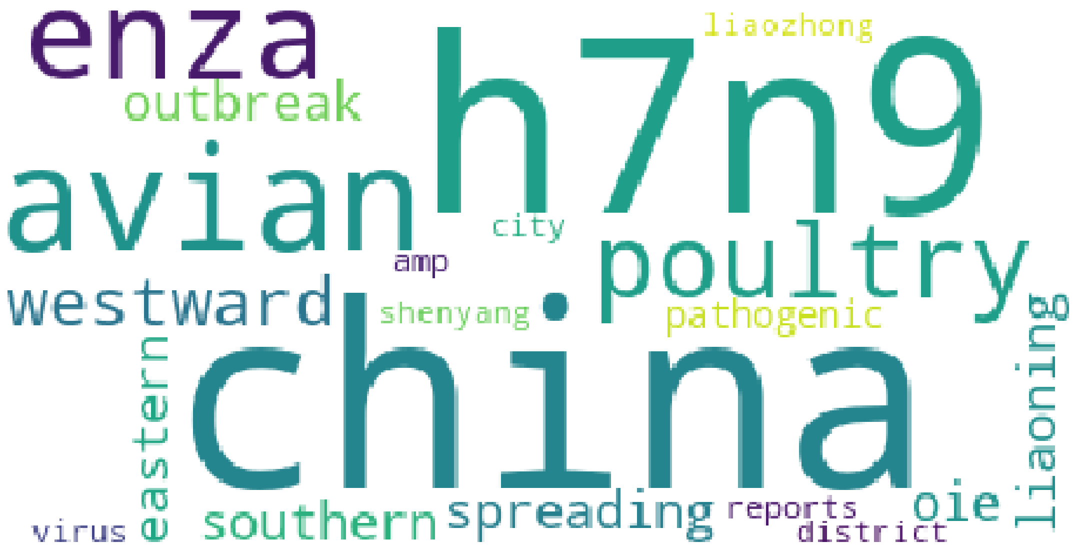

Figure 30. Word Clouds Around Outbreak Dates (China)

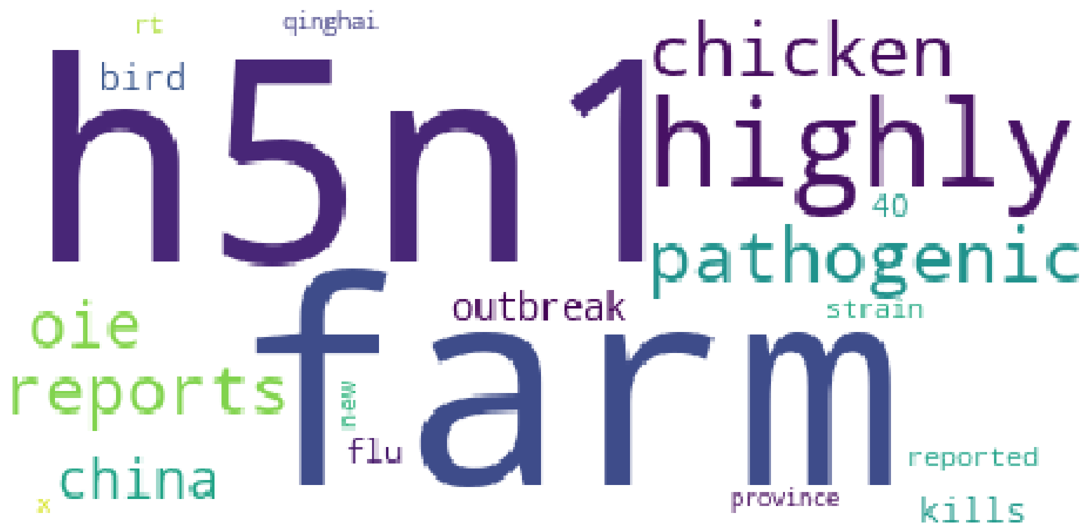

Figure 31. Word Clouds Around Outbreak Dates (China)

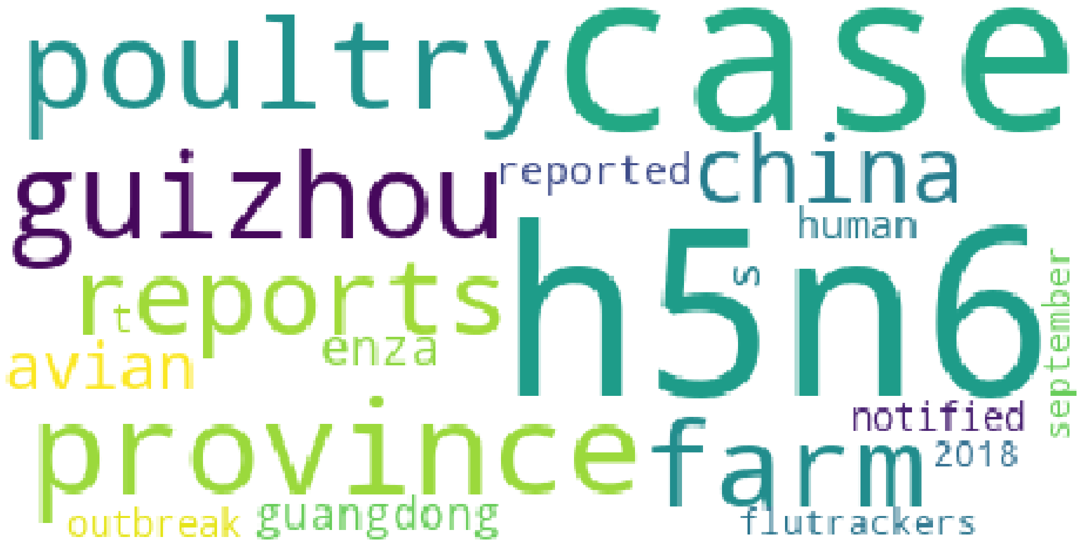

Figure 32. Word Clouds Around Outbreak Dates (China)

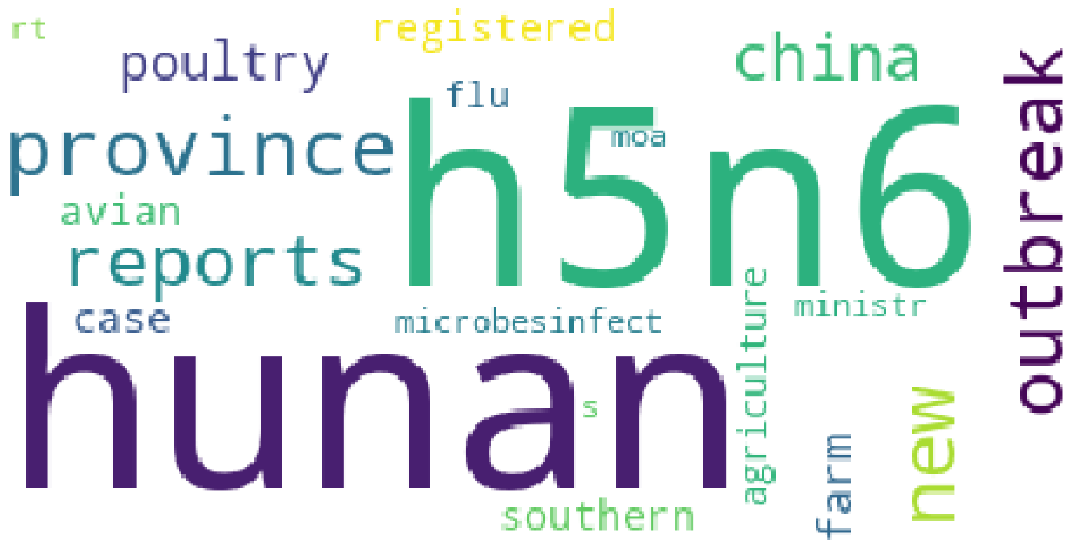

Figure 33. Word Clouds Around Outbreak Dates (China)

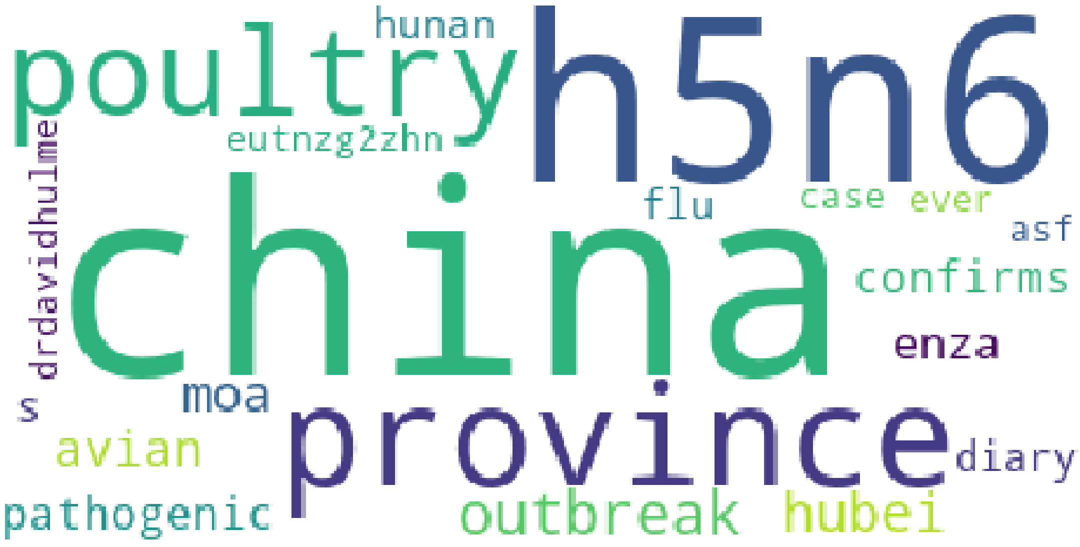

Figure 34. Word Clouds Around Outbreak Dates (China)

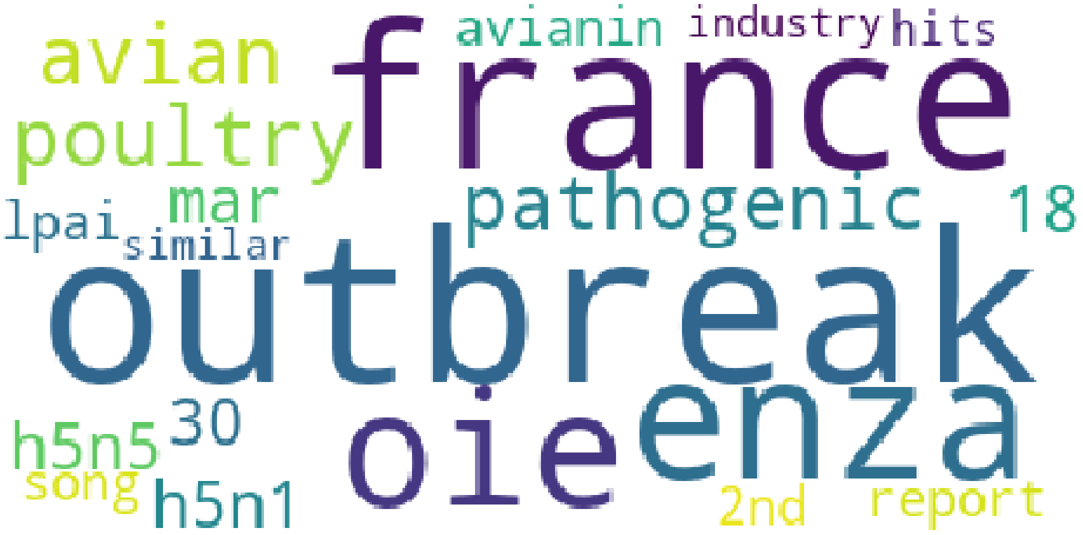

Figure 35. Word Clouds Around Outbreak Dates (France)

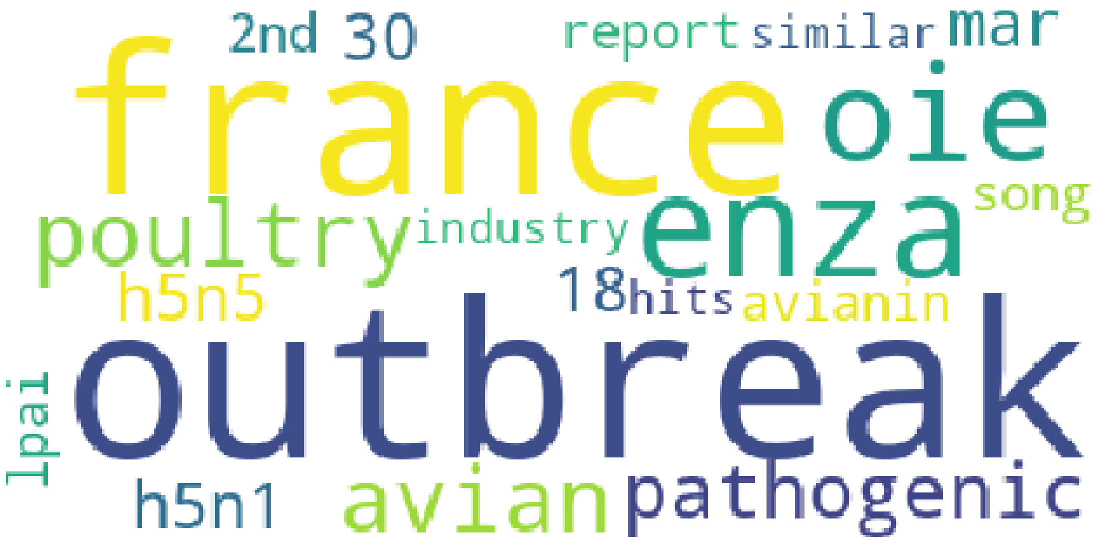

Figure 36. Word Clouds Around Outbreak Dates (France)

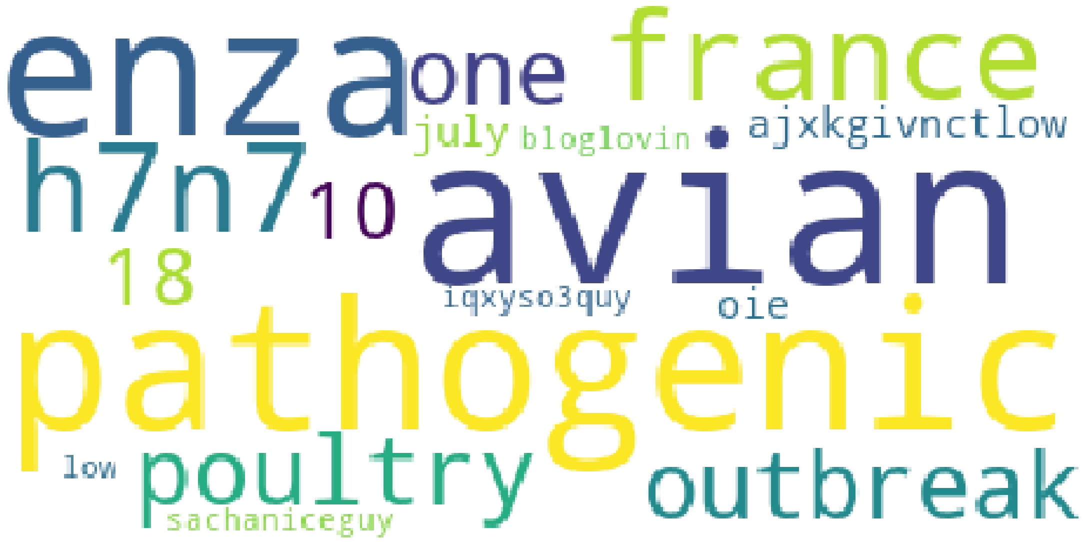

Figure 37. Word Clouds Around Outbreak Dates (France)

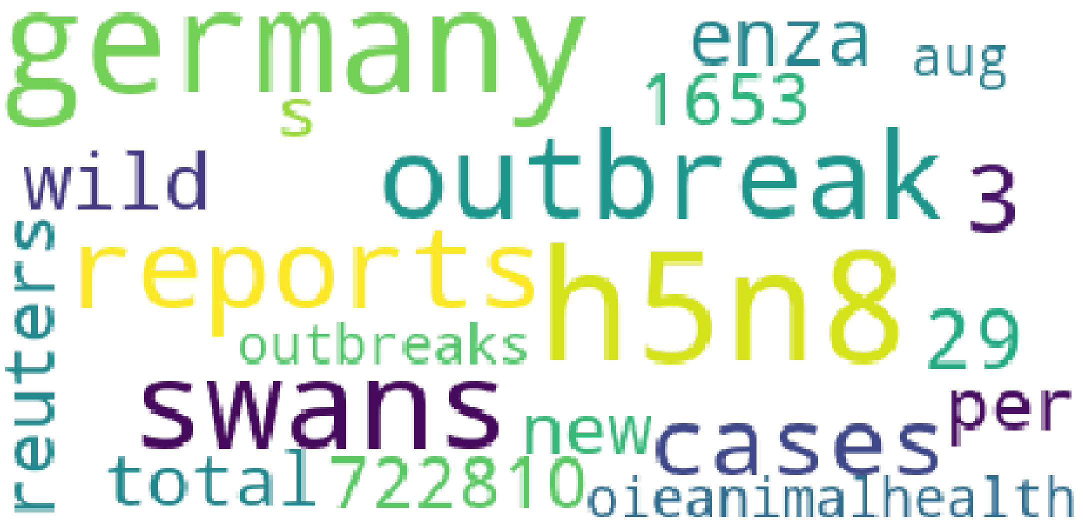

Figure 38. Word Clouds Around Outbreak Dates (Germany)

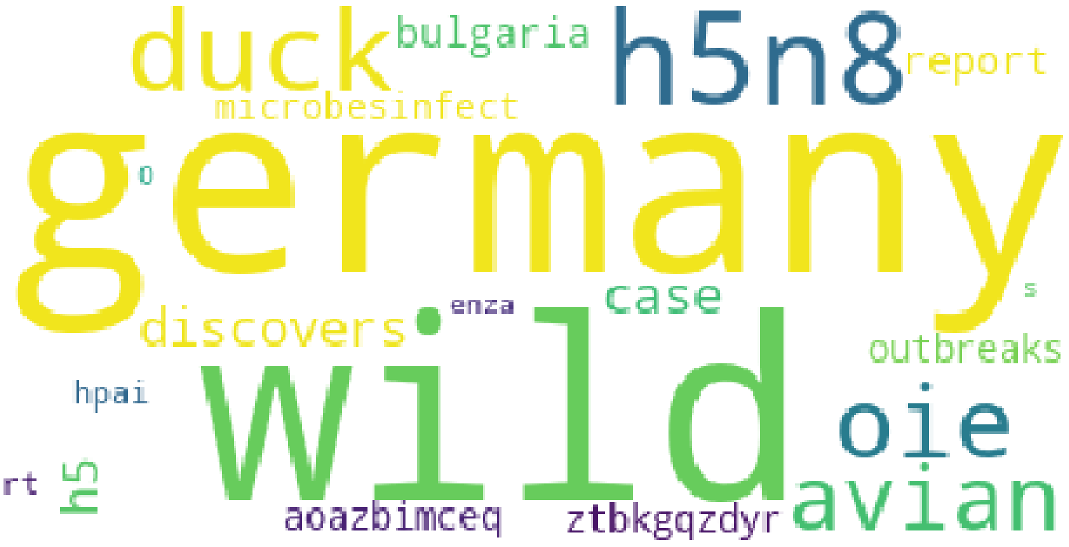

Figure 39. Word Clouds Around Outbreak Dates (Germany)

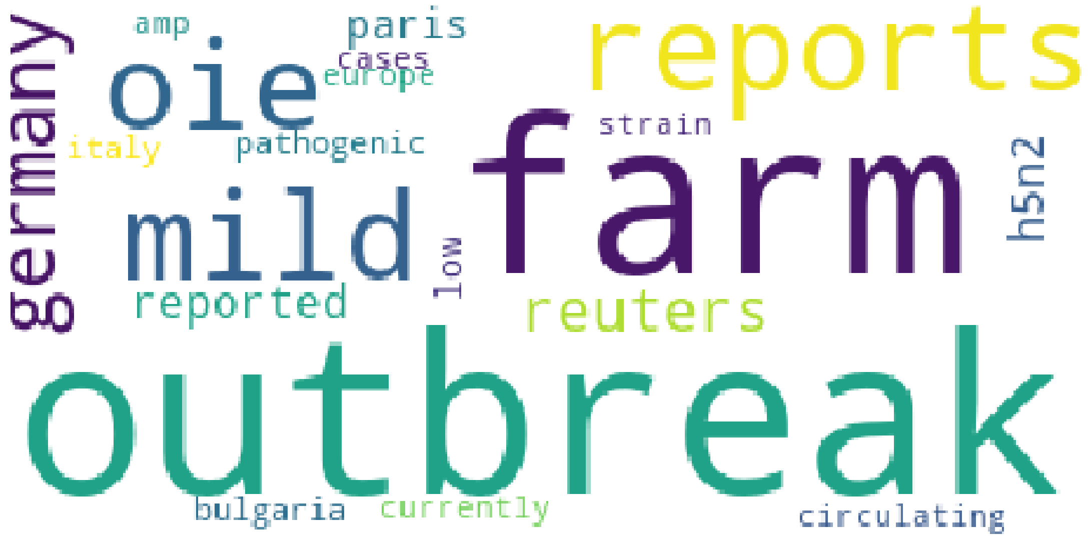

Figure 40. Word Clouds Around Outbreak Dates (Germany)

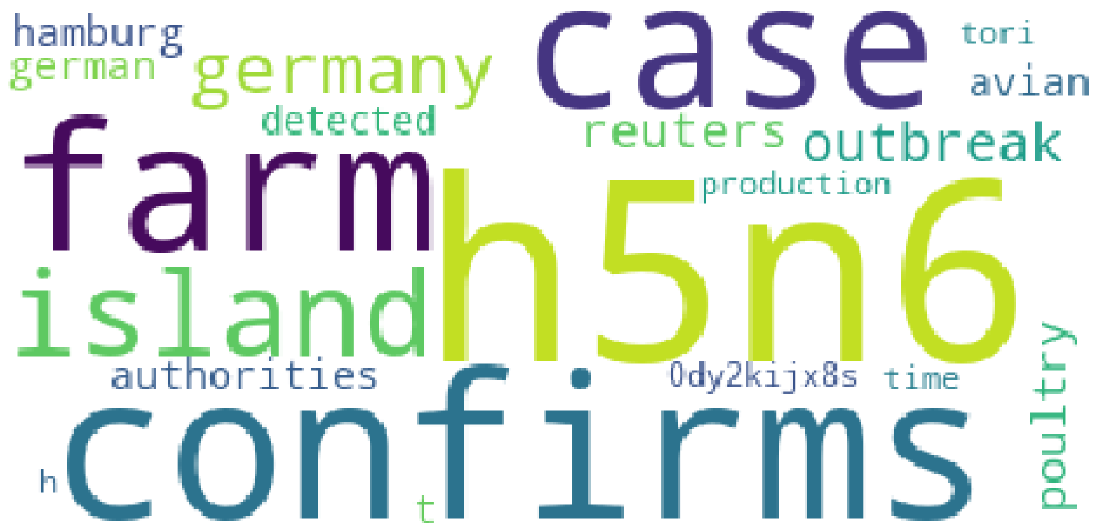

Figure 41. Word Clouds Around Outbreak Dates (Germany)

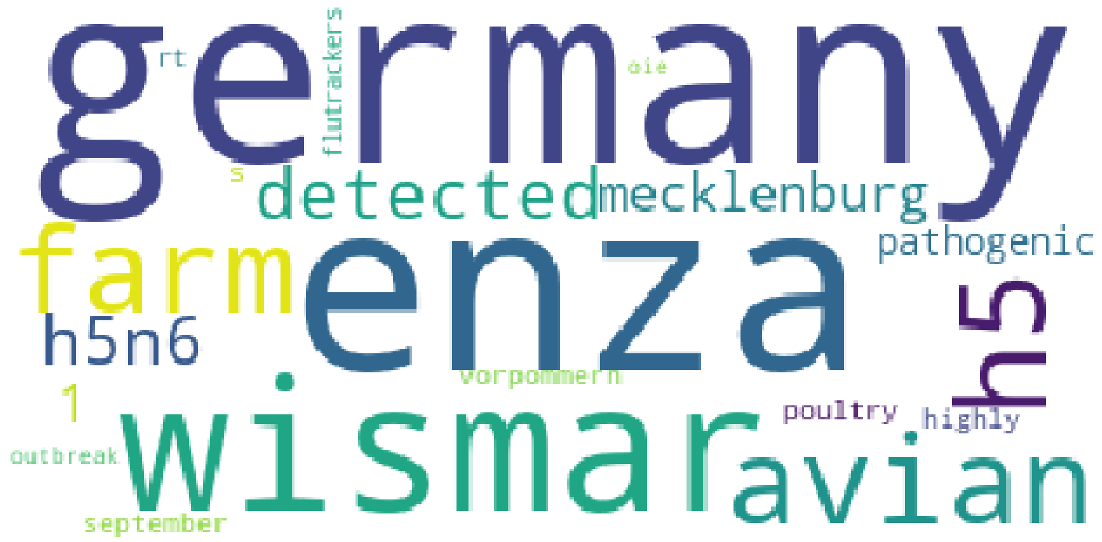

Figure 42. Word Clouds Around Outbreak Dates (Germany)

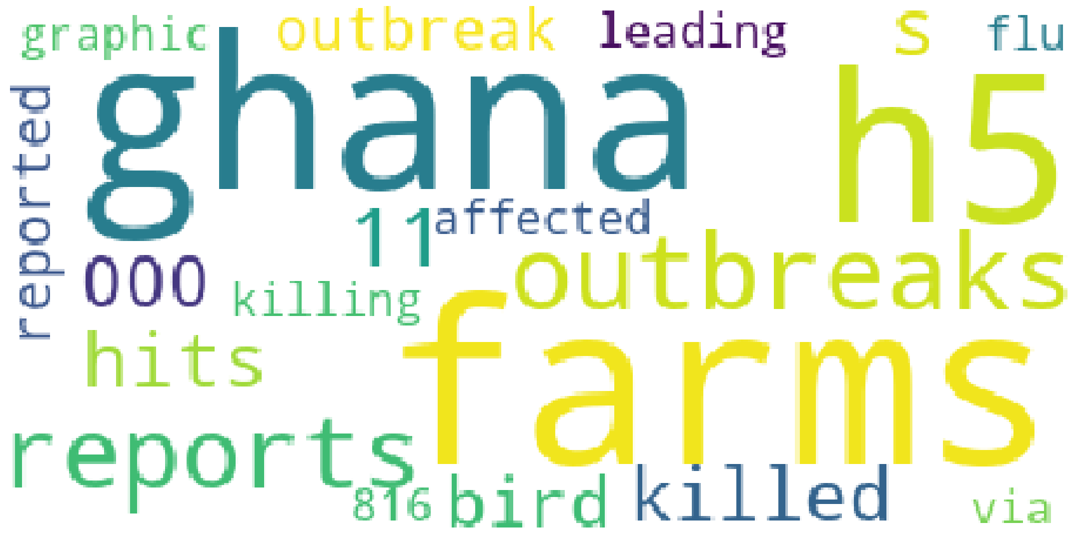

Figure 43. Word Clouds Around Outbreak Dates (Ghana)

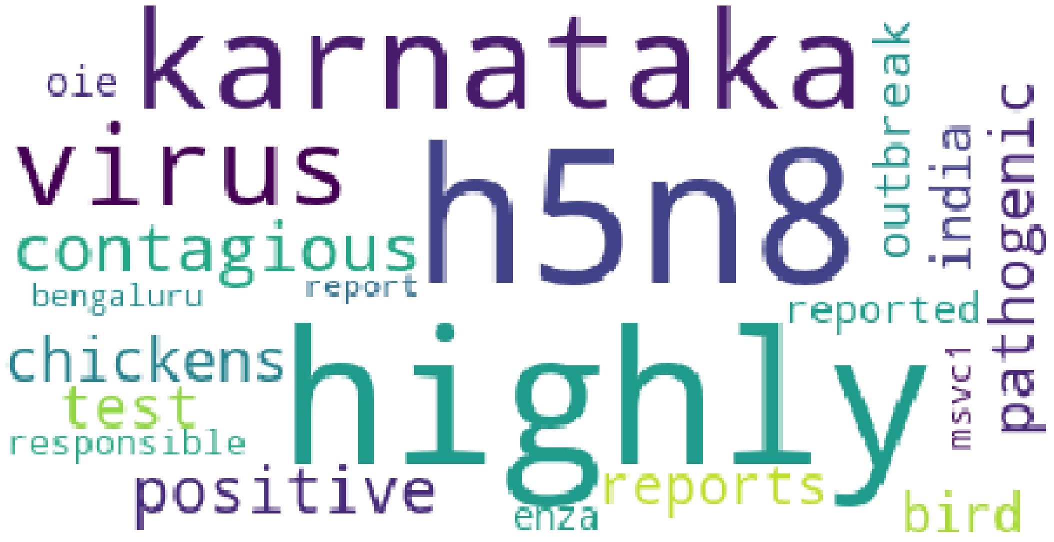

Figure 44. Word Clouds Around Outbreak Dates (India)

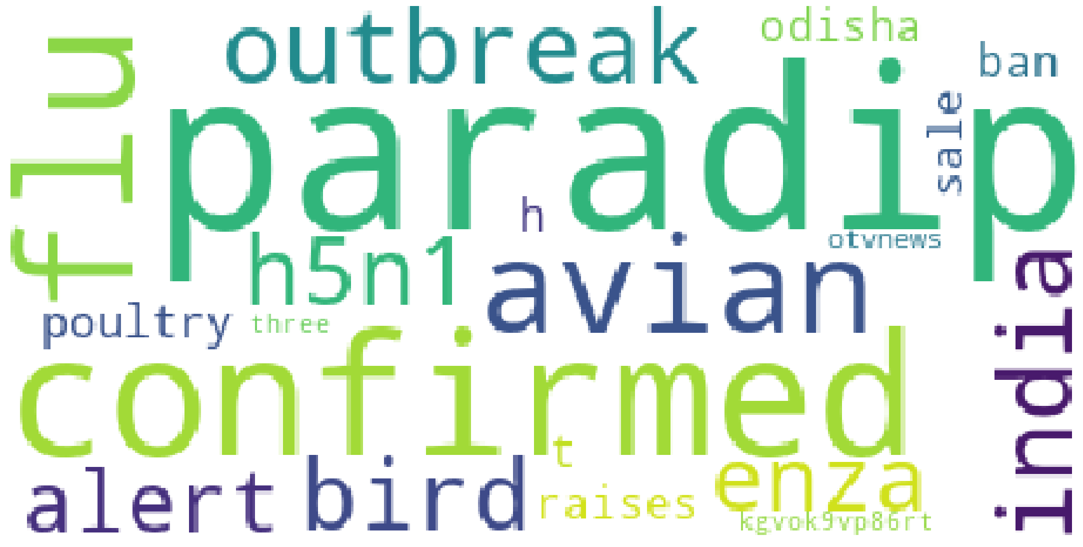

Figure 45. Word Clouds Around Outbreak Dates (India)

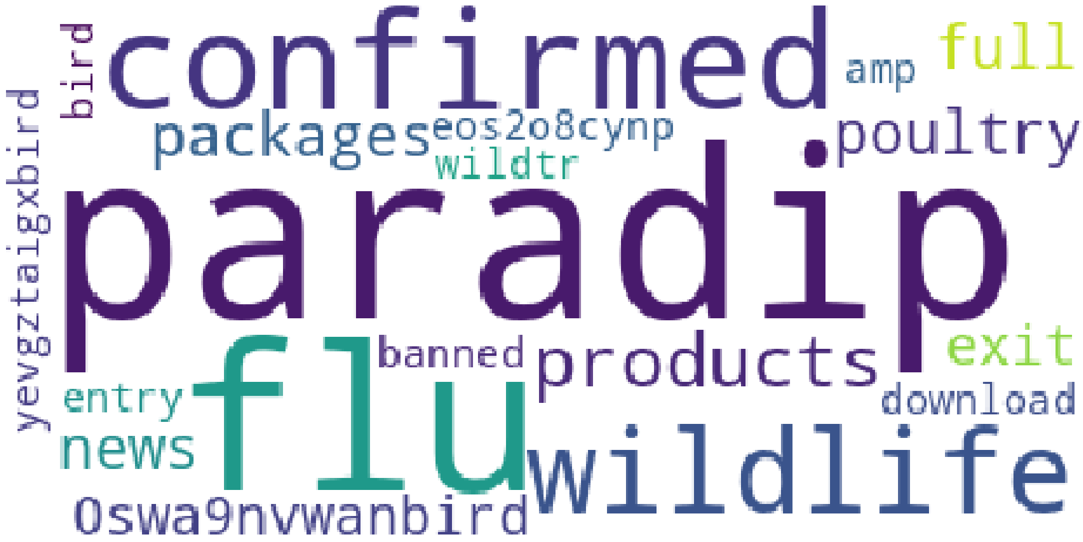

Figure 46. Word Clouds Around Outbreak Dates (India)

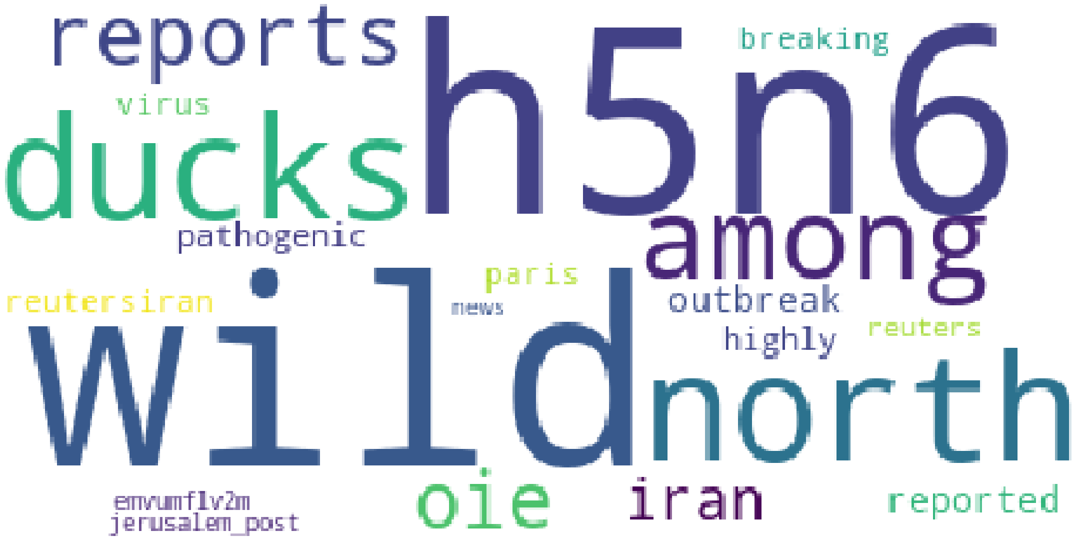

Figure 47. Word Clouds Around Outbreak Dates (Iran)

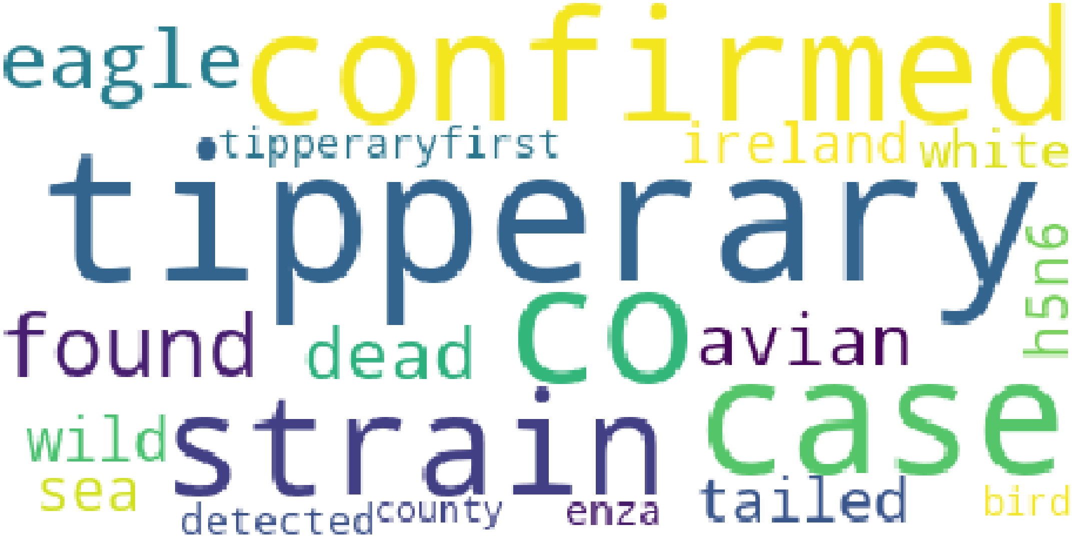

Figure 48. Word Clouds Around Outbreak Dates (Ireland)

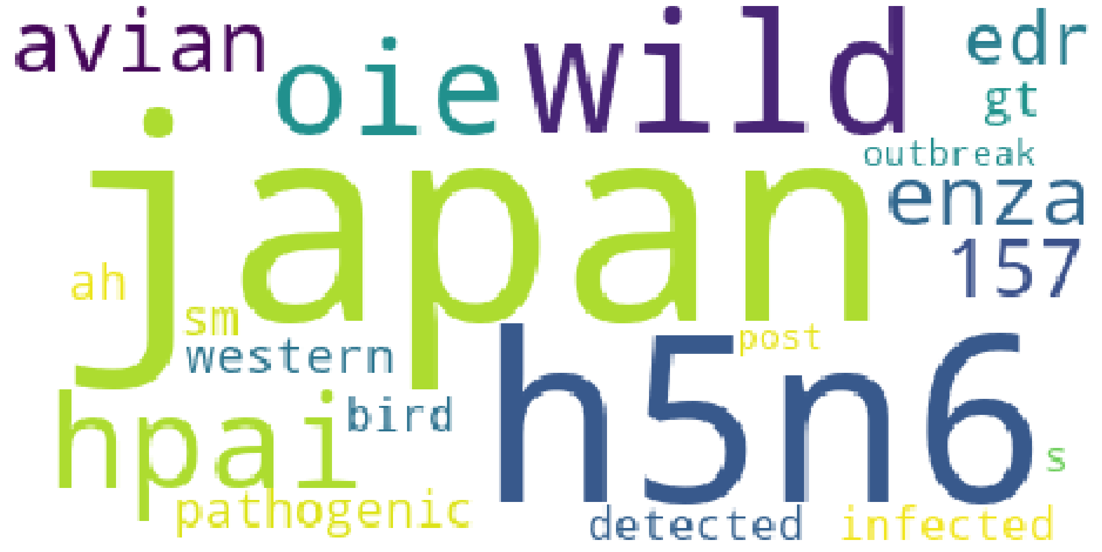

Figure 49. Word Clouds Around Outbreak Dates (Japan)

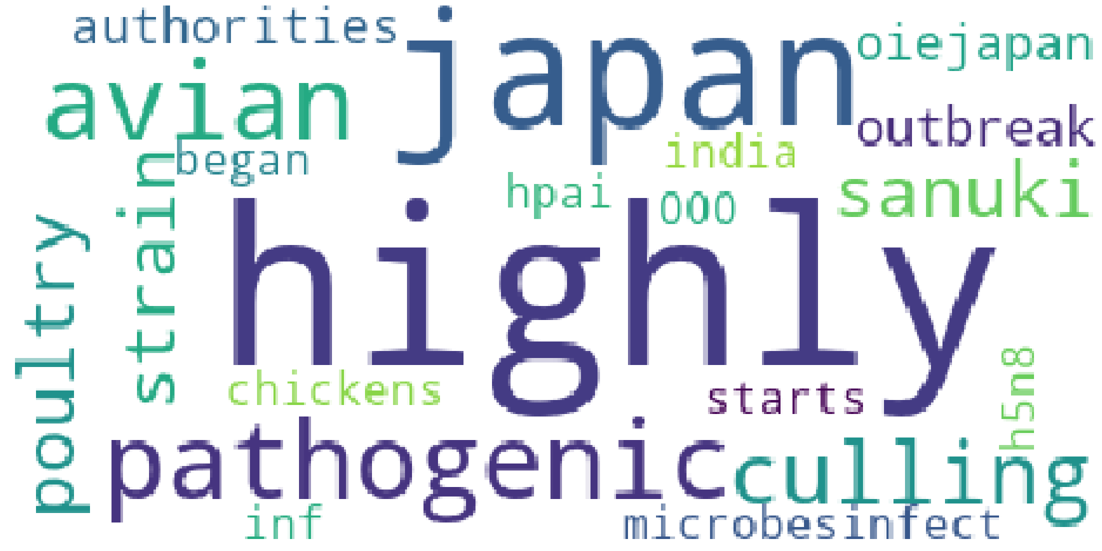

Figure 50. Word Clouds Around Outbreak Dates (Japan)

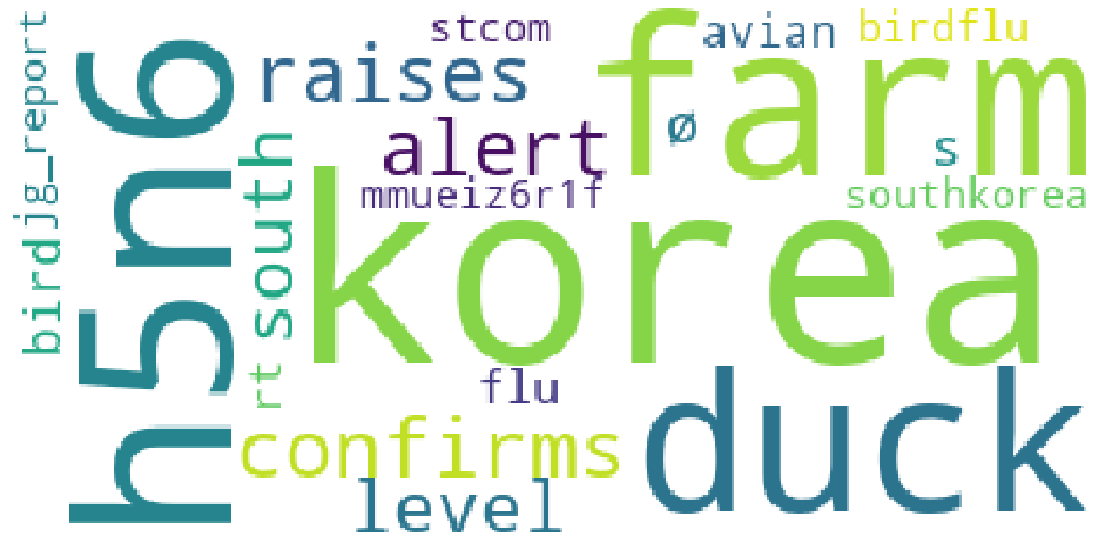

Figure 51. Word Clouds Around Outbreak Dates (Korea)

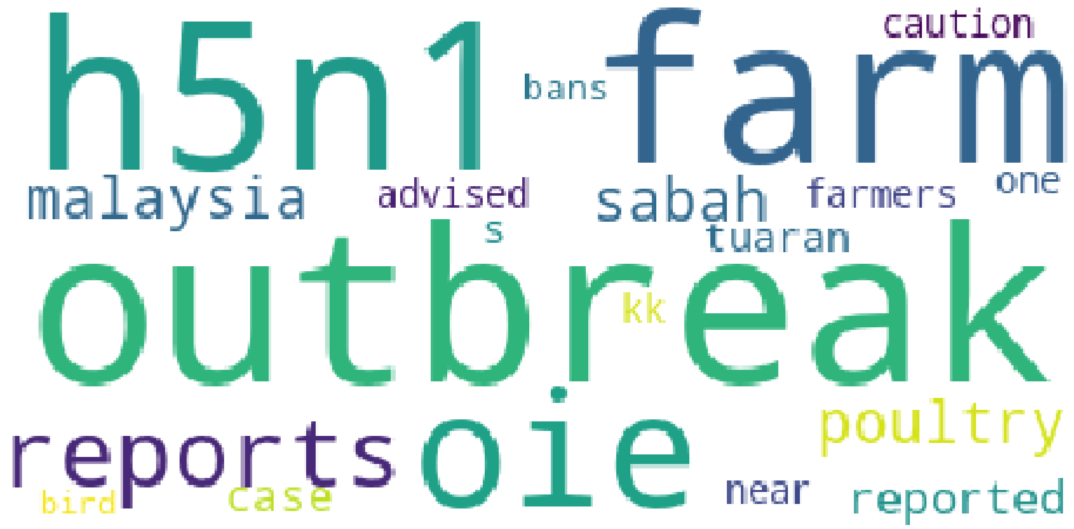

Figure 52. Word Clouds Around Outbreak Dates (Malaysia)

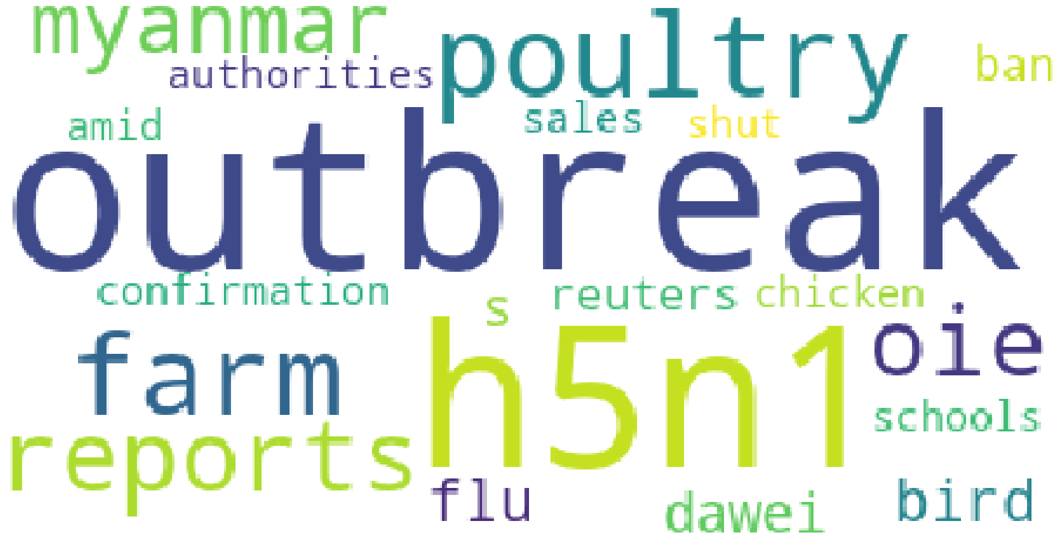

Figure 53. Word Clouds Around Outbreak Dates (Myanmar)

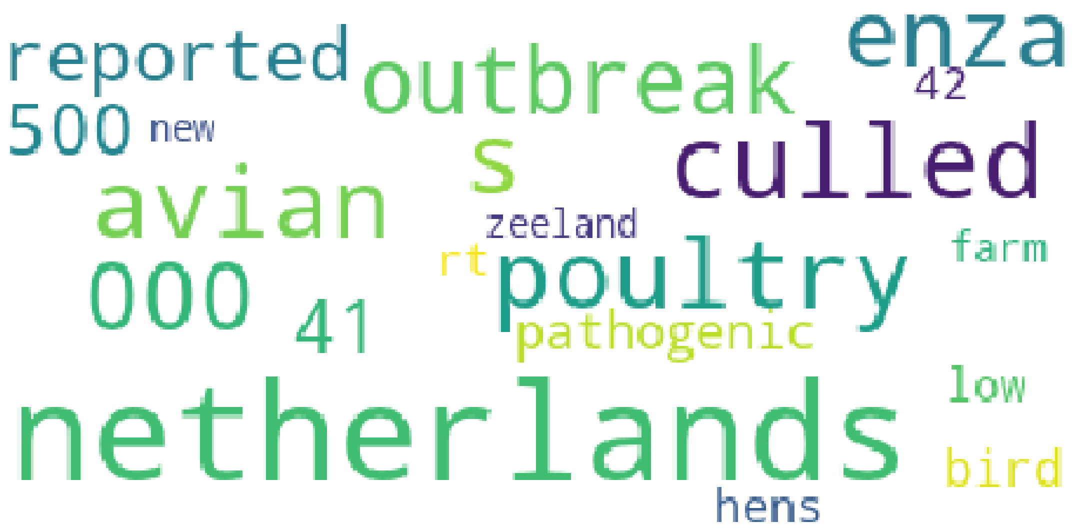

Figure 54. Word Clouds Around Outbreak Dates (The Netherlands)

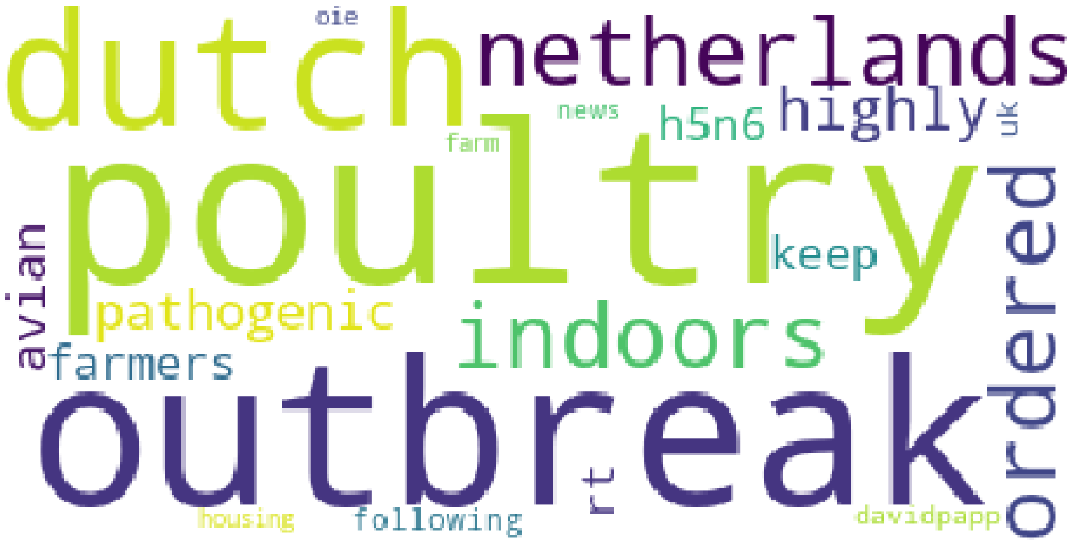

Figure 55. Word Clouds Around Outbreak Dates (The Netherlands)

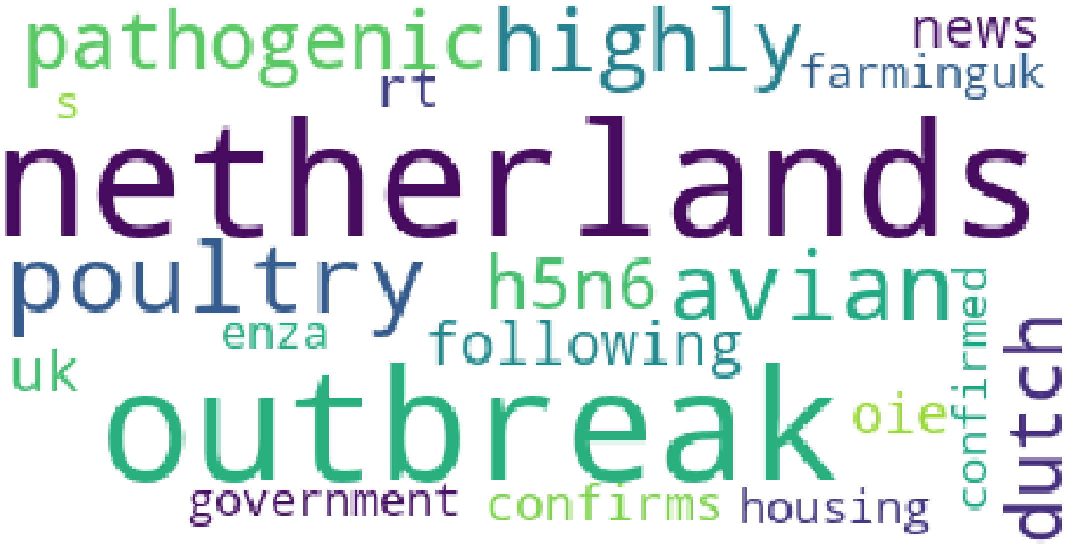

Figure 56. Word Clouds Around Outbreak Dates (The Netherlands)

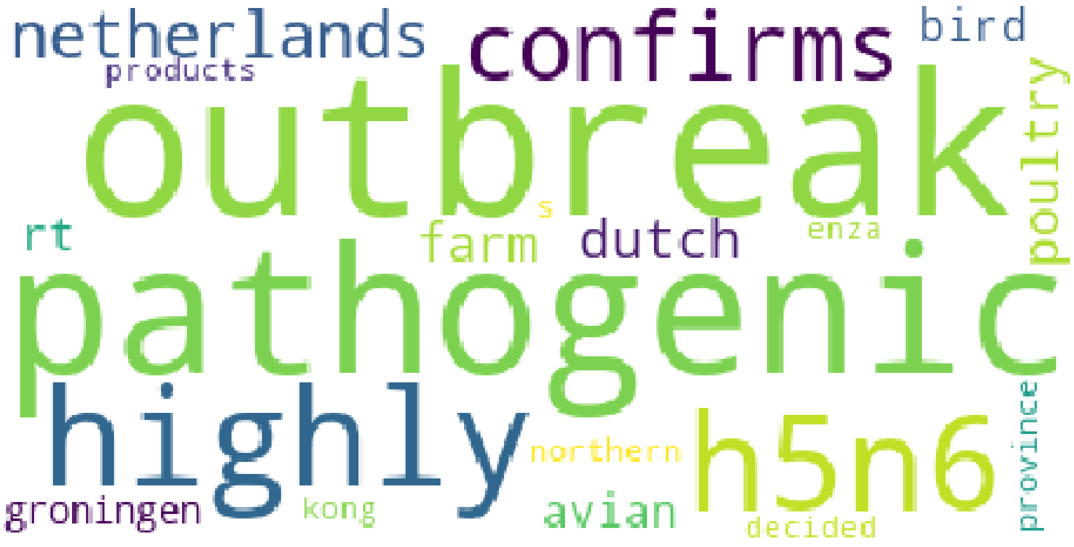

Figure 57. Word Clouds Around Outbreak Dates (The Netherlands)

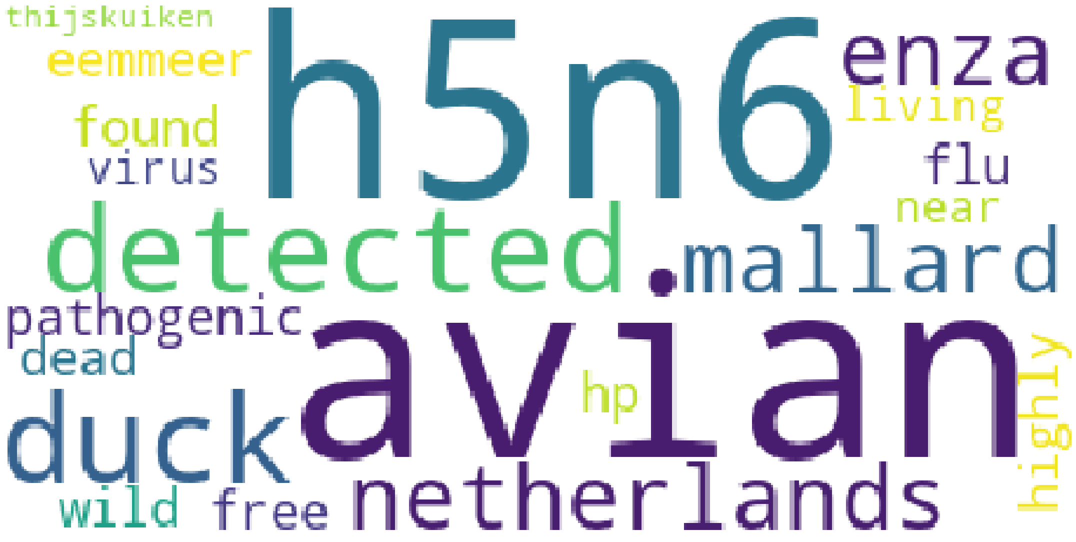

Figure 58. Word Clouds Around Outbreak Dates (The Netherlands)

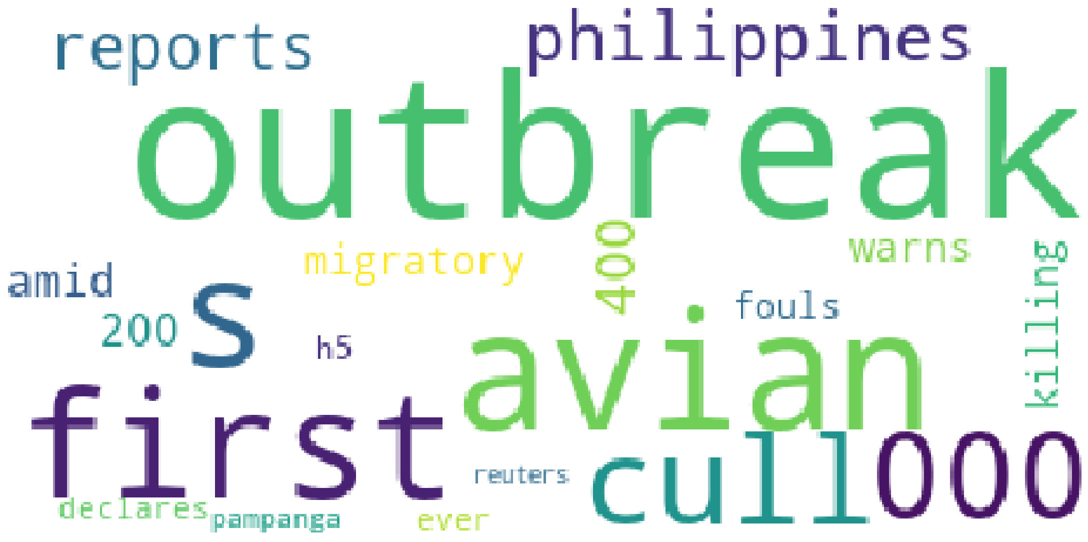

Figure 59. Word Clouds Around Outbreak Dates (The Philippines)

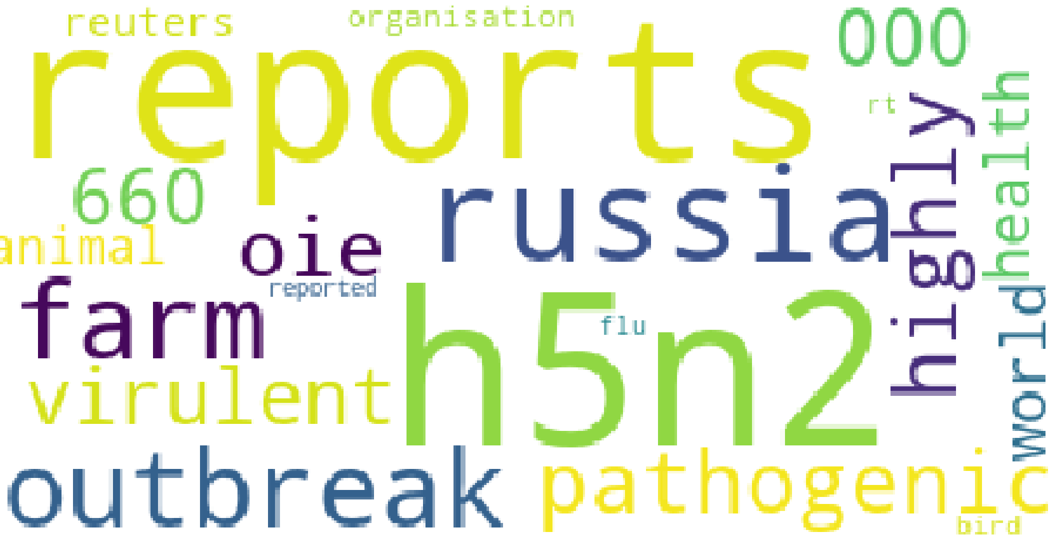

Figure 60. Word Clouds Around Outbreak Dates (Russia)

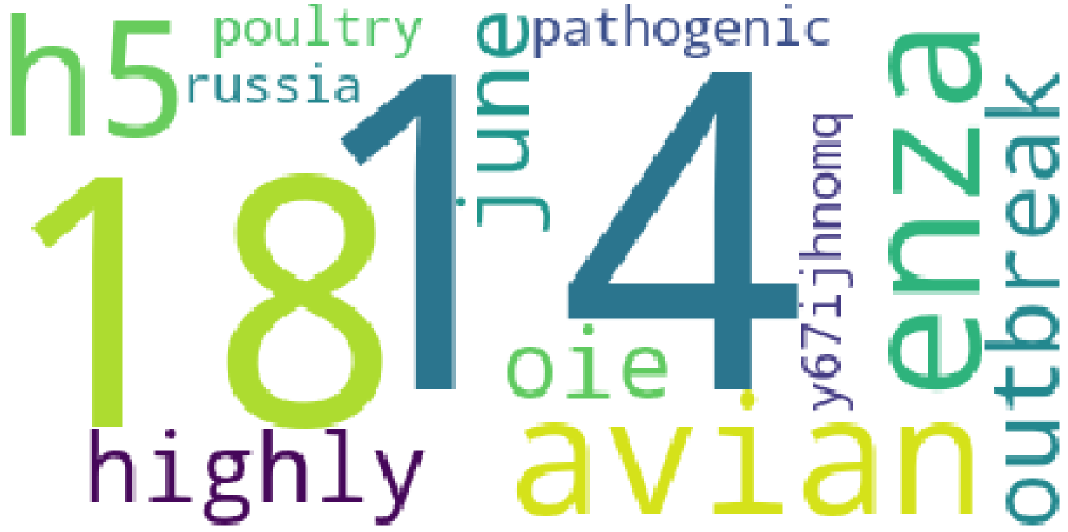

Figure 61. Word Clouds Around Outbreak Dates (Russia)

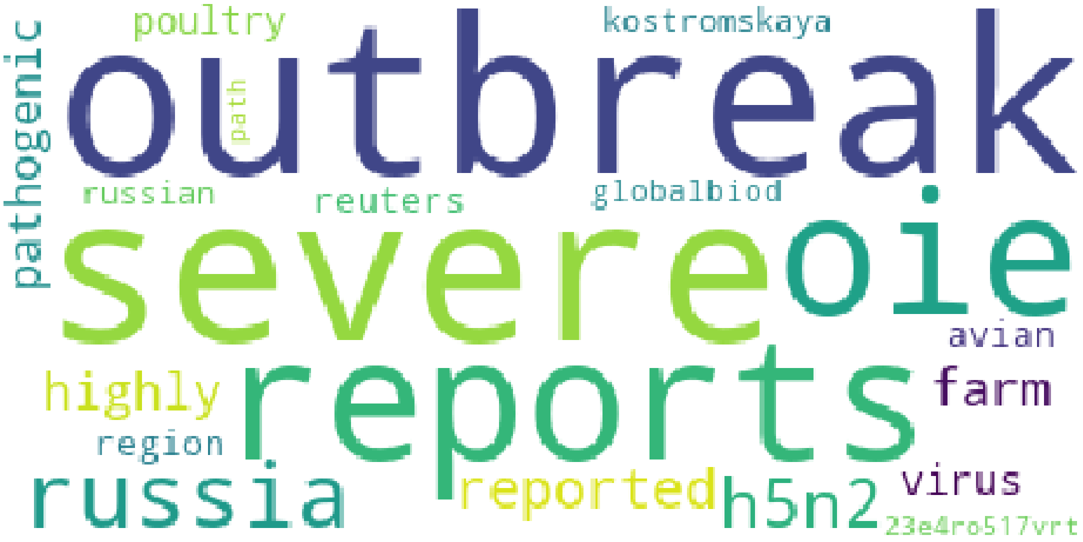

Figure 62. Word Clouds Around Outbreak Dates (Russia)

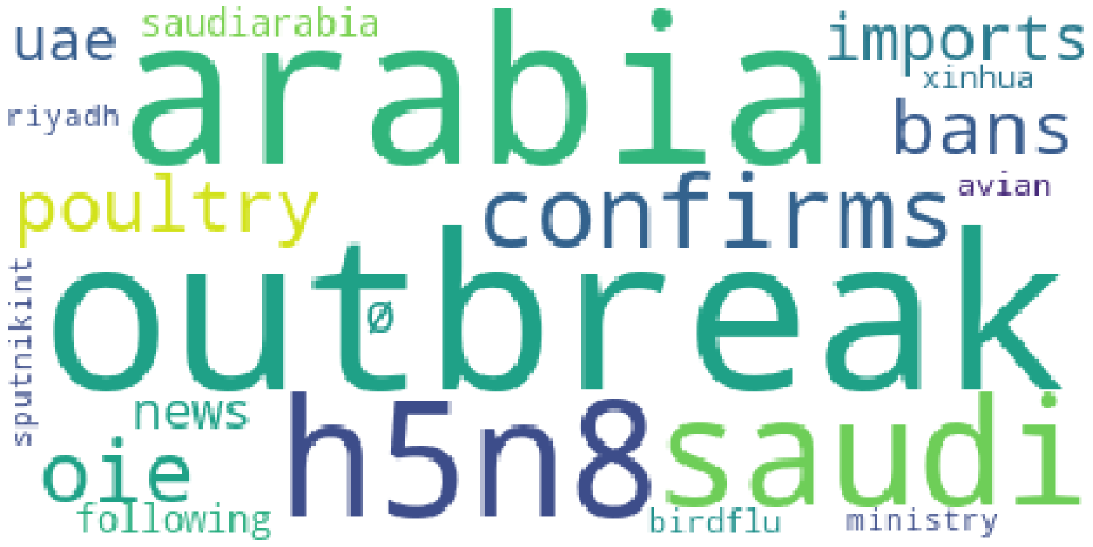

Figure 63. Word Clouds Around Outbreak Dates (Saudi Arabia)

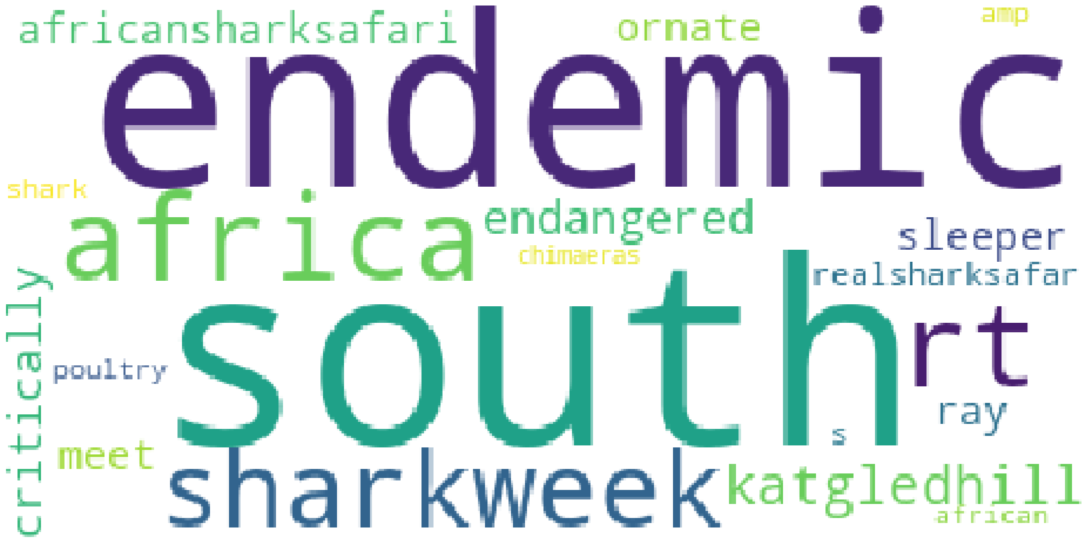

Figure 64. Word Clouds Around Outbreak Dates (South Africa)

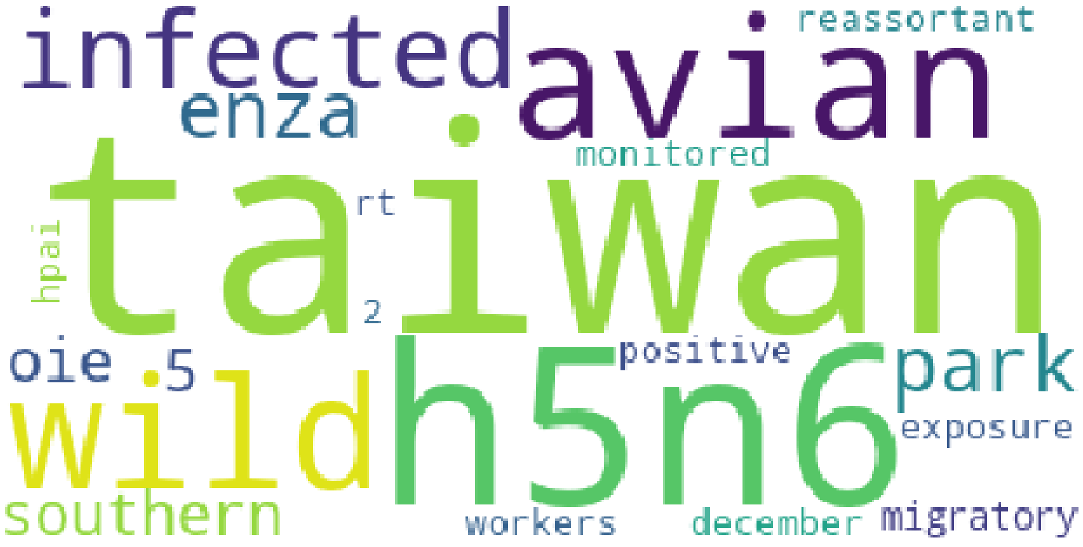

Figure 65. Word Clouds Around Outbreak Dates (Taiwan)

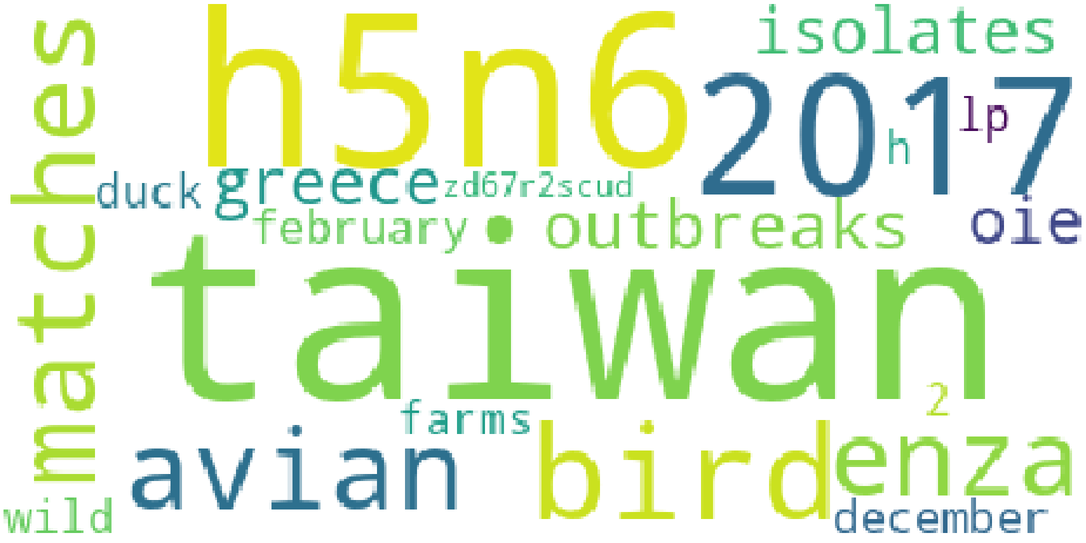

Figure 66. Word Clouds Around Outbreak Dates (Taiwan)

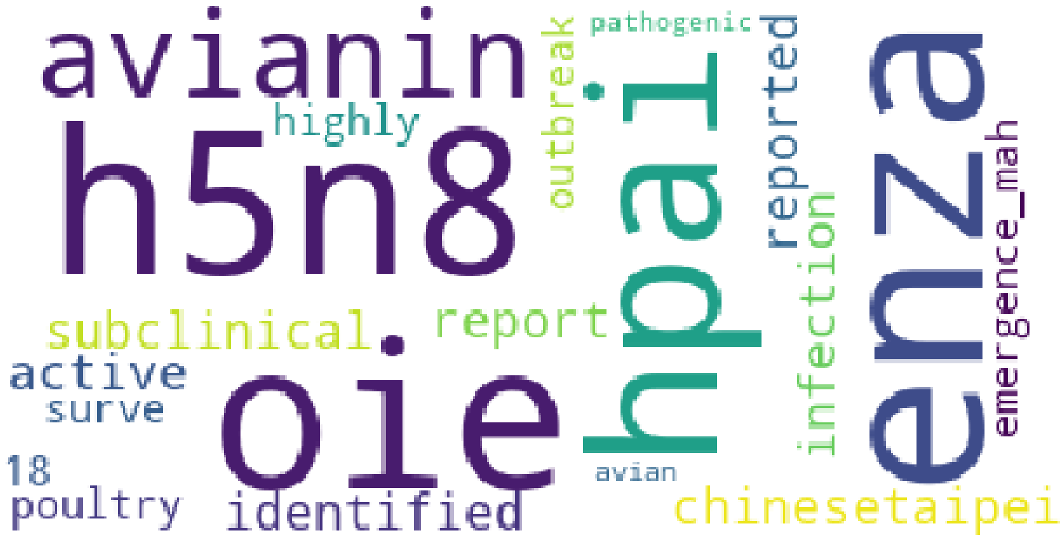

Figure 67. Word Clouds Around Outbreak Dates (Taiwan)

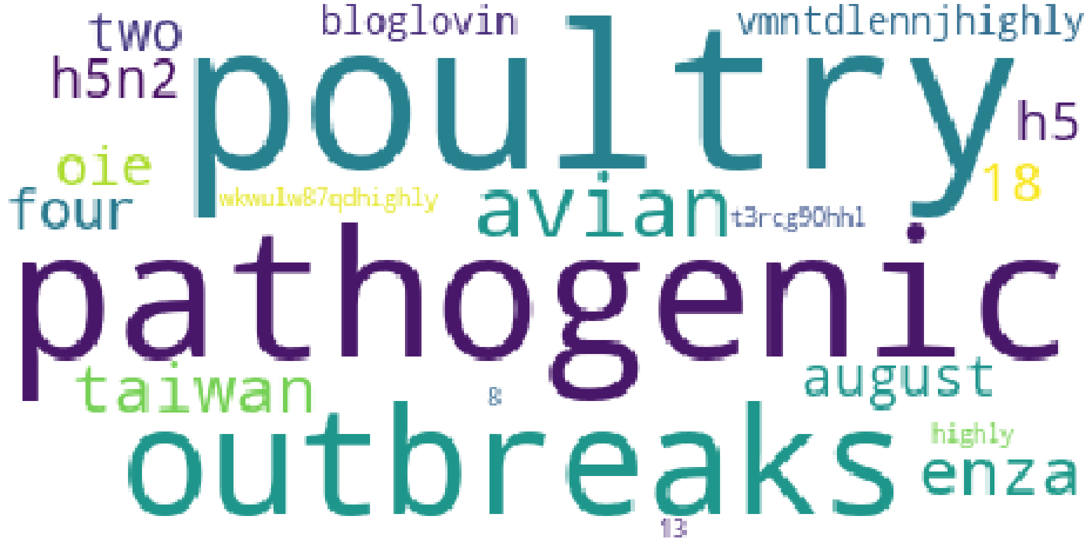

Figure 68. Word Clouds Around Outbreak Dates (Taiwan)

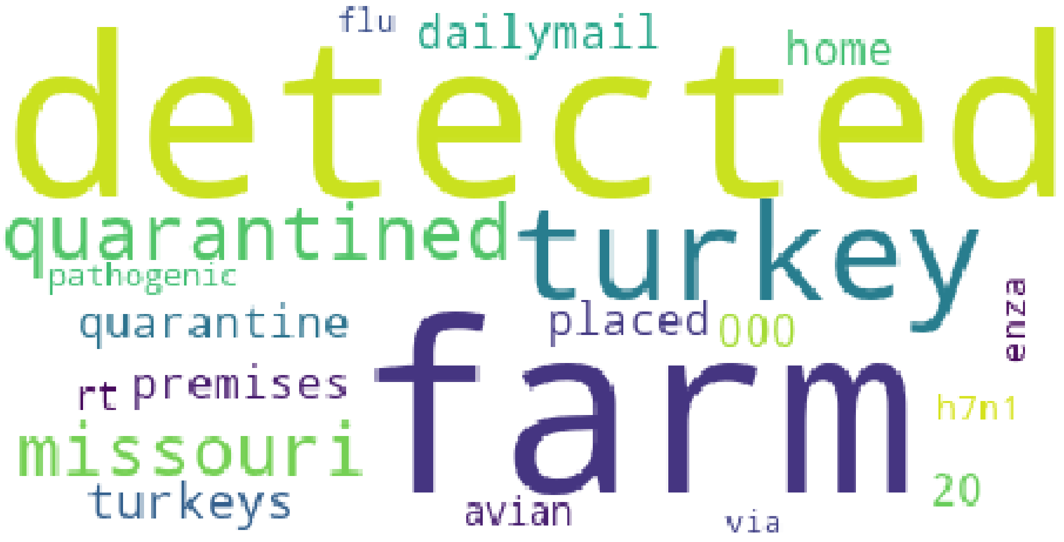

Figure 69. Word Clouds Around Outbreak Dates (USA)

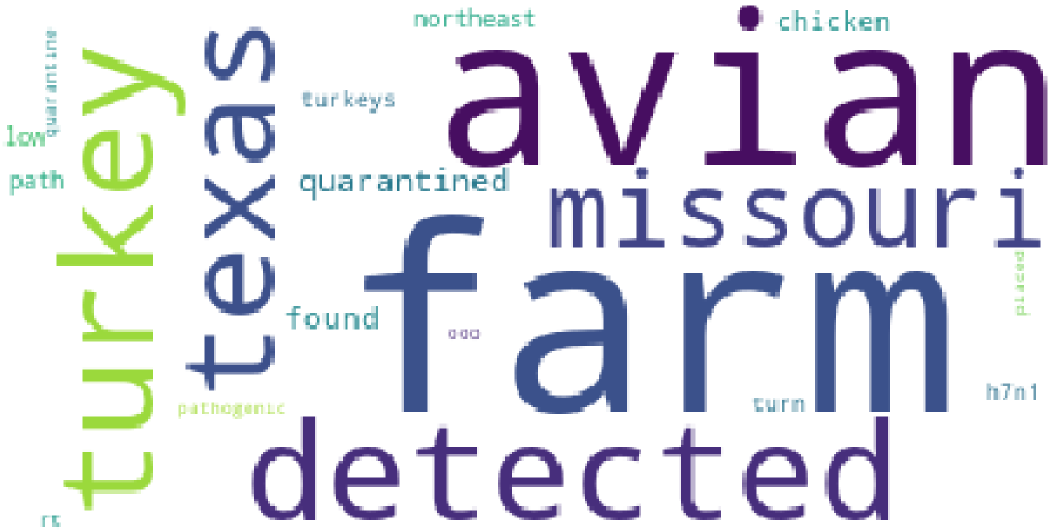

Figure 70. Word Clouds Around Outbreak Dates (USA)

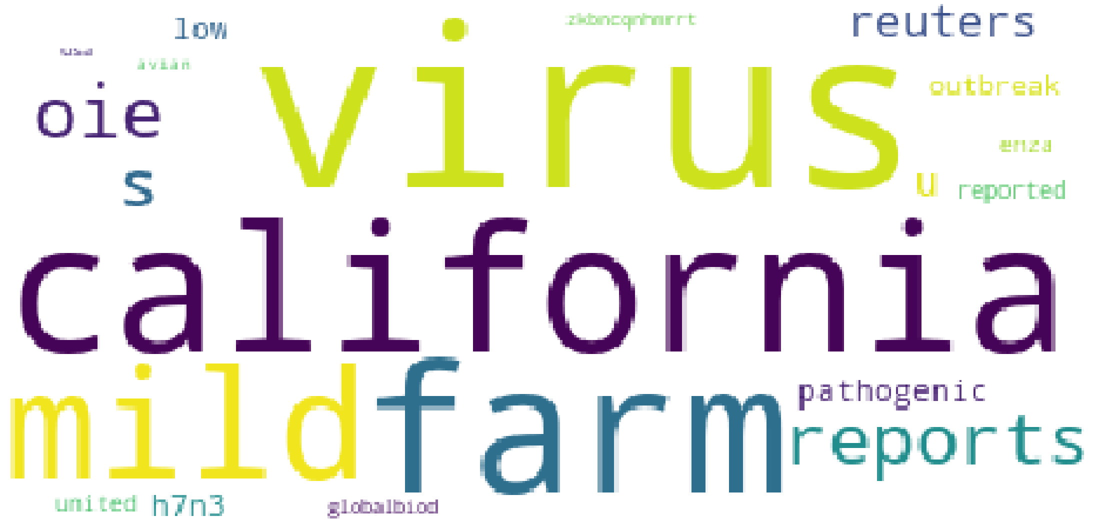

Figure 71. Word Clouds Around Outbreak Dates (USA)

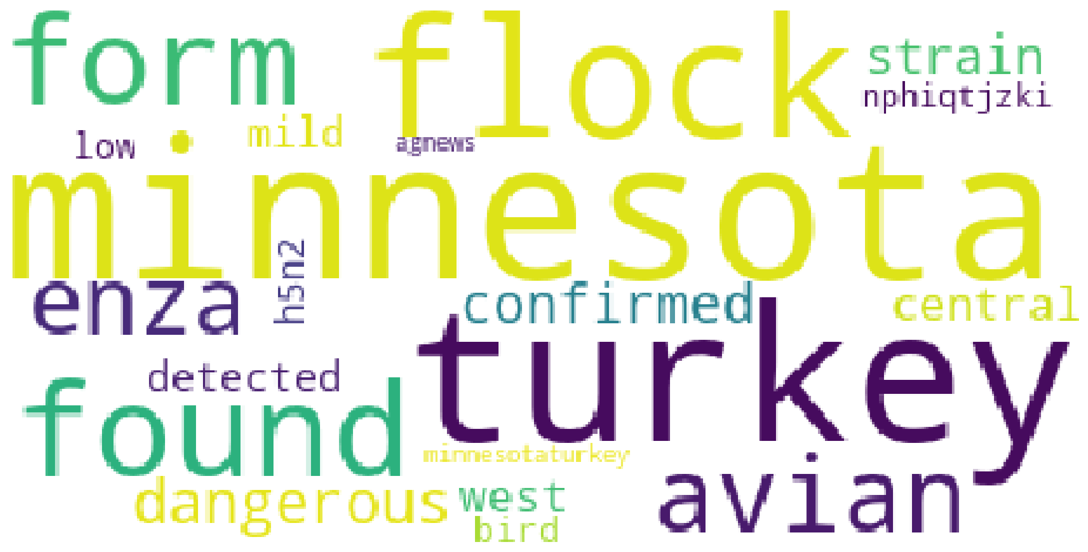

Figure 72. Word Clouds Around Outbreak Dates (USA)

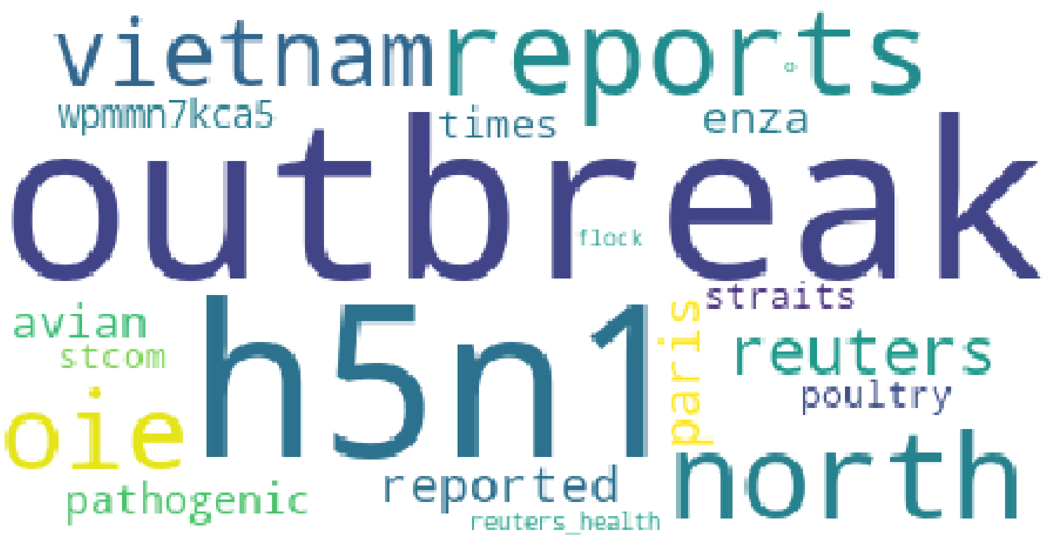

Figure 73. Word Clouds Around Outbreak Dates (Vietnam)

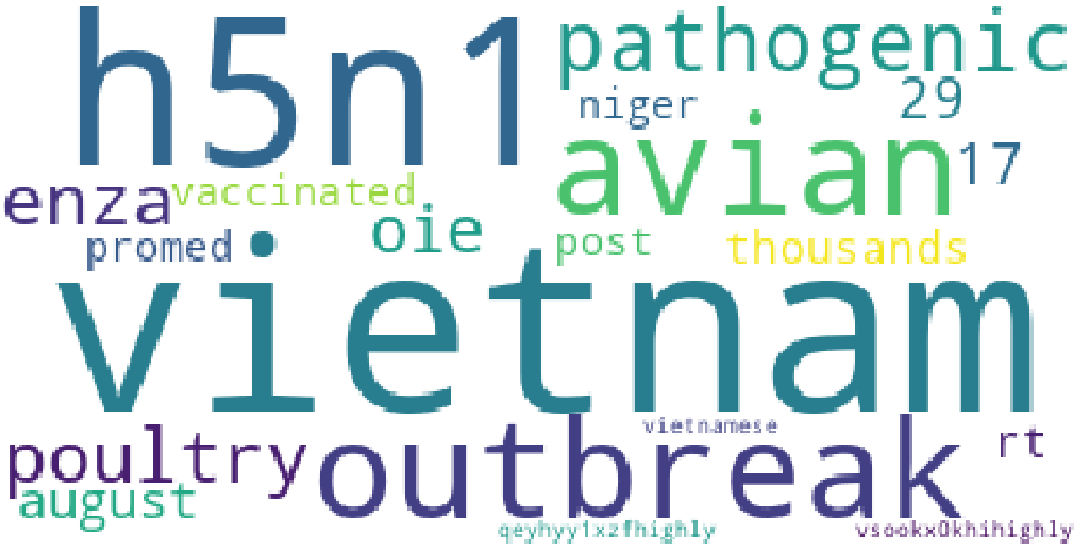

Figure 74. Word Clouds Around Outbreak Dates (Vietnam)

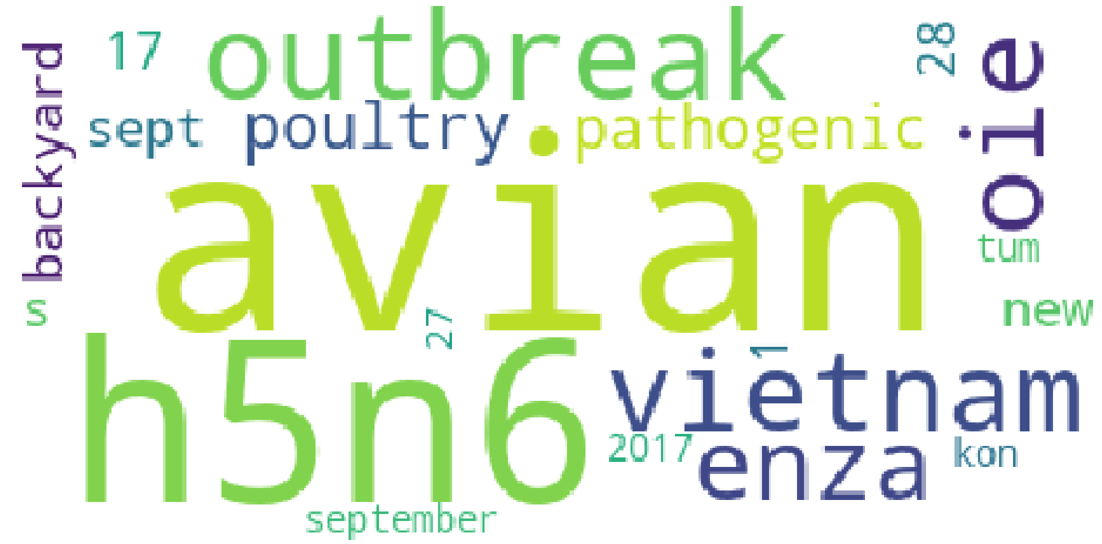

Figure 75. Word Clouds Around Outbreak Dates (Vietnam)

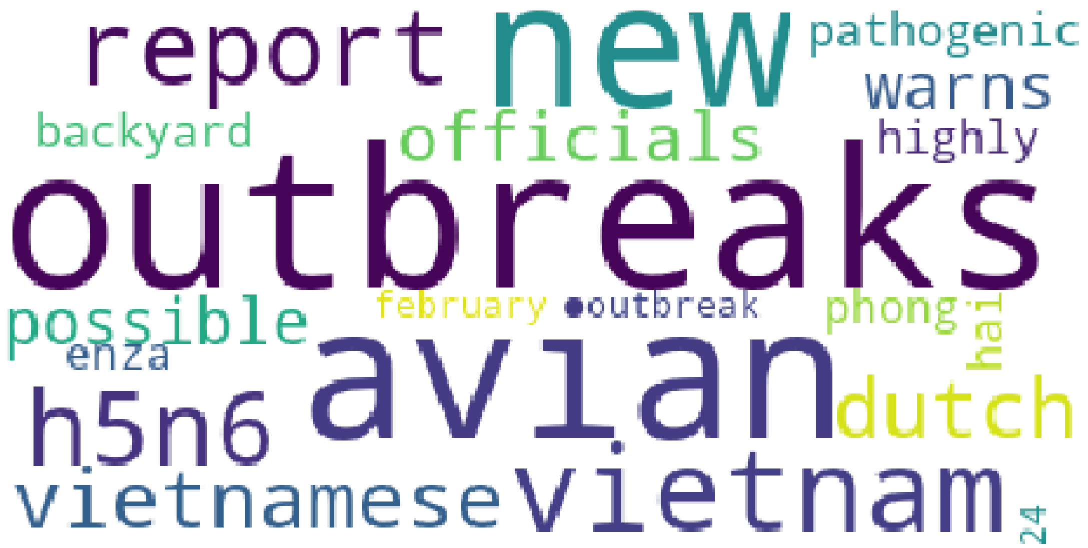

Figure 76. Word Clouds Around Outbreak Dates (Vietnam)

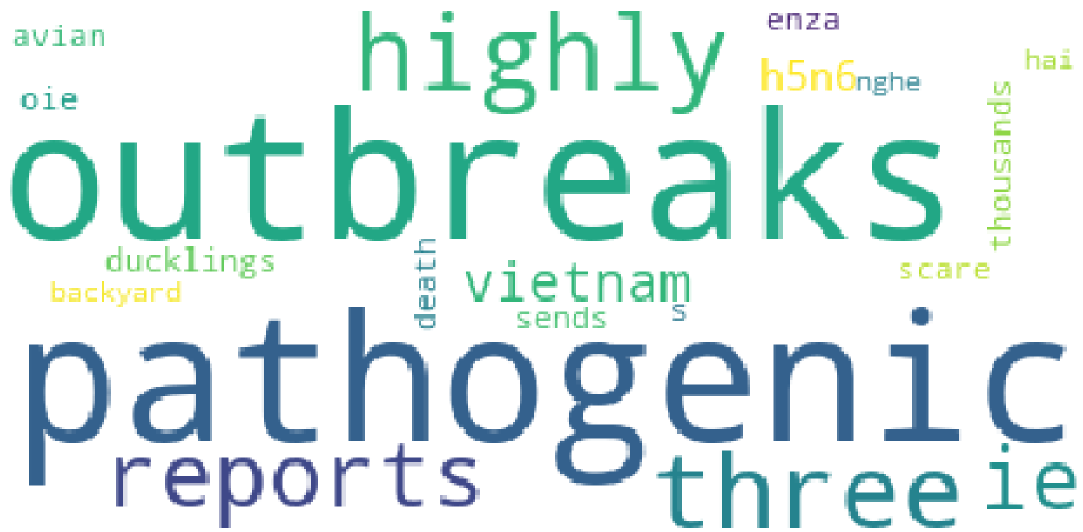

Figure 77. Word Clouds Around Outbreak Dates (Vietnam)
